# Supplementary material for: Feature extraction tool using temporal landmarks in arterial blood pressure and photoplethysmography waveforms
Source: NPJ Cardiovasc Health. 2025 Nov 24;2:57. doi: 10.1038/s44325-025-00096-0 (PMC12643918; doi:10.1038/s44325-025-00096-0)
Supplement: Supplementary file 1 — Supplementary Movie1 [file 44325_2025_96_MOESM1_ESM.pdf]

Supplementary Movie1. Demonstration video. Showcases two examples of photoplethysmography (PPG) waveforms and two examples of arterial blood pressure (ABP) waveforms.
