# Supplementary material for: Feature extraction tool using temporal landmarks in arterial blood pressure and photoplethysmography waveforms
Source: NPJ Cardiovasc Health. 2025 Nov 24;2:57. doi: 10.1038/s44325-025-00096-0 (PMC12643918; doi:10.1038/s44325-025-00096-0)
Supplement: Supplementary file 3 — Supplementary table [file 44325_2025_96_MOESM3_ESM.pdf]

Detailed descriptions of the features (n1=852) that the feature extraction tool can extract from arterial blood pressure (ABP) and photoplethysmography (PPG) waveforms per cardiac cycle

1. Original and normalized amplitude features (1-30)

1	ABP/PPG_AM_SPO_wrtZero	ABP/PPG cardiac cycle systolic phase onset amplitude with respect to zero
2	ABP/PPG_AM_SPP_wrtZero	ABP/PPG cardiac cycle systolic phase peak amplitude with respect to zero
3	ABP/PPG_AM_DN_wrtZero	ABP/PPG cardiac cycle dicrotic notch amplitude with respect to zero
4	ABP/PPG_AM_DPP_wrtZero	ABP/PPG cardiac cycle of diastolic phase peak amplitude with respect to zero
5	ABP/PPG_AM_DPE_wrtZero	ABP/PPG cardiac cycle diastolic phase end point amplitude with respect to zero
6	ABP/PPG_NAM_SPO_wrtZero	Normalized ABP/PPG cardiac cycle systolic phase onset amplitude with respect to zero
7	ABP/PPG_NAM_SPP_wrtZero	Normalized ABP/PPG cardiac cycle systolic phase peak amplitude with respect to zero
8	ABP/PPG_NAM_DN_wrtZero	Normalized ABP/PPG cardiac cycle dicrotic notch amplitude with respect to zero
9	ABP/PPG_NAM_DPP_wrtZero	Normalized ABP/PPG cardiac cycle diastolic phase peak amplitude with respect to zero
10	ABP/PPG_NAM_DPE_wrtZero	Normalized ABP/PPG cardiac cycle diastolic phase end point amplitude with respect to zero
11	ABP/PPG_AM_SPP_wrtSPO	ABP/PPG cardiac cycle systolic phase peak amplitude with respect to systolic phase onset
12	ABP/PPG_ AM_ DN_wrtSPO	ABP/PPG cardiac cycle dicrotic notch amplitude with respect to systolic phase onset
13	ABP/PPG_ AM_ DPP_wrtSPO	ABP/PPG cardiac cycle diastolic phase peak amplitude with respect to systolic phase onset
14	ABP/PPG_ AM_ DPE_wrtSPO	ABP/PPG cardiac cycle diastolic phase end point amplitude with respect to systolic phase onset
15	ABP/PPG_ AM_ SPP_wrtDN	ABP/PPG cardiac cycle systolic phase peak amplitude with respect to dicrotic notch
16	ABP/PPG_ AM_ SPP_wrtDPP	ABP/PPG cardiac cycle systolic phase peak amplitude with respect to diastolic phase peak

17	ABP/PPG_ AM_ SPP_wrtDPE	ABP/PPG cardiac cycle systolic phase peak amplitude with respect to diastolic phase end point
18	ABP/PPG_ AM_ DN_wrt DPE	ABP/PPG cardiac cycle dicrotic notch amplitude with respect to diastolic phase end point
19	ABP/PPG_ AM_ DPP_wrtDN	ABP/PPG cardiac cycle diastolic phase peak amplitude with respect to dicrotic notch
20	ABP/PPG_ AM_ DPP_wrtDPE	ABP/PPG cardiac cycle diastolic phase peak amplitude with respect to diastolic phase end point
21	ABP/PPG_NAM_SPP_wrtSPO	Normalized ABP/PPG cardiac cycle systolic phase peak amplitude with respect to systolic phase onset
22	ABP/PPG_NAM_DN_wrtSPO	Normalized ABP/PPG cardiac cycle dicrotic notch amplitude with respect to systolic phase onset
23	ABP/PPG_NAM_DPP_wrtSPO	Normalized ABP/PPG cardiac cycle diastolic phase peak amplitude with respect to systolic phase onset
24	ABP/PPG_NAM_DPE_wrtSPO	Normalized ABP/PPG cardiac cycle diastolic phase end point amplitude with respect to systolic phase onset
25	ABP/PPG_NAM_SPP_wrtDN	Normalized ABP/PPG cardiac cycle systolic phase peak amplitude with respect to dicrotic notch
26	ABP/PPG_NAM_SPP_wrtDPP	Normalized ABP/PPG cardiac cycle systolic phase peak amplitude with respect to diastolic phase peak
27	ABP/PPG_NAM_SPP_wrtDPE	Normalized ABP/PPG cardiac cycle systolic phase peak amplitude with respect to diastolic phase end point
28	ABP/PPG_NAM_DN_wrtDPE	Normalized ABP/PPG cardiac cycle dicrotic notch amplitude with respect to diastolic phase end point
29	ABP/PPG_NAM_DPP_wrtDN	Normalized ABP/PPG cardiac cycle diastolic phase peak amplitude with respect to dicrotic notch
30	ABP/PPG_NAM_DPP_wrtDPE	Normalized ABP/PPG cardiac cycle diastolic phase peak amplitude with respect to diastolic phase end point

2. Amplitude ratio features (31-135)

31	ABP/PPG_AM_RT_SPP_wrtZero_ABP/PPG_SPO_wrtZero	The ratio of the ABP/PPG cardiac cycle systolic phase peak amplitude with respect to zero to the ABP/PPG cardiac cycle systolic phase onset amplitude with respect to zero
32	ABP/PPG_AM_RT_SPP_wrtSPO_ABP/PPG_SPO_wrtZero	The ratio of the ABP/PPG cardiac cycle systolic phase peak amplitude with respect to systolic phase onset to the ABP/PPG cardiac cycle systolic phase onset amplitude with respect to zero
33	ABP/PPG_AM_RT_SPP_wrtDN_ABP/PPG_SPO_wrtZero	The ratio of the ABP/PPG cardiac cycle systolic phase peak amplitude with respect to dicrotic notch to the ABP/PPG cardiac cycle systolic phase onset amplitude with respect to zero
34	ABP/PPG_AM_RT_SPP_wrtDPP_ABP/PPG_SPO_wrtZero	The ratio of the ABP/PPG cardiac cycle systolic phase peak amplitude with respect to diastolic phase peak to the ABP/PPG cardiac cycle systolic phase onset amplitude with respect to zero
35	ABP/PPG_AM_RT_SPP_wrtDPE_ABP/PPG_SPO_wrtZero	The ratio of the ABP/PPG cardiac cycle systolic phase peak amplitude with respect to diastolic phase end point to the ABP/PPG cardiac cycle systolic phase onset amplitude with respect to zero
36	ABP/PPG_AM_RT_DN_wrtZero_ABP/PPG_SPO_wrtZero	The ratio of the ABP/PPG cardiac cycle dicrotic notch amplitude with respect to zero to the ABP/PPG cardiac cycle systolic phase onset amplitude with respect to zero
37	ABP/PPG_AM_RT_DN_wrtSPO_ABP/PPG_SPO_wrtZero	The ratio of the ABP/PPG cardiac cycle dicrotic notch amplitude with respect to systolic phase onset to the ABP/PPG cardiac cycle systolic phase onset amplitude with respect to zero
38	ABP/PPG_AM_RT_DN_wrtDPE_ABP/PPG_SPO_wrtZero	The ratio of the ABP/PPG cardiac cycle dicrotic notch amplitude with respect to diastolic phase end point to the ABP/PPG cardiac cycle systolic phase onset amplitude with respect to zero

39	ABP/PPG_AM_RT_DPP_wrtZero_ABP/PPG_SPO_wrtZero	The ratio of the ABP/PPG cardiac cycle diastolic phase peak amplitude with respect to zero to the ABP/PPG cardiac cycle systolic phase onset amplitude with respect to zero
40	ABP/PPG_AM_RT_DPP_wrtSPO_ABP/PPG_SPO_wrtZero	The ratio of the ABP/PPG cardiac cycle diastolic phase peak amplitude with respect to systolic phase onset to the ABP/PPG cardiac cycle systolic phase onset amplitude with respect to zero
41	ABP/PPG_AM_RT_DPP_wrtDN_ABP/PPG_SPO_wrtZero	The ratio of the ABP/PPG cardiac cycle diastolic phase peak amplitude with respect to dicrotic notch to the ABP/PPG cardiac cycle systolic phase onset amplitude with respect to zero
42	ABP/PPG_AM_RT_DPP_wrtDPE_ABP/PPG_SPO_wrtZero	The ratio of the ABP/PPG cardiac cycle diastolic phase peak amplitude with respect to diastolic phase end point to the ABP/PPG cardiac cycle systolic phase onset amplitude with respect to zero
43	ABP/PPG_AM_RT_DPE_wrtZero_ABP/PPG_SPO_wrtZero	The ratio of the ABP/PPG cardiac cycle diastolic phase end point amplitude with respect to zero to the ABP/PPG cardiac cycle systolic phase onset amplitude with respect to zero
44	ABP/PPG_AM_RT_DPE_wrtSPO_ABP/PPG_SPO_wrtZero	The ratio of the ABP/PPG cardiac cycle diastolic phase end point amplitude with respect to systolic phase onset to the ABP/PPG cardiac cycle systolic phase onset amplitude with respect to zero
45	ABP/PPG_AM_RT_DN_wrtZero_ABP/PPG_SPP_wrtZero	The ratio of the ABP/PPG cardiac cycle dicrotic notch amplitude with respect to zero to the ABP/PPG cardiac cycle systolic phase peak amplitude with respect to zero
46	ABP/PPG_AM_RT_DN_wrtSPO_ABP/PPG_SPP_wrtZero	The ratio of the ABP/PPG cardiac cycle dicrotic notch amplitude with respect to systolic phase onset to the ABP/PPG cardiac cycle systolic phase peak amplitude with respect to zero
47	ABP/PPG_AM_RT_DN_wrtDPE_ABP/PPG_SPP_wrtZero	The ratio of the ABP/PPG cardiac cycle dicrotic notch amplitude with respect to

		diastolic phase end point to the ABP/PPG cardiac cycle systolic phase peak amplitude with respect to zero
48	ABP/PPG_AM_RT_DPP_wrtZero_ABP/PPG_SPP_wrtZero	The ratio of the ABP/PPG cardiac cycle diastolic phase peak amplitude with respect to zero to the ABP/PPG cardiac cycle systolic phase peak amplitude with respect to zero
49	ABP/PPG_AM_RT_DPP_wrtSPO_ABP/PPG_SPP_wrtZero	The ratio of the ABP/PPG cardiac cycle diastolic phase peak amplitude with respect to systolic phase onset to the ABP/PPG cardiac cycle systolic phase peak amplitude with respect to zero
50	ABP/PPG_AM_RT_DPP_wrtDN_ABP/PPG_SPP_wrtZero	The ratio of the ABP/PPG cardiac cycle diastolic phase peak amplitude with respect to dicrotic notch to the ABP/PPG cardiac cycle systolic phase peak amplitude with respect to zero
51	ABP/PPG_AM_RT_DPP_wrtDPE_ABP/PPG_SPP_wrtZero	The ratio of the ABP/PPG cardiac cycle diastolic phase peak amplitude with respect to diastolic phase end point to the ABP/PPG cardiac cycle systolic phase peak amplitude with respect to zero
52	ABP/PPG_AM_RT_DPE_wrtZero_ABP/PPG_SPP_wrtZero	The ratio of the ABP/PPG cardiac cycle diastolic phase end point amplitude with respect to zero to the ABP/PPG cardiac cycle systolic phase peak amplitude with respect to zero
53	ABP/PPG_AM_RT_DPE_wrtSPO_ABP/PPG_SPP_wrtZero	The ratio of the ABP/PPG cardiac cycle diastolic phase end point amplitude with respect to systolic phase onset to the ABP/PPG cardiac cycle systolic phase peak amplitude with respect to zero
54	ABP/PPG_AM_RT_DN_wrtZero_ABP/PPG_SPP_wrtSPO	The ratio of the ABP/PPG cardiac cycle dicrotic notch amplitude with respect to zero to the ABP/PPG cardiac cycle systolic phase peak amplitude with respect to systolic phase onset
55	ABP/PPG_AM_RT_DN_wrtSPO_ABP/PPG_SPP_wrtSPO	The ratio of the ABP/PPG cardiac cycle dicrotic notch amplitude with respect to systolic phase onset to the ABP/PPG cardiac cycle systolic phase peak

		amplitude with respect to systolic phase onset
56	ABP/PPG_AM_RT_DN_wrtDPE_ABP/PPG_SPP_wrtSPO	The ratio of the ABP/PPG cardiac cycle dicrotic notch amplitude with respect to diastolic phase end point to the ABP/PPG cardiac cycle systolic phase peak amplitude with respect to systolic phase onset
57	ABP/PPG_AM_RT_DPP_wrtZero_ABP/PPG_SPP_wrtSPO	The ratio of the ABP/PPG cardiac cycle diastolic phase peak amplitude with respect to zero to the ABP/PPG cardiac cycle systolic phase peak amplitude with respect to systolic phase onset
58	ABP/PPG_AM_RT_DPP_wrtSPO_ABP/PPG_SPP_wrtSPO	The ratio of the ABP/PPG cardiac cycle diastolic phase peak amplitude with respect to systolic phase onset to the ABP/PPG cardiac cycle systolic phase peak amplitude with respect to systolic phase onset
59	ABP/PPG_AM_RT_DPP_wrtDN_ABP/PPG_SPP_wrtSPO	The ratio of the ABP/PPG cardiac cycle diastolic phase peak amplitude with respect to dicrotic notch to the ABP/PPG cardiac cycle systolic phase peak amplitude with respect to systolic phase onset
60	ABP/PPG_AM_RT_DPP_wrtDPE_ABP/PPG_SPP_wrtSPO	The ratio of the ABP/PPG cardiac cycle diastolic phase peak amplitude with respect to diastolic phase end point to the ABP/PPG cardiac cycle systolic phase peak amplitude with respect to systolic phase onset
61	ABP/PPG_AM_RT_DPE_wrtZero_ABP/PPG_SPP_wrtSPO	The ratio of the ABP/PPG cardiac cycle diastolic phase end point amplitude with respect to zero to the ABP/PPG cardiac cycle systolic phase peak amplitude with respect to systolic phase onset
62	ABP/PPG_AM_RT_DPE_wrtSPO_ABP/PPG_SPP_wrtSPO	The ratio of the ABP/PPG cardiac cycle diastolic phase end point amplitude with respect to systolic phase onset to the ABP/PPG cardiac cycle systolic phase peak amplitude with respect to systolic phase onset

63	ABP/PPG_AM_RT_DN_wrtZero_ABP/PPG_SPP_wrtDN	The ratio of the ABP/PPG cardiac cycle dicrotic notch amplitude with respect to zero to the ABP/PPG cardiac cycle systolic phase peak amplitude with respect to dicrotic notch
64	ABP/PPG_AM_RT_DN_wrtSPO_ABP/PPG_SPP_wrtDN	The ratio of the ABP/PPG cardiac cycle dicrotic notch amplitude with respect to systolic phase onset to the ABP/PPG cardiac cycle systolic phase peak amplitude with respect to dicrotic notch
65	ABP/PPG_AM_RT_DN_wrtDPE_ABP/PPG_SPP_wrtDN	The ratio of the ABP/PPG cardiac cycle dicrotic notch amplitude with respect to diastolic phase end point to the ABP/PPG cardiac cycle systolic phase peak amplitude with respect to dicrotic notch
66	ABP/PPG_AM_RT_DPP_wrtZero_ABP/PPG_SPP_wrtDN	The ratio of the ABP/PPG cardiac cycle diastolic phase peak amplitude with respect to zero to the ABP/PPG cardiac cycle systolic phase peak amplitude with respect to dicrotic notch
67	ABP/PPG_AM_RT_DPP_wrtSPO_ABP/PPG_SPP_wrtDN	The ratio of the ABP/PPG cardiac cycle diastolic phase peak amplitude with respect to systolic phase onset to the ABP/PPG cardiac cycle systolic phase peak amplitude with respect to dicrotic notch
68	ABP/PPG_AM_RT_DPP_wrtDN_ABP/PPG_SPP_wrtDN	The ratio of the ABP/PPG cardiac cycle diastolic phase peak amplitude with respect to dicrotic notch to the ABP/PPG cardiac cycle systolic phase peak amplitude with respect to dicrotic notch
69	ABP/PPG_AM_RT_DPP_wrtDPE_ABP/PPG_SPP_wrtDN	The ratio of the ABP/PPG cardiac cycle diastolic phase peak amplitude with respect to diastolic phase end point to the ABP/PPG cardiac cycle systolic phase peak amplitude with respect to dicrotic notch
70	ABP/PPG_AM_RT_DPE_wrtZero_ABP/PPG_SPP_wrtDN	The ratio of the ABP/PPG cardiac cycle diastolic phase end point amplitude with respect to zero to the ABP/PPG cardiac cycle systolic phase peak amplitude with respect to dicrotic notch

71	ABP/PPG_AM_RT_DPE_wrtSPO_ABP/PPG_SPP_wrtDN	The ratio of the ABP/PPG cardiac cycle diastolic phase end point amplitude with respect to systolic phase onset to the ABP/PPG cardiac cycle systolic phase peak amplitude with respect to dicrotic notch
72	ABP/PPG_AM_RT_DN_wrtZero_ABP/PPG_SPP_wrtDPP	The ratio of the ABP/PPG cardiac cycle dicrotic notch amplitude with respect to zero to the ABP/PPG cardiac cycle systolic phase peak amplitude with respect to diastolic phase peak
73	ABP/PPG_AM_RT_DN_wrtSPO_ABP/PPG_SPP_wrtDPP	The ratio of the ABP/PPG cardiac cycle dicrotic notch amplitude with respect to systolic phase onset to the ABP/PPG cardiac cycle systolic phase peak amplitude with respect to diastolic phase peak
74	ABP/PPG_AM_RT_DN_wrtDPE_ABP/PPG_SPP_wrtDPP	The ratio of the ABP/PPG cardiac cycle dicrotic notch amplitude with respect to diastolic phase end point to the ABP/PPG cardiac cycle systolic phase peak amplitude with respect to diastolic phase peak
75	ABP/PPG_AM_RT_DPP_wrtZero_ABP/PPG_SPP_wrtDPP	The ratio of the ABP/PPG cardiac cycle diastolic phase peak amplitude with respect to zero to the ABP/PPG cardiac cycle systolic phase peak amplitude with respect to diastolic phase peak
76	ABP/PPG_AM_RT_DPP_wrtSPO_ABP/PPG_SPP_wrtDPP	The ratio of the ABP/PPG cardiac cycle diastolic phase peak amplitude with respect to systolic phase onset to the ABP/PPG cardiac cycle systolic phase peak amplitude with respect to diastolic phase peak
77	ABP/PPG_AM_RT_DPP_wrtDN_ABP/PPG_SPP_wrtDPP	The ratio of the ABP/PPG cardiac cycle diastolic phase peak amplitude with respect to dicrotic notch to the ABP/PPG cardiac cycle systolic phase peak amplitude with respect to diastolic phase peak
78	ABP/PPG_AM_RT_DPP_wrtDPE_ABP/PPG_SPP_wrtDPP	The ratio of the ABP/PPG cardiac cycle diastolic phase peak amplitude with respect to diastolic phase end point to the ABP/PPG cardiac cycle systolic

		phase peak amplitude with respect to diastolic phase peak
79	ABP/PPG_AM_RT_DPE_wrtZero_ABP/PPG_SPP_wrtDPP	The ratio of the ABP/PPG cardiac cycle diastolic phase end point amplitude with respect to zero to the ABP/PPG cardiac cycle systolic phase peak amplitude with respect to diastolic phase peak
80	ABP/PPG_AM_RT_DPE_wrtSPO_ABP/PPG_SPP_wrtDPP	The ratio of the ABP/PPG cardiac cycle diastolic phase end point amplitude with respect to systolic phase onset to the ABP/PPG cardiac cycle systolic phase peak amplitude with respect to diastolic phase peak
81	ABP/PPG_AM_RT_DN_wrtZero_ABP/PPG_SPP_wrtDPE	The ratio of the ABP/PPG cardiac cycle dicrotic notch amplitude with respect to zero to the ABP/PPG cardiac cycle systolic phase peak amplitude with respect to diastolic phase end point
82	ABP/PPG_AM_RT_DN_wrtSPO_ABP/PPG_SPP_wrtDPE	The ratio of the ABP/PPG cardiac cycle dicrotic notch amplitude with respect to systolic phase onset to the ABP/PPG cardiac cycle systolic phase peak amplitude with respect to diastolic phase end point
83	ABP/PPG_AM_RT_DN_wrtDPE_ABP/PPG_SPP_wrtDPE	The ratio of the ABP/PPG cardiac cycle dicrotic notch amplitude with respect to diastolic phase end point to the ABP/PPG cardiac cycle systolic phase peak amplitude with respect to diastolic phase end point
84	ABP/PPG_AM_RT_DPP_wrtZero_ABP/PPG_SPP_wrtDPE	The ratio of the ABP/PPG cardiac cycle diastolic phase peak amplitude with respect to zero to the ABP/PPG cardiac cycle systolic phase peak amplitude with respect to diastolic phase end point
85	ABP/PPG_AM_RT_DPP_wrtSPO_ABP/PPG_SPP_wrtDPE	The ratio of the ABP/PPG cardiac cycle diastolic phase peak amplitude with respect to systolic phase onset to the ABP/PPG cardiac cycle systolic phase peak amplitude with respect to diastolic phase end point

86	ABP/PPG_AM_RT_DPP_wrtDN_ABP/PPG_SPP_wrtDPE	The ratio of the ABP/PPG cardiac cycle diastolic phase peak amplitude with respect to dicrotic notch to the ABP/PPG cardiac cycle systolic phase peak amplitude with respect to diastolic phase end point
87	ABP/PPG_AM_RT_DPP_wrtDPE_ABP/PPG_SPP_wrtDPE	The ratio of the ABP/PPG cardiac cycle diastolic phase peak amplitude with respect to diastolic phase end point to the ABP/PPG cardiac cycle systolic phase peak amplitude with respect to diastolic phase end point
88	ABP/PPG_AM_RT_DPE_wrtZero_ABP/PPG_SPP_wrtDPE	The ratio of the ABP/PPG cardiac cycle diastolic phase end point amplitude with respect to zero to the ABP/PPG cardiac cycle systolic phase peak amplitude with respect to diastolic phase end point
89	ABP/PPG_AM_RT_DPE_wrtSPO_ABP/PPG_SPP_wrtDPE	The ratio of the ABP/PPG cardiac cycle diastolic phase end point amplitude with respect to systolic phase peak to the ABP/PPG cardiac cycle systolic phase peak amplitude with respect to diastolic phase end point
90	ABP/PPG_AM_RT_DPP_wrtZero_ABP/PPG_DN_wrtZero	The ratio of the ABP/PPG cardiac cycle diastolic phase peak amplitude with respect to zero to the ABP/PPG cardiac cycle dicrotic notch amplitude with respect to zero
91	ABP/PPG_AM_RT_DPP_wrtSPO_ABP/PPG_DN_wrtZero	The ratio of the ABP/PPG cardiac cycle diastolic phase peak amplitude with respect to systolic phase onset to the ABP/PPG cardiac cycle dicrotic notch amplitude with respect to zero
92	ABP/PPG_AM_RT_DPP_wrtDN_ABP/PPG_DN_wrtZero	The ratio of the ABP/PPG cardiac cycle diastolic phase peak amplitude with respect to dicrotic notch to the ABP/PPG cardiac cycle dicrotic notch amplitude with respect to zero
93	ABP/PPG_AM_RT_DPP_wrtDPE_ABP/PPG_DN_wrtZero	The ratio of the ABP/PPG cardiac cycle diastolic phase peak amplitude with respect to diastolic phase end point to the ABP/PPG cardiac cycle dicrotic notch amplitude with respect to zero

94	ABP/PPG_AM_RT_DPE_wrtZero_ABP/PPG_DN_wrtZero	The ratio of the ABP/PPG cardiac cycle diastolic phase end point amplitude with respect to zero to the ABP/PPG cardiac cycle dicrotic notch amplitude with respect to zero
95	ABP/PPG_AM_RT_DPE_wrtSPO_ABP/PPG_DN_wrtZero	The ratio of the ABP/PPG cardiac cycle diastolic phase end point amplitude with respect to systolic phase onset to the ABP/PPG cardiac cycle dicrotic notch amplitude with respect to zero
96	ABP/PPG_AM_RT_DPP_wrtZero_ABP/PPG_DN_wrtSPO	The ratio of the ABP/PPG cardiac cycle diastolic phase peak amplitude with respect to zero to the ABP/PPG cardiac cycle dicrotic notch amplitude with respect to systolic phase onset
97	ABP/PPG_AM_RT_DPP_wrtSPO_ABP/PPG_DN_wrtSPO	The ratio of the ABP/PPG cardiac cycle diastolic phase peak amplitude with respect to systolic phase onset to the ABP/PPG cardiac cycle dicrotic notch amplitude with respect to systolic phase onset
98	ABP/PPG_AM_RT_DPP_wrtDN_ABP/PPG_DN_wrtSPO	The ratio of the ABP/PPG cardiac cycle diastolic phase peak amplitude with respect to dicrotic notch to the ABP/PPG cardiac cycle dicrotic notch amplitude with respect to systolic phase onset
99	ABP/PPG_AM_RT_DPP_wrtDPE_ABP/PPG_DN_wrtSPO	The ratio of the ABP/PPG cardiac cycle diastolic phase peak amplitude with respect to diastolic phase end point to the ABP/PPG cardiac cycle dicrotic notch amplitude with respect to systolic phase onset
100	ABP/PPG_AM_RT_DPE_wrtZero_ABP/PPG_DN_wrtSPO	The ratio of the ABP/PPG cardiac cycle diastolic phase end point amplitude with respect to zero to the ABP/PPG cardiac cycle dicrotic notch amplitude with respect to systolic phase onset
101	ABP/PPG_AM_RT_DPE_wrtSPO_ABP/PPG_DN_wrtSPO	The ratio of the ABP/PPG cardiac cycle diastolic phase end point amplitude with respect to systolic phase onset to the ABP/PPG cardiac cycle dicrotic notch amplitude with respect to systolic phase onset

102	ABP/PPG_AM_RT_DPP_wrtZero_ABP/PPG_DN_wrtDPE	The ratio of the ABP/PPG cardiac cycle diastolic phase peak amplitude with respect to zero to the ABP/PPG cardiac cycle dicrotic notch amplitude with respect to diastolic phase end point
103	ABP/PPG_AM_RT_DPP_wrtSPO_ABP/PPG_DN_wrtDPE	The ratio of the ABP/PPG cardiac cycle diastolic phase peak amplitude with respect to systolic phase onset to the ABP/PPG cardiac cycle dicrotic notch amplitude with respect to diastolic phase end point
104	ABP/PPG_AM_RT_DPP_wrtDN_ABP/PPG_DN_wrtDPE	The ratio of the ABP/PPG cardiac cycle diastolic phase peak amplitude with respect to dicrotic notch to the ABP/PPG cardiac cycle dicrotic notch amplitude with respect to diastolic phase end point
105	ABP/PPG_AM_RT_DPP_wrtDPE_ABP/PPG_DN_wrtDPE	The ratio of the ABP/PPG cardiac cycle diastolic phase peak amplitude with respect to diastolic phase end point to the ABP/PPG cardiac cycle dicrotic notch amplitude with respect to diastolic phase end point
106	ABP/PPG_AM_RT_DPE_wrtZero_ABP/PPG_DN_wrtDPE	The ratio of the ABP/PPG cardiac cycle diastolic phase end point amplitude with respect to zero to the ABP/PPG cardiac cycle dicrotic notch amplitude with respect to diastolic phase end point
107	ABP/PPG_AM_RT_DPE_wrtSPO_ABP/PPG_DN_wrtDPE	The ratio of the ABP/PPG cardiac cycle diastolic phase end point amplitude with respect to systolic phase onset to the ABP/PPG cardiac cycle dicrotic notch amplitude with respect to diastolic phase end point
108	ABP/PPG_AM_RT_DPE_wrtZero_ABP/PPG_DPP_wrtZero	The ratio of the ABP/PPG cardiac cycle diastolic phase end point amplitude with respect to zero to the ABP/PPG cardiac cycle diastolic phase peak amplitude with respect to zero
109	ABP/PPG_AM_RT_DPE_wrtSPO_ABP/PPG_DPP_wrtZero	The ratio of the ABP/PPG cardiac cycle diastolic phase end point amplitude with respect to systolic phase onset to the ABP/PPG cardiac cycle diastolic

		phase peak amplitude with respect to zero
110	ABP/PPG_AM_RT_DPE_wrtZero_ABP/PPG_DPP_wrtSPO	The ratio of the ABP/PPG cardiac cycle diastolic phase end point amplitude with respect to zero to the ABP/PPG cardiac cycle diastolic phase peak amplitude with respect to systolic phase onset
111	ABP/PPG_AM_RT_DPE_wrtSPO_ABP/PPG_DPP_wrtSPO	The ratio of the ABP/PPG cardiac cycle diastolic phase end point amplitude with respect to systolic phase onset to the ABP/PPG cardiac cycle diastolic phase peak amplitude with respect to systolic phase onset
112	ABP/PPG_AM_RT_DPE_wrtZero_ABP/PPG_DPP_wrtDN	The ratio of the ABP/PPG cardiac cycle diastolic phase end point amplitude with respect to zero to the ABP/PPG cardiac cycle diastolic phase peak amplitude with respect to dicrotic notch
113	ABP/PPG_AM_RT_DPE_wrtSPO_ABP/PPG_DPP_wrtDN	The ratio of the ABP/PPG cardiac cycle diastolic phase end point amplitude with respect to systolic phase onset to the ABP/PPG cardiac cycle diastolic phase peak amplitude with respect to dicrotic notch
114	ABP/PPG_AM_RT_DPE_wrtZero_ABP/PPG_DPP_wrtDPE	The ratio of the ABP/PPG cardiac cycle diastolic phase end point amplitude with respect to zero to the ABP/PPG cardiac cycle diastolic phase peak amplitude with respect to diastolic phase end point
115	ABP/PPG_AM_RT_DPE_wrtSPO_ABP/PPG_DPP_wrtDPE	The ratio of the ABP/PPG cardiac cycle diastolic phase end point amplitude with respect to systolic phase onset to the ABP/PPG cardiac cycle diastolic phase peak amplitude with respect to diastolic phase end point
116	ABP/PPG_AM_RT_SPP_wrtSPO_ABP/PPG_SPP_wrtZero	The ratio of the ABP/PPG cardiac cycle systolic phase peak amplitude with respect to systolic phase onset to the ABP/PPG cardiac cycle systolic phase peak amplitude with respect to zero
117	ABP/PPG_AM_RT_SPP_wrtDN_ABP/PPG_SPP_wrtZero	The ratio of the ABP/PPG cardiac cycle systolic phase peak amplitude with

		respect to dicrotic notch to the ABP/PPG cardiac cycle systolic phase peak amplitude with respect to zero
118	ABP/PPG_AM_RT_SPP_wrtDPP_ABP/PPG_SPP_wrtZero	The ratio of the ABP/PPG cardiac cycle systolic phase peak amplitude with respect to diastolic phase peak to the ABP/PPG cardiac cycle systolic phase peak amplitude with respect to zero
119	ABP/PPG_AM_RT_SPP_wrtDPE_ABP/PPG_SPP_wrtZero	The ratio of the ABP/PPG cardiac cycle systolic phase peak amplitude with respect to diastolic phase end point to the ABP/PPG cardiac cycle systolic phase peak amplitude with respect to zero
120	ABP/PPG_AM_RT_SPP_wrtDN_ABP/PPG_SPP_wrtSPO	The ratio of the ABP/PPG cardiac cycle systolic phase peak amplitude with respect to dicrotic notch to the ABP/PPG cardiac cycle systolic phase peak amplitude with respect to systolic phase onset
121	ABP/PPG_AM_RT_SPP_wrtDPP_ABP/PPG_SPP_wrtSPO	The ratio of the ABP/PPG cardiac cycle systolic phase peak amplitude with respect to diastolic phase peak to the ABP/PPG cardiac cycle systolic phase peak amplitude with respect to systolic phase onset
122	ABP/PPG_AM_RT_SPP_wrtDPE_ABP/PPG_SPP_wrtSPO	The ratio of the ABP/PPG cardiac cycle systolic phase peak amplitude with respect to diastolic phase end point to the ABP/PPG cardiac cycle systolic phase peak amplitude with respect to systolic phase onset
123	ABP/PPG_AM_RT_SPP_wrtDPP_ABP/PPG_SPP_wrtDN	The ratio of the ABP/PPG cardiac cycle systolic phase peak amplitude with respect to diastolic phase peak to the ABP/PPG cardiac cycle systolic phase peak amplitude with respect to dicrotic notch
124	ABP/PPG_AM_RT_SPP_wrtDPE_ABP/PPG_SPP_wrtDN	The ratio of the ABP/PPG cardiac cycle systolic phase peak amplitude with respect to diastolic phase end point to the ABP/PPG cardiac cycle systolic phase peak amplitude with respect to dicrotic notch

125	ABP/PPG_AM_RT_SPP_wrtDPE_ABP/PPG_SPP_wrtDPP	The ratio of the ABP/PPG cardiac cycle systolic phase peak amplitude with respect to diastolic phase end point to the ABP/PPG cardiac cycle systolic phase peak amplitude with respect to diastolic phase peak
126	ABP/PPG_AM_RT_DN_wrtSPO_ABP/PPG_DN_wrtZero	The ratio of the ABP/PPG cardiac cycle dicrotic notch amplitude with respect to systolic phase onset to the ABP/PPG dicrotic notch amplitude with respect to zero
127	ABP/PPG_AM_RT_DN_wrtDPE_ABP/PPG_DN_wrtZero	The ratio of the ABP/PPG cardiac cycle dicrotic notch amplitude with respect to diastolic phase end point to the ABP/PPG dicrotic notch amplitude with respect to zero
128	ABP/PPG_AM_RT_DN_wrtDPE_ABP/PPG_DN_wrtSPO	The ratio of the ABP/PPG cardiac cycle dicrotic notch amplitude with respect to diastolic phase end point to the ABP/PPG dicrotic notch amplitude with respect to systolic phase onset
129	ABP/PPG_AM_RT_DPP_wrtSPO_ABP/PPG_DPP_wrtZero	The ratio of the ABP/PPG cardiac cycle diastolic phase peak amplitude with respect to systolic phase onset to the ABP/PPG diastolic phase peak with respect to zero
130	ABP/PPG_AM_RT_DPP_wrtDN_ABP/PPG_DPP_wrtZero	The ratio of the ABP/PPG cardiac cycle diastolic phase peak amplitude with respect to dicrotic notch to the ABP/PPG diastolic phase peak with respect to zero
131	ABP/PPG_AM_RT_DPP_wrtDPE_ABP/PPG_DPP_wrtZero	The ratio of the ABP/PPG cardiac cycle diastolic phase peak amplitude with respect to diastolic phase end point to the ABP/PPG diastolic phase peak with respect to zero
132	ABP/PPG_AM_RT_DPP_wrtDN_ABP/PPG_DPP_wrtSPO	The ratio of the ABP/PPG cardiac cycle diastolic phase peak amplitude with respect to dicrotic notch to the ABP/PPG diastolic phase peak with respect to systolic phase onset
133	ABP/PPG_AM_RT_DPP_wrtDPE_ABP/PPG_DPP_wrtSPO	The ratio of the ABP/PPG cardiac cycle diastolic phase peak amplitude with respect to diastolic phase end point to

		the ABP/PPG diastolic phase peak with respect to systolic phase onset
134	ABP/PPG_AM_RT_DPP_wrtDN_ABP/PPG_DPP_wrtDPE	The ratio of the ABP/PPG cardiac cycle diastolic phase peak amplitude with respect to dicrotic notch to the ABP/PPG diastolic phase peak with respect to diastolic phase end point
135	ABP/PPG_AM_RT_DPE_wrtSPO_ABP/PPG_DPE_wrtZero	The ratio of the ABP/PPG cardiac cycle diastolic phase end point amplitude with respect to systolic phase onset to the ABP/PPG diastolic phase end point with respect to zero

3. Normalized amplitude ratio features (136-240)

136	ABP/PPG_NAM_RT_SPP_wrtZero_ABP/PPG_SPO_wrtZero	The ratio of the normalized ABP/PPG cardiac cycle systolic phase peak amplitude with respect to zero to the normalized ABP/PPG cardiac cycle systolic phase onset amplitude with respect to zero
137	ABP/PPG_NAM_RT_SPP_wrtSPO_ABP/PPG_SPO_wrtZero	The ratio of the normalized ABP/PPG cardiac cycle systolic phase peak amplitude with respect to systolic phase onset to the normalized ABP/PPG cardiac cycle systolic phase onset amplitude with respect to zero
138	ABP/PPG_NAM_RT_SPP_wrtDN_ABP/PPG_SPO_wrtZero	The ratio of the normalized ABP/PPG cardiac cycle systolic phase peak amplitude with respect to dicrotic notch to the normalized ABP/PPG cardiac cycle systolic phase onset amplitude with respect to zero
139	ABP/PPG_NAM_RT_SPP_wrtDPP_ABP/PPG_SPO_wrtZero	The ratio of the normalized ABP/PPG cardiac cycle systolic phase peak amplitude with respect to diastolic phase peak to the normalized ABP/PPG cardiac cycle systolic phase onset amplitude with respect to zero
140	ABP/PPG_NAM_RT_SPP_wrtDPE_ABP/PPG_SPO_wrtZero	The ratio of the normalized ABP/PPG cardiac cycle systolic phase peak amplitude with respect to diastolic phase end point to the normalized

		ABP/PPG cardiac cycle systolic phase onset amplitude with respect to zero
141	ABP/PPG_NAM_RT_DN_wrtZero_ABP/PPG_SPO_wrtZero	The ratio of the normalized ABP/PPG cardiac cycle dicrotic notch with respect to zero to the normalized ABP/PPG cardiac cycle systolic phase onset amplitude with respect to zero
142	ABP/PPG_NAM_RT_DN_wrtSPO_ABP/PPG_SPO_wrtZero	The ratio of the normalized ABP/PPG cardiac cycle dicrotic notch with respect to systolic phase onset to the normalized ABP/PPG cardiac cycle systolic phase onset amplitude with respect to zero
143	ABP/PPG_NAM_RT_DN_wrtDPE_ABP/PPG_SPO_wrtZero	The ratio of the normalized ABP/PPG cardiac cycle dicrotic notch with respect to diastolic phase end point to the normalized ABP/PPG cardiac cycle systolic phase onset amplitude with respect to zero
144	ABP/PPG_NAM_RT_DPP_wrtZero_ABP/PPG_SPO_wrtZero	The ratio of the normalized ABP/PPG cardiac cycle diastolic phase peak with respect to zero to the normalized ABP/PPG cardiac cycle systolic phase onset amplitude with respect to zero
145	ABP/PPG_NAM_RT_DPP_wrtSPO_ABP/PPG_SPOwrtZero	The ratio of the normalized ABP/PPG cardiac cycle diastolic phase peak amplitude with respect to systolic phase onset to the normalized ABP/PPG cardiac cycle systolic phase onset amplitude with respect to zero
146	ABP/PPG_NAM_RT_DPP_wrtDN_ABP/PPG_SPO_wrtZero	The ratio of the normalized ABP/PPG cardiac cycle diastolic phase peak amplitude with respect to dicrotic notch to the normalized ABP/PPG cardiac cycle systolic phase onset amplitude with respect to zero
147	ABP/PPG_NAM_RT_DPP_wrtDPE_ABP/PPG_SPOwrtZero	The ratio of the normalized ABP/PPG cardiac cycle diastolic phase peak with respect to diastolic phase end point to the normalized ABP/PPG cardiac cycle systolic phase onset amplitude with respect to zero

148	ABP/PPG_NAM_RT_DPE_wrtZero_ABP/PPG_SPO_wrtZero	The ratio of the normalized ABP/PPG cardiac cycle diastolic phase end point with respect to zero to the normalized ABP/PPG cardiac cycle systolic phase onset amplitude with respect to zero
149	ABP/PPG_NAM_RT_DPE_wrtSPO_ABP/PPG_SPO_wrtZero	The ratio of the normalized ABP/PPG cardiac cycle diastolic phase end point amplitude with respect to systolic phase onset to the normalized ABP/PPG cardiac cycle systolic phase onset amplitude with respect to zero
150	ABP/PPG_NAM_RT_DN_wrtZero_ABP/PPG_SPP_wrtZero	The ratio of the normalized ABP/PPG cardiac cycle dicrotic notch with respect to zero to the normalized ABP/PPG cardiac cycle systolic phase peak amplitude with respect to zero
151	ABP/PPG_NAM_RT_DN_wrtSPO_ABP/PPG_SPP_wrtZero	The ratio of the normalized ABP/PPG cardiac cycle dicrotic notch with respect to systolic phase onset to the normalized ABP/PPG cardiac cycle systolic phase peak amplitude with respect to zero
152	ABP/PPG_NAM_RT_DN_wrtDPE_ABP/PPG_SPP_wrtZero	The ratio of the normalized ABP/PPG cardiac cycle dicrotic notch with respect to diastolic phase end point to the normalized ABP/PPG cardiac cycle systolic phase peak amplitude with respect to zero
153	ABP/PPG_NAM_RT_DPP_wrtZero_ABP/PPG_SPP_wrtZero	The ratio of the normalized ABP/PPG cardiac cycle diastolic phase peak with respect to zero to the normalized ABP/PPG cardiac cycle systolic phase peak amplitude with respect to zero
154	ABP/PPG_NAM_RT_DPP_wrtSPO_ABP/PPG_SPP_wrtZero	The ratio of the normalized ABP/PPG cardiac cycle diastolic phase peak amplitude with respect to systolic phase onset to the normalized ABP/PPG cardiac cycle systolic phase peak amplitude with respect to zero
155	ABP/PPG_NAM_RT_DPP_wrtDN_ABP/PPG_SPP_wrtZero	The ratio of the normalized ABP/PPG cardiac cycle diastolic phase peak amplitude with respect to dicrotic

		notch to the normalized ABP/PPG cardiac cycle systolic phase peak amplitude with respect to zero
156	ABP/PPG_NAM_RT_DPP_wrtDPE_ABP/PPG_SPP_wrtZero	The ratio of the normalized ABP/PPG cardiac cycle diastolic phase peak with respect to diastolic phase end point to the normalized ABP/PPG cardiac cycle systolic phase peak amplitude with respect to zero
157	ABP/PPG_NAM_RT_DPE_wrtZero_ABP/PPG_SPP_wrtZero	The ratio of the normalized ABP/PPG cardiac cycle diastolic phase end point with respect to zero to the normalized ABP/PPG cardiac cycle systolic phase peak amplitude with respect to zero
158	ABP/PPG_NAM_RT_DPE_wrtSPO_ABP/PPG_SPP_wrtZero	The ratio of the normalized ABP/PPG cardiac cycle diastolic phase end point amplitude with respect to systolic phase onset to the normalized ABP/PPG cardiac cycle systolic phase peak amplitude with respect to zero
159	ABP/PPG_NAM_RT_DN_wrtZero_ABP/PPG_SPP_wrtSPO	The ratio of the normalized ABP/PPG cardiac cycle dicrotic notch with respect to zero to the normalized ABP/PPG cardiac cycle systolic phase peak amplitude with respect to systolic phase onset
160	ABP/PPG_NAM_RT_DN_wrtSPO_ABP/PPG_SPP_wrtSPO	The ratio of the normalized ABP/PPG cardiac cycle dicrotic notch with respect to systolic phase onset to the normalized ABP/PPG cardiac cycle systolic phase peak amplitude with respect to systolic phase onset
161	ABP/PPG_NAM_RT_DN_wrtDPE_ABP/PPG_SPP_wrtSPO	The ratio of the normalized ABP/PPG cardiac cycle dicrotic notch with respect to diastolic phase end point to the normalized ABP/PPG cardiac cycle systolic phase peak amplitude with respect to systolic phase onset
162	ABP/PPG_NAM_RT_DPP_wrtZero_ABP/PPG_SPP_wrtSPO	The ratio of the normalized ABP/PPG cardiac cycle diastolic phase peak with respect to zero to the normalized ABP/PPG cardiac cycle

		systolic phase peak amplitude with respect to systolic phase onset
163	ABP/PPG_NAM_RT_DPP_wrtSPO_ABP/PPG_SPP_wrtSPO	The ratio of the normalized ABP/PPG cardiac cycle diastolic phase peak amplitude with respect to systolic phase onset to the normalized ABP/PPG cardiac cycle systolic phase peak amplitude with respect to systolic phase onset
164	ABP/PPG_NAM_RT_DPP_wrtDN_ABP/PPG_SPP_wrtSPO	The ratio of the normalized ABP/PPG cardiac cycle diastolic phase peak amplitude with respect to dicrotic notch to the normalized ABP/PPG cardiac cycle systolic phase peak amplitude with respect to systolic phase onset
165	ABP/PPG_NAM_RT_DPP_wrtDPE_ABP/PPG_SPP_wrtSPO	The ratio of the normalized ABP/PPG cardiac cycle diastolic phase peak with respect to diastolic phase end point to the normalized ABP/PPG cardiac cycle systolic phase peak amplitude with respect to systolic phase onset
166	ABP/PPG_NAM_RT_DPE_wrtZero_ABP/PPG_SPP_wrtSPO	The ratio of the normalized ABP/PPG cardiac cycle diastolic phase end point with respect to zero to the normalized ABP/PPG cardiac cycle systolic phase peak amplitude with respect to systolic phase onset
167	ABP/PPG_NAM_RT_DPE_wrtSPO_ABP/PPG_SPP_wrtSPO	The ratio of the normalized ABP/PPG cardiac cycle diastolic phase end point amplitude with respect to systolic phase onset to the normalized ABP/PPG cardiac cycle systolic phase peak amplitude with respect to systolic phase onset
168	ABP/PPG_NAM_RT_DN_wrtZero_ABP/PPG_SPP_wrtDN	The ratio of the normalized ABP/PPG cardiac cycle dicrotic notch with respect to zero to the normalized ABP/PPG cardiac cycle systolic phase peak amplitude with respect to dicrotic notch
169	ABP/PPG_NAM_RT_DN_wrtSPO_ABP/PPG_SPP_wrtDN	The ratio of the normalized ABP/PPG cardiac cycle dicrotic notch with

		respect to systolic phase onset to the normalized ABP/PPG cardiac cycle systolic phase peak amplitude with respect to dicrotic notch
170	ABP/PPG_NAM_RT_DN_wrtDPE_ABP/PPG_SPP_wrtDN	The ratio of the normalized ABP/PPG cardiac cycle dicrotic notch with respect to diastolic phase end point to the normalized ABP/PPG cardiac cycle systolic phase peak amplitude with respect to dicrotic notch
171	ABP/PPG_NAM_RT_DPP_wrtZero_ABP/PPG_SPP_wrtDN	The ratio of the normalized ABP/PPG cardiac cycle diastolic phase peak with respect to zero to the normalized ABP/PPG cardiac cycle systolic phase peak amplitude with respect to dicrotic notch
172	ABP/PPG_NAM_RT_DPP_wrtSPO_ABP/PPG_SPP_wrtDN	The ratio of the normalized ABP/PPG cardiac cycle diastolic phase peak amplitude with respect to systolic phase onset to the normalized ABP/PPG cardiac cycle systolic phase peak amplitude with respect to dicrotic notch
173	ABP/PPG_NAM_RT_DPP_wrtDN_ABP/PPG_SPP_wrtDN	The ratio of the normalized ABP/PPG cardiac cycle diastolic phase peak amplitude with respect to dicrotic notch to the normalized ABP/PPG cardiac cycle systolic phase peak amplitude with respect to dicrotic notch
174	ABP/PPG_NAM_RT_DPP_wrtDPE_ABP/PPG_SPP_wrtDN	The ratio of the normalized ABP/PPG cardiac cycle diastolic phase peak with respect to diastolic phase end point to the normalized ABP/PPG cardiac cycle systolic phase peak amplitude with respect to dicrotic notch
175	ABP/PPG_NAM_RT_DPE_wrtZero_ABP/PPG_SPP_wrtDN	The ratio of the normalized ABP/PPG cardiac cycle diastolic phase end point with respect to zero to the normalized ABP/PPG cardiac cycle systolic phase peak amplitude with respect to dicrotic notch

176	ABP/PPG_NAM_RT_DPE_wrtSPO_ABP/PPG_SPP_wrtDN	The ratio of the normalized ABP/PPG cardiac cycle diastolic phase end point amplitude with respect to systolic phase onset to the normalized ABP/PPG cardiac cycle systolic phase peak amplitude with respect to dicrotic notch
177	ABP/PPG_NAM_RT_DN_wrtZero_ABP/PPG_SPP_wrtDPP	The ratio of the normalized ABP/PPG cardiac cycle dicrotic notch with respect to zero to the normalized ABP/PPG cardiac cycle systolic phase peak amplitude with respect to diastolic phase peak
178	ABP/PPG_NAM_RT_DN_wrtSPO_ABP/PPG_SPP_wrtDPP	The ratio of the normalized ABP/PPG cardiac cycle dicrotic notch with respect to systolic phase onset to the normalized ABP/PPG cardiac cycle systolic phase peak amplitude with respect to diastolic phase peak
179	ABP/PPG_NAM_RT_DN_wrtDPE_ABP/PPG_SPP_wrtDPP	The ratio of the normalized ABP/PPG cardiac cycle dicrotic notch with respect to diastolic phase end point to the normalized ABP/PPG cardiac cycle systolic phase peak amplitude with respect to diastolic phase peak
180	ABP/PPG_NAM_RT_DPP_wrtZero_ABP/PPG_SPP_wrtDPP	The ratio of the normalized ABP/PPG cardiac cycle diastolic phase peak with respect to zero to the normalized ABP/PPG cardiac cycle systolic phase peak amplitude with respect to diastolic phase peak
181	ABP/PPG_NAM_RT_DPP_wrtSPO_ABP/PPG_SPP_wrtDPP	The ratio of the normalized ABP/PPG cardiac cycle diastolic phase peak amplitude with respect to systolic phase onset to the normalized ABP/PPG cardiac cycle systolic phase peak amplitude with respect to diastolic phase peak
182	ABP/PPG_NAM_RT_DPP_wrtDN_ABP/PPG_SPP_wrtDPP	The ratio of the normalized ABP/PPG cardiac cycle diastolic phase peak amplitude with respect to dicrotic notch to the normalized ABP/PPG cardiac cycle systolic phase peak

		amplitude with respect to diastolic phase peak
183	ABP/PPG_NAM_RT_DPP_wrtDPE_ABP/PPG_SPP_wrtDPP	The ratio of the normalized ABP/PPG cardiac cycle diastolic phase peak with respect to diastolic phase end point to the normalized ABP/PPG cardiac cycle systolic phase peak amplitude with respect to diastolic phase peak
184	ABP/PPG_NAM_RT_DPE_wrtZero_ABP/PPG_SPP_wrtDPP	The ratio of the normalized ABP/PPG cardiac cycle diastolic phase end point with respect to zero to the normalized ABP/PPG cardiac cycle systolic phase peak amplitude with respect to diastolic phase peak
185	ABP/PPG_NAM_RT_DPE_wrtSPO_ABP/PPG_SPP_wrtDPP	The ratio of the normalized ABP/PPG cardiac cycle diastolic phase end point amplitude with respect to systolic phase onset to the normalized ABP/PPG cardiac cycle systolic phase peak amplitude with respect to diastolic phase peak
186	ABP/PPG_NAM_RT_DN_wrtZero_ABP/PPG_SPP_wrtDPE	The ratio of the normalized ABP/PPG cardiac cycle dicrotic notch with respect to zero to the normalized ABP/PPG cardiac cycle systolic phase peak amplitude with respect to diastolic phase end point
187	ABP/PPG_NAM_RT_DN_wrtSPO_ABP/PPG_SPP_wrtDPE	The ratio of the normalized ABP/PPG cardiac cycle dicrotic notch with respect to systolic phase onset to the normalized ABP/PPG cardiac cycle systolic phase peak amplitude with respect to diastolic phase end point
188	ABP/PPG_NAM_RT_DN_wrtDPE_ABP/PPG_SPP_wrtDPE	The ratio of the normalized ABP/PPG cardiac cycle dicrotic notch with respect to diastolic phase end point to the normalized ABP/PPG cardiac cycle systolic phase peak amplitude with respect to diastolic phase end point
189	ABP/PPG_NAM_RT_DPP_wrtZero_ABP/PPG_SPP_wrtDPE	The ratio of the normalized ABP/PPG cardiac cycle diastolic phase peak with respect to zero to the

		normalized ABP/PPG cardiac cycle systolic phase peak amplitude with respect to diastolic phase end point
190	ABP/PPG_NAM_RT_DPP_wrtSPO_ABP/PPG_SPP_wrtDPE	The ratio of the normalized ABP/PPG cardiac cycle diastolic phase peak amplitude with respect to systolic phase onset to the normalized ABP/PPG cardiac cycle systolic phase peak amplitude with respect to diastolic phase end point
191	ABP/PPG_NAM_RT_DPP_wrtDN_ABP/PPG_SPP_wrtDPE	The ratio of the normalized ABP/PPG cardiac cycle diastolic phase peak amplitude with respect to dicrotic notch to the normalized ABP/PPG cardiac cycle systolic phase peak amplitude with respect to diastolic phase end point
192	ABP/PPG_NAM_RT_DPP_wrtDPE_ABP/PPG_SPP_wrtDPE	The ratio of the normalized ABP/PPG cardiac cycle diastolic phase peak with respect to diastolic phase end point to the normalized ABP/PPG cardiac cycle systolic phase peak amplitude with respect to diastolic phase end point
193	ABP/PPG_NAM_RT_DPE_wrtZero_ABP/PPG_SPP_wrtDPE	The ratio of the normalized ABP/PPG cardiac cycle diastolic phase end point with respect to zero to the normalized ABP/PPG cardiac cycle systolic phase peak amplitude with respect to diastolic phase end point
194	ABP/PPG_NAM_RT_DPE_wrtSPO_ABP/PPG_SPP_wrtDPE	The ratio of the normalized ABP/PPG cardiac cycle diastolic phase end point amplitude with respect to systolic phase onset to the normalized ABP/PPG cardiac cycle systolic phase peak amplitude with respect to diastolic phase end point
195	ABP/PPG_NAM_RT_DPP_wrtZero_ABP/PPG_DN_wrtZero	The ratio of the normalized ABP/PPG cardiac cycle diastolic phase peak amplitude with respect to zero to the normalized ABP/PPG cardiac cycle dicrotic notch amplitude with respect to zero

196	ABP/PPG_NAM_RT_DPP_wrtSPO_ABP/PPG_DN_wrtZero	The ratio of the normalized ABP/PPG cardiac cycle diastolic phase peak amplitude with respect to systolic phase onset to the normalized ABP/PPG cardiac cycle dicrotic notch amplitude with respect to zero
197	ABP/PPG_NAM_RT_DPP_wrtDN_ABP/PPG_DN_wrtZero	The ratio of the normalized ABP/PPG cardiac cycle diastolic phase peak amplitude with respect to dicrotic notch to the normalized ABP/PPG cardiac cycle dicrotic notch amplitude with respect to zero
198	ABP/PPG_NAM_RT_DPP_wrtDPE_ABP/PPG_DN_wrtZero	The ratio of the normalized ABP/PPG cardiac cycle diastolic phase peak amplitude with respect to diastolic phase end point to the normalized ABP/PPG cardiac cycle dicrotic notch amplitude with respect to zero
199	ABP/PPG_NAM_RT_DPE_wrtZero_ABP/PPG_DN_wrtZero	The ratio of the normalized ABP/PPG cardiac cycle diastolic phase end point amplitude with respect to zero to the normalized ABP/PPG cardiac cycle dicrotic notch amplitude with respect to zero
200	ABP/PPG_NAM_RT_DPE_wrtSPO_ABP/PPG_DN_wrtZero	The ratio of the normalized ABP/PPG cardiac cycle diastolic phase end point amplitude with respect to systolic phase onset to the normalized ABP/PPG cardiac cycle dicrotic notch amplitude with respect to zero
201	ABP/PPG_NAM_RT_DPP_wrtZero_ABP/PPG_DN_wrtSPO	The ratio of the normalized ABP/PPG cardiac cycle diastolic phase peak amplitude with respect to zero to the normalized ABP/PPG cardiac cycle dicrotic notch amplitude with respect to systolic phase onset
202	ABP/PPG_NAM_RT_DPP_wrtSPO_ABP/PPG_DN_wrtSPO	The ratio of the normalized ABP/PPG cardiac cycle diastolic phase peak amplitude with respect to systolic phase onset to the normalized ABP/PPG cardiac cycle dicrotic notch amplitude with respect to systolic phase onset

203	ABP/PPG_NAM_RT_DPP_wrtDN_ABP/PPG_DN_wrtSPO	The ratio of the normalized ABP/PPG cardiac cycle diastolic phase peak amplitude with respect to dicrotic notch to the normalized ABP/PPG cardiac cycle dicrotic notch amplitude with respect to systolic phase onset
204	ABP/PPG_NAM_RT_DPP_wrtDPE_ABP/PPG_DN_wrtSPO	The ratio of the normalized ABP/PPG cardiac cycle diastolic phase peak amplitude with respect to diastolic phase end point to the normalized ABP/PPG cardiac cycle dicrotic notch amplitude with respect to systolic phase onset
205	ABP/PPG_NAM_RT_DPE_wrtZero_ABP/PPG_DN_wrtSPO	The ratio of the normalized ABP/PPG cardiac cycle diastolic phase end point amplitude with respect to zero to the normalized ABP/PPG cardiac cycle dicrotic notch amplitude with respect to systolic phase onset
206	ABP/PPG_NAM_RT_DPE_wrtSPO_ABP/PPG_DN_wrtSPO	The ratio of the normalized ABP/PPG cardiac cycle diastolic phase end point amplitude with respect to systolic phase onset to the normalized ABP/PPG cardiac cycle dicrotic notch amplitude with respect to systolic phase onset
207	ABP/PPG_NAM_RT_DPP_wrtZero_ABP/PPG_DN_wrtDPE	The ratio of the normalized ABP/PPG cardiac cycle diastolic phase peak amplitude with respect to zero to the normalized ABP/PPG cardiac cycle dicrotic notch amplitude with respect to diastolic phase end point
208	ABP/PPG_NAM_RT_DPP_wrtSPO_ABP/PPG_DN_wrtDPE	The ratio of the normalized ABP/PPG cardiac cycle diastolic phase peak amplitude with respect to systolic phase onset to the normalized ABP/PPG cardiac cycle dicrotic notch amplitude with respect to diastolic phase end point
209	ABP/PPG_NAM_RT_DPP_wrtDN_ABP/PPG_DN_wrtDPE	The ratio of the normalized ABP/PPG cardiac cycle diastolic phase peak amplitude with respect to dicrotic notch to the normalized ABP/PPG cardiac cycle dicrotic notch amplitude

		with respect to diastolic phase end point
210	ABP/PPG_NAM_RT_DPP_wrtDPE_ABP/PPG_DN_wrtDPE	The ratio of the normalized ABP/PPG cardiac cycle diastolic phase peak amplitude with respect to diastolic phase end point to the normalized ABP/PPG cardiac cycle dicrotic notch amplitude with respect to diastolic phase end point
211	ABP/PPG_NAM_RT_DPE_wrtZero_ABP/PPG_DN_wrtDPE	The ratio of the normalized ABP/PPG cardiac cycle diastolic phase end point amplitude with respect to zero to the normalized ABP/PPG cardiac cycle dicrotic notch amplitude with respect to diastolic phase end point
212	ABP/PPG_NAM_RT_DPE_wrtSPO_ABP/PPG_DN_wrtDPE	The ratio of the normalized ABP/PPG cardiac cycle diastolic phase end point amplitude with respect to systolic phase onset to the normalized ABP/PPG cardiac cycle dicrotic notch amplitude with respect to diastolic phase end point
213	ABP/PPG_NAM_RT_DPE_wrtZero_ABP/PPG_DPP_wrtZero	The ratio of the normalized ABP/PPG cardiac cycle diastolic phase end point amplitude with respect to zero to the normalized ABP/PPG cardiac cycle diastolic phase peak amplitude with respect to zero
214	ABP/PPG_NAM_RT_DPE_wrtSPO_ABP/PPG_DPP_wrtZero	The ratio of the normalized ABP/PPG cardiac cycle diastolic phase end point amplitude with respect to systolic phase onset to the normalized ABP/PPG cardiac cycle diastolic phase peak amplitude with respect to zero
215	ABP/PPG_NAM_RT_DPE_wrtZero_ABP/PPG_DPP_wrtSPO	The ratio of the normalized ABP/PPG cardiac cycle diastolic phase end point amplitude with respect to zero to the normalized ABP/PPG cardiac cycle diastolic phase peak amplitude with respect to systolic phase onset
216	ABP/PPG_NAM_RT_DPE_wrtSPO_ABP/PPG_DPP_wrtSPO	The ratio of the normalized ABP/PPG cardiac cycle diastolic phase end point amplitude with respect to systolic phase onset to the normalized

		ABP/PPG cardiac cycle diastolic phase peak amplitude with respect to systolic phase onset
217	ABP/PPG_NAM_RT_DPE_wrtZero_ABP/PPG_DPP_wrtDN	The ratio of the normalized ABP/PPG cardiac cycle diastolic phase end point amplitude with respect to zero to the normalized ABP/PPG cardiac cycle diastolic phase peak amplitude with respect to dicrotic notch
218	ABP/PPG_NAM_RT_DPE_wrtSPO_ABP/PPG_DPP_wrtDN	The ratio of the normalized ABP/PPG cardiac cycle diastolic phase end point amplitude with respect to systolic phase onset to the normalized ABP/PPG cardiac cycle diastolic phase peak amplitude with respect to dicrotic notch
219	ABP/PPG_NAM_RT_DPE_wrtZero_ABP/PPG_DPP_wrtDPE	The ratio of the normalized ABP/PPG cardiac cycle diastolic phase end point amplitude with respect to zero to the normalized ABP/PPG cardiac cycle diastolic phase peak amplitude with respect to diastolic phase end point
220	ABP/PPG_NAM_RT_DPE_wrtSPO_ABP/PPG_DPP_wrtDPE	The ratio of the normalized ABP/PPG cardiac cycle diastolic phase end point amplitude with respect to systolic phase onset to the normalized ABP/PPG cardiac cycle diastolic phase peak amplitude with respect to diastolic phase end point
221	ABP/PPG_NAM_RT_SPP_wrtSPO_ABP/PPG_SPP_wrtZero	The ratio of the normalized ABP/PPG cardiac cycle systolic phase peak amplitude with respect to systolic phase onset to the normalized ABP/PPG cardiac cycle systolic phase peak amplitude with respect to zero
222	ABP/PPG_NAM_RT_SPP_wrtDN_ABP/PPG_SPP_wrtZero	The ratio of the normalized ABP/PPG cardiac cycle systolic phase peak amplitude with respect to dicrotic notch to the normalized ABP/PPG cardiac cycle systolic phase peak amplitude with respect to zero
223	ABP/PPG_NAM_RT_SPP_wrtDPP_ABP/PPG_SPP_wrtZero	The ratio of the normalized ABP/PPG cardiac cycle systolic phase peak amplitude with respect to diastolic

		phase peak to the normalized ABP/PPG cardiac cycle systolic phase peak amplitude with respect to zero
224	ABP/PPG_NAM_RT_SPP_wrtDPE_ABP/PPG_SPP_wrtZero	The ratio of the normalized ABP/PPG cardiac cycle systolic phase peak amplitude with respect to diastolic phase end point to the normalized ABP/PPG cardiac cycle systolic phase peak amplitude with respect to zero
225	ABP/PPG_NAM_RT_SPP_wrtDN_ABP/PPG_SPP_wrtSPO	The ratio of the normalized ABP/PPG cardiac cycle systolic phase peak amplitude with respect to dicrotic notch to the normalized ABP/PPG cardiac cycle systolic phase peak amplitude with respect to systolic phase onset
226	ABP/PPG_NAM_RT_SPP_wrtDPP_ABP/PPG_SPP_wrtSPO	The ratio of the normalized ABP/PPG cardiac cycle systolic phase peak amplitude with respect to diastolic phase peak to the normalized ABP/PPG cardiac cycle systolic phase peak amplitude with respect to systolic phase onset
227	ABP/PPG_NAM_RT_SPP_wrtDPE_ABP/PPG_SPP_wrtSPO	The ratio of the normalized ABP/PPG cardiac cycle systolic phase peak amplitude with respect to diastolic phase end point to the normalized ABP/PPG cardiac cycle systolic phase peak amplitude with respect to systolic phase onset
228	ABP/PPG_NAM_RT_SPP_wrtDPP_ABP/PPG_SPP_wrtDN	The ratio of the normalized ABP/PPG cardiac cycle systolic phase peak amplitude with respect to diastolic phase peak to the normalized ABP/PPG cardiac cycle systolic phase peak amplitude with respect to systolic dicrotic notch
229	ABP/PPG_NAM_RT_SPP_wrtDPE_ABP/PPG_SPP_wrtDN	The ratio of the normalized ABP/PPG cardiac cycle systolic phase peak amplitude with respect to diastolic phase end point to the normalized ABP/PPG cardiac cycle systolic phase peak amplitude with respect to dicrotic notch

230	ABP/PPG_NAM_RT_SPP_wrtDPE_ABP/PPG_SPP_wrtDPP	The ratio of the normalized ABP/PPG cardiac cycle systolic phase peak amplitude with respect to diastolic phase end point to the normalized ABP/PPG cardiac cycle systolic phase peak amplitude with respect to diastolic phase peak
231	ABP/PPG_NAM_RT_DN_wrtSPO_ABP/PPG_DN_wrtZero	The ratio of the normalized ABP/PPG cardiac cycle dicrotic notch amplitude with respect to systolic phase onset to the normalized ABP/PPG cardiac cycle dicrotic notch amplitude with respect to zero
232	ABP/PPG_NAM_RT_DN_wrtDPE_ABP/PPG_DN_wrtZero	The ratio of the normalized ABP/PPG cardiac cycle dicrotic notch amplitude with respect to diastolic phase end point to the normalized ABP/PPG cardiac cycle dicrotic notch amplitude with respect to zero
233	ABP/PPG_NAM_RT_DN_wrtDPE_ABP/PPG_DN_wrtSPO	The ratio of the normalized ABP/PPG cardiac cycle dicrotic notch amplitude with respect to diastolic phase end point to the normalized ABP/PPG cardiac cycle dicrotic notch amplitude with respect to systolic phase onset
234	ABP/PPG_NAM_RT_DPP_wrtSPO_ABP/PPG_DPP_wrtZero	The ratio of the normalized ABP/PPG cardiac cycle diastolic phase peak amplitude with respect to systolic phase onset to the normalized ABP/PPG cardiac cycle diastolic phase peak amplitude with respect to zero
235	ABP/PPG_NAM_RT_DPP_wrtDN_ABP/PPG_DPP_wrtZero	The ratio of the normalized ABP/PPG cardiac cycle diastolic phase peak amplitude with respect to dicrotic notch to the normalized ABP/PPG cardiac cycle diastolic phase peak amplitude with respect to zero
236	ABP/PPG_NAM_RT_DPP_wrtDPE_ABP/PPG_DPP_wrtZero	The ratio of the normalized ABP/PPG cardiac cycle diastolic phase peak amplitude with respect to diastolic phase end point to the normalized ABP/PPG cardiac cycle diastolic phase peak amplitude with respect to zero

237	ABP/PPG_NAM_RT_DPP_wrtDN_ABP/PPG_DPP_wrtSPO	The ratio of the normalized ABP/PPG cardiac cycle diastolic phase peak amplitude with respect to dicrotic notch to the normalized ABP/PPG cardiac cycle diastolic phase peak amplitude with respect to systolic phase onset
238	ABP/PPG_NAM_RT_DPP_wrtDPE_ABP/PPG_DPP_wrtSPO	The ratio of the normalized ABP/PPG cardiac cycle diastolic phase peak amplitude with respect to diastolic phase end point to the normalized ABP/PPG cardiac cycle diastolic phase peak amplitude with respect to systolic phase onset
239	ABP/PPG_NAM_RT_DPP_wrtDN_ABP/PPG_DPP_wrtDPE	The ratio of the normalized ABP/PPG cardiac cycle diastolic phase peak amplitude with respect to dicrotic notch to the normalized ABP/PPG cardiac cycle diastolic phase peak amplitude with respect to diastolic phase end point
240	ABP/PPG_NAM_RT_DPE_wrtSPO_ABP/PPG_DPE_wrtZero	The ratio of the normalized ABP/PPG cardiac cycle diastolic phase end point amplitude with respect to systolic phase onset to the normalized ABP/PPG cardiac cycle diastolic phase end point amplitude with respect to zero

4. Duration features (241-250)

241	ABP/PPG_D_SPO_wrtSPP	Duration of the ABP/PPG cardiac cycle systolic phase onset with respect to systolic phase peak
242	ABP/PPG_D_SPO_wrtDN	Duration of the ABP/PPG cardiac cycle systolic phase onset with respect to dicrotic notch
243	ABP/PPG_D_SPO_wrtDPP	Duration of the ABP/PPG cardiac cycle systolic phase onset with respect to diastolic phase peak
244	ABP/PPG_D_SPO_wrtDPE	Duration of the ABP/PPG cardiac cycle systolic phase onset with respect to diastolic phase end point
245	ABP/PPG_D_SPP_wrtDN	Duration of the ABP/PPG cardiac cycle systolic phase peak with respect to dicrotic notch
246	ABP/PPG_D_SPP_wrtDPP	Duration of the ABP/PPG cardiac cycle systolic phase peak with respect to diastolic phase peak

247	ABP/PPG_D_SPP_wrtDPE	Duration of the ABP/PPG cardiac cycle systolic phase peak with respect to diastolic phase end point
248	ABP/PPG_D_DN_wrtDPP	Duration of the ABP/PPG cardiac cycle dicrotic notch with respect to diastolic phase peak
249	ABP/PPG_D_DN_wrtDPE	Duration of the ABP/PPG cardiac cycle dicrotic notch with respect to diastolic phase end point
250	ABP/PPG_D_DPP_wrtDPE	Duration of the ABP/PPG cardiac cycle diastolic phase peak with respect to diastolic phase end point

5. Duration ratio features (251-296)

251	ABP/PPG_D_RT_SPP_wrtDN_ABP/PPG_SPO_ wrtSPP	The ratio of the ABP/PPG cardiac cycle systolic phase peak duration with respect to dicrotic notch to the ABP/PPG systolic phase onset duration with respect to systolic phase peak
252	ABP/PPG_D_RT_SPP_wrtDPP_ABP/PPG_SPO_ wrtSPP	The ratio of the ABP/PPG cardiac cycle systolic phase peak duration with respect to diastolic phase peak to the ABP/PPG cardiac cycle systolic phase onset duration with respect to systolic phase peak
253	ABP/PPG_D_RT_SPP_wrtDPE_ABP/PPG_SPO_ wrtSPP	The ratio of the ABP/PPG cardiac cycle systolic phase peak duration with respect to diastolic phase end point to the ABP/PPG cardiac cycle systolic phase onset duration with respect to systolic phase peak
254	ABP/PPG_D_RT_DN_wrtDPP_ABP/PPG_SPO_ wrtSPP	The ratio of the ABP/PPG cardiac cycle dicrotic notch duration with respect to diastolic phase peak to the ABP/PPG cardiac cycle systolic phase onset duration with respect to systolic phase peak
255	ABP/PPG_D_RT_DN_wrtDPE_ABP/PPG_SPO_ wrtSPP	The ratio of the ABP/PPG cardiac cycle dicrotic notch duration with respect to diastolic phase end point to the ABP/PPG cardiac cycle systolic phase onset duration with respect to systolic phase peak
256	ABP/PPG_D_RT_DPP_wrtDPE_ABP/PPG_SPO_ wrtSPP	The ratio of the ABP/PPG cardiac cycle diastolic phase peak duration with respect to diastolic phase end point to the ABP/PPG cardiac cycle systolic phase onset duration with respect to systolic phase peak
257	ABP/PPG_D_RT_SPP_wrtDN_ABP/PPG_SPO_ wrtDN	The ratio of the ABP/PPG cardiac cycle systolic phase peak duration with respect to dicrotic notch to the ABP/PPG cardiac cycle systolic

		phase onset duration with respect to dicrotic notch
258	ABP/PPG_D_RT_SPP_wrtDPP_ABP/PPG_SPO_wrtDN	The ratio of the ABP/PPG cardiac cycle systolic phase peak duration with respect to diastolic phase peak to the ABP/PPG cardiac cycle systolic phase onset duration with respect to dicrotic notch
259	ABP/PPG_D_RT_SPP_wrtDPE_ABP/PPG_SPO_wrtDN	The ratio of the ABP/PPG cardiac cycle systolic phase peak duration with respect to diastolic phase end point to the ABP/PPG cardiac cycle systolic phase onset duration with respect to dicrotic notch
260	ABP/PPG_D_RT_DN_wrtDPP_ABP/PPG_SPO_wrtDN	The ratio of the ABP/PPG cardiac cycle dicrotic notch duration with respect to diastolic phase peak to the ABP/PPG cardiac cycle systolic phase onset duration with respect to dicrotic notch
261	ABP/PPG_D_RT_DN_wrtDPE_ABP/PPG_SPO_wrtDN	The ratio of the ABP/PPG cardiac cycle dicrotic notch duration with respect to diastolic phase end point to the ABP/PPG cardiac cycle systolic phase onset duration with respect to dicrotic notch
262	ABP/PPG_D_RT_DPP_wrtDPE_ABP/PPG_SPO_wrtDN	The ratio of the ABP/PPG cardiac cycle diastolic phase peak duration with respect to diastolic phase end point to the ABP/PPG cardiac cycle systolic phase onset duration with respect to dicrotic notch
263	ABP/PPG_D_RT_SPP_wrtDN_ABP/PPG_SPO_wrtDN	The ratio of the ABP/PPG cardiac cycle systolic phase peak duration with respect to dicrotic notch to the ABP/PPG cardiac cycle systolic phase onset duration with respect to dicrotic notch
264	ABP/PPG_D_RT_SPP_wrtDPP_ABP/PPG_SPO_wrtDN	The ratio of the ABP/PPG cardiac cycle systolic phase peak duration with respect to diastolic phase peak to the ABP/PPG cardiac cycle systolic phase onset duration with respect to dicrotic notch
265	ABP/PPG_D_RT_SPP_wrtDPE_ABP/PPG_SPO_wrtDN	The ratio of the ABP/PPG cardiac cycle systolic phase peak duration with respect to diastolic phase end point to the ABP/PPG cardiac cycle systolic phase onset duration with respect to dicrotic notch
266	ABP/PPG_D_RT_DN_wrtDPP_ABP/PPG_SPO_wrtDN	The ratio of the ABP/PPG cardiac cycle dicrotic notch duration with respect to diastolic phase

		peak to the ABP/PPG cardiac cycle systolic phase onset duration with respect to dicrotic notch
267	ABP/PPG_D_RT_DN_wrtDPE_ABP/PPG_SPO_wrtDN	The ratio of the ABP/PPG cardiac cycle dicrotic notch duration with respect to diastolic phase end point to the ABP/PPG cardiac cycle systolic phase onset duration with respect to dicrotic notch
268	ABP/PPG_D_RT_DPP_wrtDPE_ABP/PPG_SPO_wrtDPP	The ratio of the ABP/PPG cardiac cycle diastolic phase peak duration with respect to diastolic phase end point to the ABP/PPG cardiac cycle systolic phase onset duration with respect to diastolic phase peak
269	ABP/PPG_D_RT_SPP_wrtDN_ABP/PPG_SPO_wrtDPE	The ratio of the ABP/PPG cardiac cycle systolic phase peak duration with respect to dicrotic notch to the ABP/PPG cardiac cycle systolic phase onset duration with respect to diastolic phase end point
270	ABP/PPG_D_RT_SPP_wrtDPP_ABP/PPG_SPO_wrtDPE	The ratio of the ABP/PPG cardiac cycle systolic phase peak duration with respect to diastolic phase peak to the ABP/PPG cardiac cycle systolic phase onset duration with respect to diastolic phase end point
271	ABP/PPG_D_RT_SPP_wrtDPE_ABP/PPG_SPO_wrtDPE	The ratio of the ABP/PPG cardiac cycle systolic phase peak duration with respect to diastolic phase end point to the ABP/PPG cardiac cycle systolic phase onset duration with respect to diastolic phase end point
272	ABP/PPG_D_RT_DN_wrtDPP_ABP/PPG_SPO_wrtDPE	The ratio of the ABP/PPG cardiac cycle dicrotic notch duration with respect to diastolic phase peak to the ABP/PPG cardiac cycle systolic phase onset duration with respect to diastolic phase end point
273	ABP/PPG_D_RT_DN_wrtDPE_ABP/PPG_SPO_wrtDPE	The ratio of the ABP/PPG cardiac cycle dicrotic notch duration with respect to diastolic phase end point to the ABP/PPG cardiac cycle systolic phase onset duration with respect to diastolic phase end point
274	ABP/PPG_D_RT_DPP_wrtDPE_ABP/PPG_SPO_wrtDPE	The ratio of the ABP/PPG cardiac cycle diastolic phase peak duration with respect to diastolic phase end point to the ABP/PPG cardiac cycle systolic phase onset duration with respect to diastolic phase end point

275	ABP/PPG_D_RT_DN_wrtDPP_ABP/PPG_SPP_wrtDN	The ratio of the ABP/PPG cardiac cycle dicrotic notch duration with respect to diastolic phase peak to the ABP/PPG cardiac cycle systolic phase peak duration with respect to dicrotic notch
276	ABP/PPG_D_RT_DN_wrtDPE_ABP/PPG_SPP_wrtDN	The ratio of the ABP/PPG cardiac cycle dicrotic notch duration with respect to diastolic phase end point to the ABP/PPG cardiac cycle systolic phase peak duration with respect to dicrotic notch
277	ABP/PPG_D_RT_DPP_wrtDPE_ABP/PPG_SPP_wrtDN	The ratio of the ABP/PPG cardiac cycle diastolic phase peak duration with respect to diastolic phase end point to the ABP/PPG cardiac cycle systolic phase peak duration with respect to dicrotic notch
278	ABP/PPG_D_RT_DN_wrtDPP_ABP/PPG_SPP_wrtDPP	The ratio of the ABP/PPG cardiac cycle dicrotic notch duration with respect to diastolic phase peak to the ABP/PPG cardiac cycle systolic phase peak duration with respect to diastolic phase peak
279	ABP/PPG_D_RT_DN_wrtDPE_ABP/PPG_SPP_wrtDPP	The ratio of the ABP/PPG cardiac cycle dicrotic notch duration with respect to diastolic phase end point to the ABP/PPG cardiac cycle systolic phase peak duration with respect to diastolic phase peak
280	ABP/PPG_D_RT_DPP_wrtDPE_ABP/PPG_SPP_wrtDPP	The ratio of the ABP/PPG cardiac cycle diastolic phase peak duration with respect to diastolic phase end point to the ABP/PPG cardiac cycle systolic phase peak duration with respect to diastolic phase peak
281	ABP/PPG_D_RT_DN_wrtDPP_ABP/PPG_SPP_wrtDPE	The ratio of the ABP/PPG cardiac cycle dicrotic notch duration with respect to diastolic phase peak to the ABP/PPG cardiac cycle systolic phase peak duration with respect to diastolic phase end point
282	ABP/PPG_D_RT_DN_wrtDPE_ABP/PPG_SPP_wrtDPE	The ratio of the ABP/PPG cardiac cycle dicrotic notch duration with respect to diastolic phase end point to the ABP/PPG cardiac cycle systolic phase peak duration with respect to diastolic phase end point
283	ABP/PPG_D_RT_DPP_wrtDPE_ABP/PPG_SPP_wrtDPE	The ratio of the ABP/PPG cardiac cycle diastolic phase peak duration with respect to diastolic phase end point to the ABP/PPG cardiac cycle

		systolic phase peak duration with respect to diastolic phase end point
284	ABP/PPG_D_RT_DPP_wrtDPE_ABP/PPG_DN_wrtDPP	The ratio of the ABP/PPG cardiac cycle diastolic phase peak duration with respect to diastolic phase end point to the ABP/PPG cardiac cycle dicrotic notch duration with respect to diastolic phase peak
285	ABP/PPG_D_RT_DPP_wrtDPE_ABP/PPG_DN_wrtDPE	The ratio of the ABP/PPG cardiac cycle diastolic phase peak duration with respect to diastolic phase end point to the ABP/PPG cardiac cycle dicrotic notch duration with respect to diastolic phase end point
286	ABP/PPG_D_RT_SPO_wrtDN_ABP/PPG_SPO_wrtSPP	The ratio of the ABP/PPG cardiac cycle systolic phase onset duration with respect to dicrotic notch to the ABP/PPG cardiac cycle systolic phase onset duration with respect to systolic phase peak
287	ABP/PPG_D_RT_SPO_wrtDPP_ABP/PPG_SPO_wrtSPP	The ratio of the ABP/PPG cardiac cycle systolic phase onset duration with respect to diastolic phase peak to the ABP/PPG cardiac cycle systolic phase onset duration with respect to systolic phase peak
288	ABP/PPG_D_RT_SPO_wrtDPE_ABP/PPG_SPO_wrtSPP	The ratio of the ABP/PPG cardiac cycle systolic phase onset duration with respect to diastolic phase end point to the ABP/PPG cardiac cycle systolic phase onset duration with respect to systolic phase peak
289	ABP/PPG_D_RT_SPO_wrtDPP_ABP/PPG_SPO_wrtDN	The ratio of the ABP/PPG cardiac cycle systolic phase onset duration with respect to diastolic phase peak to the ABP/PPG cardiac cycle systolic phase onset duration with respect to dicrotic notch
290	ABP/PPG_D_RT_SPO_wrtDPE_ABP/PPG_SPO_wrtDN	The ratio of the ABP/PPG cardiac cycle systolic phase onset duration with respect to diastolic phase end point to the ABP/PPG cardiac cycle systolic phase onset duration with respect to dicrotic notch
291	ABP/PPG_D_RT_SPO_wrtDPE_ABP/PPG_SPO_wrtDPP	The ratio of the ABP/PPG cardiac cycle systolic phase onset duration with respect to diastolic phase end point to the ABP/PPG cardiac cycle systolic phase onset duration with respect to diastolic phase peak
292	ABP/PPG_D_RT_SPP_wrtDPP_ABP/PPG_SPP_wrtDN	The ratio of the ABP/PPG cardiac cycle systolic phase peak duration with respect to diastolic

		phase peak to the ABP/PPG cardiac cycle systolic phase peak duration with respect to dicrotic notch
293	ABP/PPG_D_RT_SPP_wrtDPE_ABP/PPG_SPP_wrtDN	The ratio of the ABP/PPG cardiac cycle systolic phase peak duration with respect to diastolic phase end point to the ABP/PPG cardiac cycle systolic phase peak duration with respect to dicrotic notch
294	ABP/PPG_D_RT_SPP_wrtDPE_ABP/PPG_SPP_wrtDPP	The ratio of the ABP/PPG cardiac cycle systolic phase peak duration with respect to diastolic phase end point to the ABP/PPG cardiac cycle systolic phase peak duration with respect to diastolic phase peak
295	ABP/PPG_D_RT_DN_wrtDPE_ABP/PPG_DN_wrtDPP	The ratio of the ABP/PPG cardiac cycle dicrotic notch duration with respect to diastolic phase end point to the ABP/PPG cardiac cycle dicrotic notch duration with respect to diastolic phase peak
296	ABP/PPG_HR	The ratio of the ABP/PPG cardiac cycle

6. Average features (297-306)

297	ABP/PPG_D_Avg_SPO_wrtSPP	Average duration of the ABP/PPG cardiac cycle systolic phase onset with respect to systolic phase peak
298	ABP/PPG_D_Avg_SPO_wrtDN	Average duration of the ABP/PPG cardiac cycle systolic phase onset with respect to dicrotic notch
299	ABP/PPG_D_Avg_SPO_wrtDPP	Average duration of the ABP/PPG cardiac cycle systolic phase onset with respect to diastolic phase peak
300	ABP/PPG_D_Avg_SPO_wrtDPE	Average duration of the ABP/PPG cardiac cycle systolic phase onset with respect to diastolic phase end point
301	ABP/PPG_D_Avg_SPP_wrtDN	Average duration of the ABP/PPG cardiac cycle systolic phase peak with respect to dicrotic notch
302	ABP/PPG_D_Avg_SPP_wrtDPP	Average duration of the ABP/PPG cardiac cycle systolic phase peak with respect to diastolic phase peak
303	ABP/PPG_D_Avg_SPP_wrtDPE	Average duration of the ABP/PPG cardiac cycle systolic phase peak with respect to diastolic phase end point
304	ABP/PPG_D_Avg_DN_wrtDPP	Average duration of the ABP/PPG cardiac cycle dicrotic notch with respect to diastolic phase peak

305	ABP/PPG_D_Avg_DN_wrtDPE	Average duration of the ABP/PPG cardiac cycle dicrotic notch with respect to diastolic phase end point
306	ABP/PPG_D_Avg_DPP_wrtDPE	Average duration of the ABP/PPG cardiac cycle diastolic phase peak with respect to diastolic phase end point

7. Average features with subtracted diastolic end point (307-316)

307	ABP/PPG_D_Avg_wsDPE_SPO_wrtSPP	Average duration of the ABP/PPG cardiac cycle systolic phase onset with respect to systolic phase peak, with subtracted diastolic phase end point
308	ABP/PPG_D_Avg_wsDPE_SPO_wrtDN	Average duration of the ABP/PPG cardiac cycle systolic phase onset with respect to dicrotic notch, with subtracted diastolic phase end point
309	ABP/PPG_D_Avg_wsDPE_SPO_wrtDPP	Average duration of the ABP/PPG cardiac cycle systolic phase onset with respect to diastolic phase peak, with subtracted diastolic phase end point
310	ABP/PPG_D_Avg_wsDPE_SPO_wrtDPD	Average duration of the ABP/PPG cardiac cycle systolic phase onset with respect to diastolic phase end point, with subtracted diastolic phase end point
311	ABP/PPG_D_Avg_wsDPE_SPP_wrtDN	Average duration of the ABP/PPG cardiac cycle systolic phase peak with respect to dicrotic notch, with subtracted diastolic phase end point
312	ABP/PPG_D_Avg_wsDPE_SPP_wrtDPP	Average duration of the ABP/PPG cardiac cycle systolic phase peak with respect to diastolic phase peak, with subtracted diastolic phase end point
313	ABP/PPG_D_Avg_wsDPE_SPP_wrtDPE	Average duration of the ABP/PPG cardiac cycle systolic phase peak with respect to diastolic phase end point, with subtracted diastolic phase end point
314	ABP/PPG_D_Avg_wsDPE_DN_wrtDPP	Average duration of the ABP/PPG cardiac cycle dicrotic notch with respect to diastolic phase peak, with subtracted diastolic phase end point
315	ABP/PPG_D_Avg_wsDPE_DN_wrtDPE	Average duration of the ABP/PPG cardiac cycle dicrotic notch with respect to diastolic phase end point, with subtracted diastolic phase end point
316	ABP/PPG_D_Avg_wsDPE_DPP_wrtDPE	Average duration of the ABP/PPG cardiac cycle diastolic phase peak with respect to diastolic phase end point, with subtracted diastolic phase end point

8. **Normalized average features (317-326)**

317	ABP/PPG_D_NAvg_SPO_wrtSPP	Average duration of the normalized ABP/PPG cardiac cycle systolic phase onset with respect to systolic phase peak
318	ABP/PPG_D_NAvg_SPO_wrtDN	Average duration of the normalized ABP/PPG cardiac cycle systolic phase onset with respect to dicrotic notch
319	ABP/PPG_D_NAvg_SPO_wrtDPP	Average duration of the normalized ABP/PPG cardiac cycle systolic phase onset with respect to diastolic phase peak
320	ABP/PPG_D_NAvg_SPO_wrtDPE	Average duration of the normalized ABP/PPG cardiac cycle systolic phase onset with respect to diastolic phase end point
321	ABP/PPG_D_NAvg_SPP_wrtDN	Average duration of the normalized ABP/PPG cardiac cycle systolic phase peak with respect to dicrotic notch
322	ABP/PPG_D_NAvg_SPP_wrtDPP	Average duration of the normalized ABP/PPG cardiac cycle systolic phase peak with respect to diastolic phase peak
323	ABP/PPG_D_NAvg_SPP_wrtDPE	Average duration of the normalized ABP/PPG cardiac cycle systolic phase peak with respect to diastolic phase end point
324	ABP/PPG_D_NAvg_DN_wrtDPP	Average duration of the normalized ABP/PPG cardiac cycle dicrotic notch with respect to diastolic phase peak
325	ABP/PPG_D_NAvg_DN_wrtDPE	Average duration of the normalized ABP/PPG cardiac cycle dicrotic notch with respect to diastolic phase end point
326	ABP/PPG_D_NAvg_DPP_wrtDPE	Average duration of the normalized ABP/PPG cardiac cycle diastolic phase peak with respect to diastolic phase end point

9. **Normalized average features with subtracted diastolic end point (327-336)**

327	ABP/PPG_D_NAvg_wsDPE_SPO_wrtSPP	Average duration of the normalized ABP/PPG cardiac cycle systolic phase onset with respect to systolic phase peak, with subtracted diastolic phase end point
328	ABP/PPG_D_NAvg_wsDPE_SPO_wrtDN	Average duration of the normalized ABP/PPG cardiac cycle systolic phase onset with respect to dicrotic notch, with subtracted diastolic phase end point
329	ABP/PPG_D_NAvg_wsDPE_SPO_wrtDPP	Average duration of the normalized ABP/PPG cardiac cycle systolic phase onset with respect to diastolic

		phase peak, with subtracted diastolic phase end point
330	ABP/PPG_D_NAvg_wsDPE_SPO_wrtDPE	Average duration of the normalized ABP/PPG cardiac cycle systolic phase onset with respect to diastolic phase end point, with subtracted diastolic phase end point
331	ABP/PPG_D_NAvg_wsDPE_SPP_wrtDN	Average duration of the normalized ABP/PPG cardiac cycle systolic phase peak with respect to dicrotic notch, with subtracted diastolic phase end point
332	ABP/PPG_D_NAvg_wsDPE_SPP_wrtDPP	Average duration of the normalized ABP/PPG cardiac cycle systolic phase peak with respect to diastolic phase peak, with subtracted diastolic phase end point
333	ABP/PPG_D_NAvg_wsDPE_SPP_wrtDPE	Average duration of the normalized ABP/PPG cardiac cycle systolic phase peak with respect to diastolic phase end point, with subtracted diastolic phase end point
334	ABP/PPG_D_NAvg_wsDPE_DN_wrtDPP	Average duration of the normalized ABP/PPG cardiac cycle dicrotic notch with respect to diastolic phase peak, with subtracted diastolic phase end point
335	ABP/PPG_D_NAvg_wsDPE_DN_wrtDPE	Average duration of the normalized ABP/PPG cardiac cycle dicrotic notch with respect to diastolic phase end point, with subtracted diastolic phase end point
336	ABP/PPG_D_NAvg_wsDPE_DPP_wrtDPE	Average duration of the normalized ABP/PPG cardiac cycle diastolic phase peak with respect to diastolic phase end point, with subtracted diastolic phase end point

10. Median features (337-356)

337	ABP/PPG_D_Med_SPO_wrtSPP	Median duration of the ABP/PPG cardiac cycle systolic phase onset with respect to systolic phase peak
338	ABP/PPG_D_Med_SPO_wrtDN	Median duration of the ABP/PPG cardiac cycle systolic phase onset with respect to dicrotic notch
339	ABP/PPG_D_Med_SPO_wrtDPP	Median duration of the ABP/PPG cardiac cycle systolic phase onset with respect to diastolic phase peak
340	ABP/PPG_D_Med_SPO_wrtDPE	Median duration of the ABP/PPG cardiac cycle systolic phase onset with respect to diastolic phase end point
341	ABP/PPG_D_Med_SPP_wrtDN	Median duration of the ABP/PPG cardiac cycle systolic phase peak with respect to dicrotic notch

342	ABP/PPG_D_Med_SPP_wrtDPP	Median duration of the ABP/PPG cardiac cycle systolic phase peak with respect to diastolic phase peak
343	ABP/PPG_D_Med_SPP_wrtDPE	Median duration of the ABP/PPG cardiac cycle systolic phase peak with respect to diastolic phase end point
344	ABP/PPG_D_Med_DN_wrtDPP	Median duration of the ABP/PPG cardiac cycle dicrotic notch with respect to diastolic phase peak
345	ABP/PPG_D_Med_DN_wrtDPE	Median duration of the ABP/PPG cardiac cycle dicrotic notch with respect to diastolic phase end point
346	ABP/PPG_D_Med_DPP_wrtDPE	Median duration of the ABP/PPG cardiac cycle diastolic phase peak with respect to diastolic phase end point
347	ABP/PPG_D_NMed_SPO_wrtSPP	Median duration of the normalized ABP/PPG cardiac cycle systolic phase onset with respect to systolic phase peak
348	ABP/PPG_D_NMed_SPO_wrtDN	Median duration of the normalized ABP/PPG cardiac cycle systolic phase onset with respect to dicrotic notch
349	ABP/PPG_D_NMed_SPO_wrtDPP	Median duration of the normalized ABP/PPG cardiac cycle systolic phase onset with respect to diastolic phase peak
350	ABP/PPG_D_NMED_SPO_wrtDPE	Median duration of the normalized ABP/PPG cardiac cycle systolic phase onset with respect to diastolic phase end point
351	ABP/PPG_D_NMed_SPP_wrtDN	Median duration of the normalized ABP/PPG cardiac cycle systolic phase peak with respect to dicrotic notch
352	ABP/PPG_D_NMed_SPP_wrtDPP	Median duration of the normalized ABP/PPG cardiac cycle systolic phase peak with respect to diastolic phase peak
353	ABP/PPG_D_NMed_SPP_wrtDPE	Median duration of the normalized ABP/PPG cardiac cycle systolic phase peak with respect to diastolic phase end point
354	ABP/PPG_D_NMed_DN_wrtDPP	Median duration of the normalized ABP/PPG cardiac cycle dicrotic notch with respect to diastolic phase peak
355	ABP/PPG_D_NMed_DN_wrtDPE	Median duration of the normalized ABP/PPG cardiac cycle dicrotic notch with respect to diastolic phase end point

356	ABP/PPG_D_NMed_DPP_wrtDPE	Median duration of the normalized ABP/PPG cardiac cycle diastolic phase peak with respect to diastolic phase end point

11. Root mean square features (357-376)

357	ABP/PPG_D_RMS_SPO_wrtSPP	Root mean square duration of the ABP/PPG cardiac cycle systolic phase onset with respect to systolic phase peak
358	ABP/PPG_D_RMS_SPO_wrtDN	Root mean square duration of the ABP/PPG cardiac cycle systolic phase onset with respect to dicrotic notch
359	ABP/PPG_D_RMS_SPO_wrtDPP	Root mean square duration of the ABP/PPG cardiac cycle systolic phase onset with respect to diastolic phase peak
360	ABP/PPG_D_RMS_SPO_wrtDPE	Root mean square duration of the ABP/PPG cardiac cycle systolic phase onset with respect to diastolic phase end point
361	ABP/PPG_D_RMS_SPP_wrtDN	Root mean square duration of the ABP/PPG cardiac cycle systolic phase peak with respect to dicrotic notch
362	ABP/PPG_D_RMS_ SPP_wrtDPP	Root mean square duration of the ABP/PPG cardiac cycle systolic phase peak with respect to diastolic phase peak
363	ABP/PPG_D_RMS_ SPP_wrtDPE	Root mean square duration of the ABP/PPG cardiac cycle systolic phase peak with respect to diastolic phase end point
364	ABP/PPG_D_RMS_ DN_wrtDPP	Root mean square duration of the ABP/PPG cardiac cycle dicrotic notch with respect to diastolic phase peak
365	ABP/PPG_D_RMS_ DN_wrtDPE	Root mean square duration of the ABP/PPG cardiac cycle dicrotic notch with respect to diastolic phase end point
366	ABP/PPG_D_RMS_ DPP_wrtDPE	Root mean square duration of the ABP/PPG cardiac cycle diastolic phase peak with respect to diastolic phase end point
367	ABP/PPG_D_NRMS_SPO_wrtSPP	Root mean square duration of the normalized ABP/PPG cardiac cycle systolic phase onset with respect to systolic phase peak
368	ABP/PPG_D_NRMS_SPO_wrtDN	Root mean square duration of the normalized ABP/PPG cardiac cycle systolic phase onset with respect to dicrotic notch

369	ABP/PPG_D_NRMS_SPO_wrtDPP	Root mean square duration of the normalized ABP/PPG cardiac cycle systolic phase onset with respect to diastolic phase peak
370	ABP/PPG_D_NRMS_SPO_wrtDPE	Root mean square duration of the normalized ABP/PPG cardiac cycle systolic phase onset with respect to diastolic phase end point
371	ABP/PPG_D_NRMS_SPP_wrtDN	Root mean square duration of the normalized ABP/PPG cardiac cycle systolic phase peak with respect of dicrotic notch
372	ABP/PPG_D_NRMS_ SPP _wrtDPP	Root mean square duration of the normalized ABP/PPG cardiac cycle systolic phase peak with respect to diastolic phase peak
373	ABP/PPG_D_NRMS_ SPP_wrtDPE	Root mean square duration of the normalized ABP/PPG cardiac cycle systolic phase peak with respect to diastolic phase end point
374	ABP/PPG_D_NRMS_ DN_wrtDPP	Root mean square duration of the normalized ABP/PPG cardiac cycle dicrotic notch with respect to diastolic phase peak
375	ABP/PPG_D_NRMS_ DN_wrtDPE	Root mean square duration of the normalized ABP/PPG cardiac cycle dicrotic notch with respect to diastolic phase end point
376	ABP/PPG_D_NRMS_ DPP_wrtDPE	Root mean square duration of the normalized ABP/PPG cardiac cycle diastolic phase peak with respect to diastolic phase end point

12. Percentage features (377-412)

377	ABP/PPG_W_SRP10	ABP/PPG cardiac cycle systolic rise phase width at 10% of the cardiac cycle
378	ABP/PPG_W_ODP10	ABP/PPG cardiac cycle overall decay phase width at 10% of the cardiac cycle
379	ABP/PPG_W_SRP10_ODP10	Sum of the ABP/PPG cardiac cycle systolic rise phase width and overall decay phase width at 10% of the cardiac cycle
380	ABP/PPG_RT_ODP10_W_SRP10_W	The ratio of the ABP/PPG cardiac cycle overall decay phase width at 10% of the cardiac cycle to the ABP/PPG cardiac cycle systolic rise phase width at 10% of the cardiac cycle
381	ABP/PPG_RT_ODP10_W_SRP10_ODP10_W	The ratio of the ABP/PPG cardiac cycle overall decay phase width at 10% of the cardiac cycle to the sum of the ABP/PPG cardiac cycle systolic rise phase width and overall decay phase width at 10% of the cardiac cycle

382	ABP/PPG_RT_SRP10_W_SRP10_ODP10_W	The ratio of the ABP/PPG cardiac cycle systolic rise phase width at 10% of the cardiac cycle to the sum of the ABP/PPG cardiac cycle systolic rise phase width and overall decay phase width at 10% of the cardiac cycle
383	ABP/PPG_W_SRP25	ABP/PPG cardiac cycle systolic rise phase width at 25% of the cardiac cycle
384	ABP/PPG_W_ODP25	ABP/PPG cardiac cycle overall decay phase width at 25% of the cardiac cycle
385	ABP/PPG_W_SRP25_ODP25	Sum of the ABP/PPG cardiac cycle systolic rise phase width and overall decay phase width at 25% of the cardiac cycle
386	ABP/PPG_RT_ODP25_W_SRP25_W	The ratio of the ABP/PPG cardiac cycle overall decay phase width at 25% of the cardiac cycle to the ABP/PPG cardiac cycle systolic rise phase width at 25% of the cardiac cycle
387	ABP/PPG_RT_ODP25_W_SRP25_ODP25_W	The ratio of the ABP/PPG cardiac cycle overall decay phase width at 25% of the cardiac cycle to the sum of the ABP/PPG cardiac cycle systolic rise phase width and overall decay phase width at 25% of the cardiac cycle
388	ABP/PPG_RT_SRP25_W_SRP25_ODP25_W	The ratio of the ABP/PPG cardiac cycle systolic rise phase width at 25% of the cardiac cycle to the sum of the ABP/PPG cardiac cycle systolic rise phase width and overall decay phase width at 25% of the cardiac cycle
389	ABP/PPG_W_SRP33	ABP/PPG cardiac cycle systolic rise phase width at 33% of the cardiac cycle
390	ABP/PPG_W_ODP33	ABP/PPG cardiac cycle overall decay phase width at 33% of the cardiac cycle
391	ABP/PPG_W_SRP33_ODP33	Sum of the ABP/PPG cardiac cycle systolic rise phase width and overall decay phase width at 33% of the cardiac cycle
392	ABP/PPG_RT_ODP33_W_SRP33_W	The ratio of the ABP/PPG cardiac cycle overall decay phase width at 33% of the cardiac cycle to the ABP/PPG cardiac cycle systolic rise phase width at 33% of the cardiac cycle
393	ABP/PPG_RT_ODP33_W_SRP33_ODP33_W	The ratio of the ABP/PPG cardiac cycle overall decay phase width at 33% of the cardiac cycle to the sum of the ABP/PPG cardiac cycle systolic rise phase width and overall decay phase width at 33% of the cardiac cycle

394	ABP/PPG_RT_SRP33_W_SRP33_ODP33_W	The ratio of the ABP/PPG cardiac cycle systolic rise phase width at 33% of the cardiac cycle to the sum of the ABP/PPG cardiac cycle systolic rise phase width and overall decay phase width at 33% of the cardiac cycle
395	ABP/PPG_W_SRP50	ABP/PPG cardiac cycle systolic rise phase width at 50% of the cardiac cycle
396	ABP/PPG_W_ODP50	ABP/PPG cardiac cycle overall decay phase width at 50% of the cardiac cycle
397	ABP/PPG_W_SRP50_ODP50	Sum of the ABP/PPG cardiac cycle systolic rise phase width and overall decay phase width at 50% of the cardiac cycle
398	ABP/PPG_RT_ODP50_W_SRP50_W	The ratio of the ABP/PPG cardiac cycle overall decay phase width at 50% of the cardiac cycle to the ABP/PPG cardiac cycle systolic rise phase width at 50% of the cardiac cycle
399	ABP/PPG_RT_ODP50_W_SRP50_ODP50_W	The ratio of the ABP/PPG cardiac cycle overall decay phase width at 50% of the cardiac cycle to the sum of the ABP/PPG cardiac cycle systolic rise phase width and overall decay phase width at 50% of the cardiac cycle
400	ABP/PPG_RT_SRP50_W_SRP50_ODP50_W	The ratio of the ABP/PPG cardiac cycle systolic rise phase width at 50% of the cardiac cycle to the sum of the ABP/PPG cardiac cycle systolic rise phase width and overall decay phase width at 50% of the cardiac cycle
401	ABP/PPG_W_SRP66	ABP/PPG cardiac cycle systolic rise phase width at 66% of the cardiac cycle
402	ABP/PPG_W_ODP66	ABP/PPG cardiac cycle overall decay phase width at 66% of the cardiac cycle
403	ABP/PPG_W_SRP66_ODP66	Sum of the ABP/PPG cardiac cycle systolic rise phase width and overall decay phase width at 66% of the cardiac cycle
404	ABP/PPG_RT_ODP66_W_SRP66_W	The ratio of the ABP/PPG cardiac cycle overall decay phase width at 66% of the cardiac cycle to the ABP/PPG cardiac cycle systolic rise phase width at 66% of the cardiac cycle
405	ABP/PPG_RT_ODP66_W_SRP66_ODP66_W	The ratio of the ABP/PPG cardiac cycle overall decay phase width at 66% of the cardiac cycle to the sum of the ABP/PPG cardiac cycle systolic rise phase width and overall decay phase width at 66% of the cardiac cycle

406	ABP/PPG_RT_SRP66_W_SRP66_ODP66_W	The ratio of the ABP/PPG cardiac cycle systolic rise phase width at 66% of the cardiac cycle to the sum of the ABP/PPG cardiac cycle systolic rise phase width and overall decay phase width at 66% of the cardiac cycle
407	ABP/PPG_W_SRP75	ABP/PPG cardiac cycle systolic rise phase width at 75% of the cardiac cycle
408	ABP/PPG_W_ODP75	ABP/PPG cardiac cycle overall decay phase width at 75% of the cardiac cycle
409	ABP/PPG_W_SRP75_ODP75	Sum of the ABP/PPG cardiac cycle systolic rise phase width and overall decay phase width at 75% of the cardiac cycle
410	ABP/PPG_RT_ODP75_W_SRP75_W	The ratio of the ABP/PPG cardiac cycle overall decay phase width at 75% of the cardiac cycle to the ABP/PPG cardiac cycle systolic rise phase width at 25% of the cardiac cycle
411	ABP/PPG_RT_ODP75_W_SRP75_ODP75_W	The ratio of the ABP/PPG cardiac cycle overall decay phase width at 75% of the cardiac cycle to the sum of the ABP/PPG cardiac cycle systolic rise phase width and overall decay phase width at 75% of the cardiac cycle
412	ABP/PPG_RT_SRP75_W_SRP75_ODP75_W	The ratio of the ABP/PPG cardiac cycle systolic rise phase width at 75% of the cardiac cycle to the sum of the ABP/PPG cardiac cycle systolic rise phase width and overall decay phase width at 75% of the cardiac cycle

13. Area features (413-422)

413	ABP/PPG_D_Are_SPO_wrtSPP	Area under the ABP/PPG cardiac cycle systolic phase onset with respect to systolic phase peak
414	ABP/PPG_D_Are_SPO_wrtDN	Area under the ABP/PPG cardiac cycle systolic phase onset with respect to dicrotic notch
415	ABP/PPG_D_Are_SPO_wrtDPP	Area under the ABP/PPG cardiac cycle systolic phase onset with respect to diastolic phase peak
416	ABP/PPG_D_Are_SPO_wrtDPE	Area under the ABP/PPG cardiac cycle systolic phase onset with respect to diastolic phase end point
417	ABP/PPG_D_Are_SPP_wrtDN	Area under the ABP/PPG cardiac cycle systolic phase peak with respect to dicrotic notch
418	ABP/PPG_D_Are_SPP_wrtDPP	Area under the ABP/PPG cardiac cycle systolic phase peak with respect to diastolic phase peak

419	ABP/PPG_D_Are_SPP_wrtDPE	Area under the ABP/PPG cardiac cycle systolic phase peak with respect to diastolic phase end point
420	ABP/PPG_D_Are_DN_wrtDPP	Area under the ABP/PPG cardiac cycle dicrotic notch with respect to diastolic phase peak
421	ABP/PPG_D_Are_DN_wrtDPE	Area under the ABP/PPG cardiac cycle dicrotic notch with respect to diastolic phase end point
422	ABP/PPG_D_Are_DPP_wrtDPE	Area under the ABP/PPG cardiac cycle diastolic phase peak with respect to diastolic phase end point

14. Area features with subtracted diastolic end point (423-432)

423	ABP/PPG_D_Are_wsDPE_SPO_wrtSPP	Area under the ABP/PPG cardiac cycle systolic phase onset with respect to systolic phase peak, with subtracted diastolic phase end point
424	ABP/PPG_D_Are_wsDPE_SPO_wrtDN	Area under the ABP/PPG cardiac cycle systolic phase onset with respect to dicrotic notch, with subtracted diastolic phase end point
425	ABP/PPG_D_Are_wsDPE_SPO_wrtDPP	Area under the ABP/PPG cardiac cycle systolic phase onset with respect to diastolic phase peak, with subtracted diastolic phase end point
426	ABP/PPG_D_Are_wsDPE_SPO_wrtDPE	Area under the ABP/PPG cardiac cycle systolic phase onset with respect to diastolic phase end point, with subtracted diastolic phase end point
427	ABP/PPG_D_Are_wsDPE_SPP_wrtDN	Area under the ABP/PPG cardiac cycle systolic phase peak with respect to dicrotic notch, with subtracted diastolic phase end point
428	ABP/PPG_D_Are_wsDPE_SPP_wrtDPP	Area under the ABP/PPG cardiac cycle systolic phase peak with respect to diastolic phase peak, with subtracted diastolic phase end point
429	ABP/PPG_D_Are_wsDPE_SPP_wrtDPE	Area under the ABP/PPG cardiac cycle systolic phase peak with respect to diastolic phase end point, with subtracted diastolic phase end point
430	ABP/PPG_D_Are_wsDPE_DN_wrtDPP	Area under the ABP/PPG cardiac cycle dicrotic notch with respect to diastolic phase peak, with subtracted diastolic phase end point
431	ABP/PPG_D_Are_wsDPE_DN_wrtDPE	Area under the ABP/PPG cardiac cycle dicrotic notch with respect to diastolic phase end point, with subtracted diastolic phase end point
432	ABP/PPG_D_Are_wsDPE_DPP_wrtDPE	Area under the ABP/PPG cardiac cycle diastolic phase peak with respect to diastolic phase end point, with subtracted diastolic phase end point

15. Normalized area features (433-442)

433	ABP/PPG_D_NAre_SPO_wrtSPP	Area under the normalized ABP/PPG cardiac cycle systolic phase onset with respect to systolic phase peak
434	ABP/PPG_D_NAre_SPO_wrtDN	Area under the normalized ABP/PPG cardiac cycle systolic phase onset with respect to dicrotic notch
435	ABP/PPG_D_NAre_SPO_wrtDPP	Area under the normalized ABP/PPG cardiac cycle systolic phase onset with respect to diastolic phase peak
436	ABP/PPG_D_NAre_SPO_wrtDPE	Area under the normalized ABP/PPG cardiac cycle systolic phase onset with respect to diastolic phase end point
437	ABP/PPG_D_NAre_SPP_wrtDN	Area under the normalized ABP/PPG cardiac cycle systolic phase peak with respect to dicrotic notch
438	ABP/PPG_D_NAre_SPP_wrtDPP	Area under the normalized ABP/PPG cardiac cycle systolic phase peak with respect to diastolic phase peak
439	ABP/PPG_D_NAre_SPP_wrtDPE	Area under the normalized ABP/PPG cardiac cycle systolic phase peak with respect to diastolic phase end point
440	ABP/PPG_D_NAre_DN_wrtDPP	Area under the normalized ABP/PPG cardiac cycle dicrotic notch with respect to diastolic phase peak
441	ABP/PPG_D_NAre_DN_wrtDPE	Area under the normalized ABP/PPG cardiac cycle dicrotic notch with respect to diastolic phase end point
442	ABP/PPG_D_NAre_DPP_wrtDPE	Area under the normalized ABP/PPG cardiac cycle diastolic phase peak with respect to diastolic phase end point

16. Normalized area features with subtracted end point (443-452)

443	ABP/PPG_D_NAre_wsDPE_SPO_wrtSPP	Area under the normalized ABP/PPG cardiac cycle systolic phase onset with respect to systolic phase peak, with subtracted diastolic phase end point
444	ABP/PPG_D_NAre_wsDPE_SPO_wrtDN	Area under the normalized ABP/PPG cardiac cycle systolic phase onset with respect to dicrotic notch, with subtracted diastolic phase end point
445	ABP/PPG_D_NAre_wsDPE_SPO_wrtDPP	Area under the normalized ABP/PPG cardiac cycle systolic phase onset with respect to diastolic phase peak, with subtracted diastolic phase end point
446	ABP/PPG_D_NAre_wsDPE_SPO_wrtDPE	Area under the normalized ABP/PPG cardiac cycle systolic phase onset with respect to diastolic phase

		end point, with subtracted diastolic phase end point
447	ABP/PPG_D_NAre_wsDPE_SPP_wrtDN	Area under the normalized ABP/PPG cardiac cycle systolic phase peak with respect to dicrotic notch, with subtracted diastolic phase end point
448	ABP/PPG_D_NAre_wsDPE_SPP_wrtDPP	Area under the normalized ABP/PPG cardiac cycle systolic phase peak with respect to diastolic phase peak, with subtracted diastolic phase end point
449	ABP/PPG_D_NAre_wsDPE_SPP_wrtDPE	Area under the normalized ABP/PPG cardiac cycle systolic phase peak with respect to diastolic phase end point, with subtracted diastolic phase end point
450	ABP/PPG_D_NAre_wsDPE_DN_wrtDPP	Area under the normalized ABP/PPG cardiac cycle dicrotic notch with respect to diastolic phase peak, with subtracted diastolic phase end point
451	ABP/PPG_D_NAre_wsDPE_DN_wrtDPE	Area under the normalized ABP/PPG cardiac cycle dicrotic notch with respect to diastolic phase end point, with subtracted diastolic phase end point
452	ABP/PPG_D_NAre_wsDPE_DPP_wrtDPE	Area under the normalized ABP/PPG cardiac cycle diastolic phase peak with respect to diastolic phase end point, with subtracted diastolic phase end point

17. Area ratio features (453-632)

453	ABP/PPG_D_RT_Are_SPO_wrtDN_ABP/PPG_D_Are_SPO_wrtSPP	The ratio of the area under the ABP/PPG cardiac cycle systolic phase onset with respect to dicrotic notch to the area under the ABP/PPG cardiac cycle systolic phase onset with respect to systolic phase peak
454	ABP/PPG_D_RT_Are_SPO_wrtDPP_ABP/PPG_D_Are_SPO_wrtSPP	The ratio of the area under the ABP/PPG cardiac cycle systolic phase onset with respect to diastolic phase peak to the area under the ABP/PPG cardiac cycle systolic phase onset with respect to systolic phase peak
455	ABP/PPG_D_RT_Are_SPO_wrtDPE_ABP/PPG_D_Are_SPO_wrtSPP	The ratio of the area under the ABP/PPG cardiac cycle systolic phase onset with respect to diastolic phase end point to the area under the ABP/PPG cardiac cycle systolic phase onset with respect to systolic phase peak
456	ABP/PPG_D_RT_Are_SPP_wrtDN_ABP/PPG_D_Are_SPO_wrtSPP	The ratio of the area under the ABP/PPG cardiac cycle systolic phase peak with respect to dicrotic notch to the area under the ABP/PPG

		cardiac cycle systolic phase onset with respect to systolic phase peak
457	ABP/PPG_D_RT_Are_SPP_wrtDPP_ABP/PPG_D_Are_SPO_wrtSPP	The ratio of the area under the ABP/PPG cardiac cycle systolic phase peak with respect to diastolic phase peak to the area under the ABP/PPG cardiac cycle systolic phase onset with respect to systolic phase peak
458	ABP/PPG_D_RT_Are_SPP_wrtDPE_ABP/PPG_D_Are_SPO_wrtSPP	The ratio of the area under the ABP/PPG cardiac cycle systolic phase peak with respect to diastolic phase end point to the area under the ABP/PPG cardiac cycle systolic phase onset with respect to systolic phase peak
459	ABP/PPG_D_RT_Are_DN_wrtDPP_ABP/PPG_D_Are_SPO_wrtSPP	The ratio of the area under the ABP/PPG cardiac cycle dicrotic notch with respect to diastolic phase peak to the area under the ABP/PPG cardiac cycle systolic phase onset with respect to systolic phase peak
460	ABP/PPG_D_RT_Are_DN_wrtDPE_ABP/PPG_D_Are_SPO_wrtSPP	The ratio of the area under the ABP/PPG cardiac cycle dicrotic notch with respect to diastolic phase end point to the area under the ABP/PPG cardiac cycle systolic phase onset with respect to systolic phase peak
461	ABP/PPG_D_RT_Are_DPP_wrtDPE_ABP/PPG_D_Are_SPO_wrtSPP	The ratio of the area under the ABP/PPG cardiac cycle diastolic phase peak with respect to diastolic phase end point to the area under the ABP/PPG cardiac cycle systolic phase onset with respect to systolic phase peak
462	ABP/PPG_D_RT_Are_SPO_wrtDPP_ABP/PPG_D_Are_SPO_wrtDN	The ratio of the area under the ABP/PPG cardiac cycle systolic phase onset with respect to diastolic phase peak to the area under the ABP/PPG cardiac cycle systolic phase onset with respect to dicrotic notch
463	ABP/PPG_D_RT_Are_SPO_wrtDPE_ABP/PPG_D_Are_SPO_wrtDN	The ratio of the area under the ABP/PPG cardiac cycle systolic phase onset with respect to diastolic phase end point to the area under the ABP/PPG cardiac cycle systolic phase onset with respect to dicrotic notch
464	ABP/PPG_D_RT_Are_SPP_wrtDN_ABP/PPG_D_Are_SPO_wrtDN	The ratio of the area under the ABP/PPG cardiac cycle systolic phase peak with respect to dicrotic notch to the area under the ABP/PPG cardiac cycle systolic phase onset with respect to dicrotic notch
465	ABP/PPG_D_RT_Are_SPP_wrtDPP_ABP/PPG_D_Are_SPO_wrtDN	The ratio of the area under the ABP/PPG cardiac cycle systolic phase peak with respect to

		diastolic phase peak to the area under the ABP/PPG cardiac cycle systolic phase onset with respect to dicrotic notch
466	ABP/PPG_D_RT_Are_SPP_wrtDPE_ABP/PPG_D_Are_SPO_wrtDN	The ratio of the area under the ABP/PPG cardiac cycle systolic phase peak with respect to diastolic phase end point to the area under the ABP/PPG cardiac cycle systolic phase onset with respect to dicrotic notch
467	ABP/PPG_D_RT_Are_DN_wrtDPP_ABP/PPG_D_Are_SPO_wrtDN	The ratio of the area under the ABP/PPG cardiac cycle dicrotic notch with respect to diastolic phase peak to the area under the ABP/PPG cardiac cycle systolic phase onset with respect to dicrotic notch
468	ABP/PPG_D_RT_Are_DN_wrtDPE_ABP/PPG_D_Are_SPO_wrtDN	The ratio of the area under the ABP/PPG cardiac cycle dicrotic notch with respect to diastolic phase end point to the area under the ABP/PPG cardiac cycle systolic phase onset with respect to dicrotic notch
469	ABP/PPG_D_RT_Are_DPP_wrtDPE_ABP/PPG_D_Are_SPO_wrtDN	The ratio of the area under the ABP/PPG cardiac cycle diastolic phase peak with respect to diastolic phase end point to the area under the ABP/PPG cardiac cycle systolic phase onset with respect to dicrotic notch
470	ABP/PPG_D_RT_Are_SPO_wrtDPE_ABP/PPG_D_Are_SPO_wrtDPP	The ratio of the area under the ABP/PPG cardiac cycle systolic phase onset with respect to diastolic phase end point to the area under the ABP/PPG cardiac cycle systolic phase onset with respect to diastolic phase peak
471	ABP/PPG_D_RT_Are_SPP_wrtDN_ABP/PPG_D_Are_SPO_wrtDPP	The ratio of the area under the ABP/PPG cardiac cycle systolic phase peak with respect to dicrotic notch to the area under the ABP/PPG cardiac cycle systolic phase onset with respect to diastolic phase peak
472	ABP/PPG_D_RT_Are_SPP_wrtDPP_ABP/PPG_D_Are_SPO_wrtDPP	The ratio of the area under the ABP/PPG cardiac cycle systolic phase peak with respect to diastolic phase peak to the area under the ABP/PPG cardiac cycle systolic phase onset with respect to diastolic phase peak
473	ABP/PPG_D_RT_Are_SPP_wrtDPE_ABP/PPG_D_Are_SPO_wrtDPP	The ratio of the area under the ABP/PPG cardiac cycle systolic phase peak with respect to diastolic phase end point to the area under the ABP/PPG cardiac cycle systolic phase onset with respect to diastolic phase peak

474	ABP/PPG_D_RT_Are_DN_wrtDPP_ABP/PPG_D_Are_SPO_wrtDPP	The ratio of the area under the ABP/PPG cardiac cycle dicrotic notch with respect to diastolic phase peak to the area under the ABP/PPG cardiac cycle systolic phase onset with respect to diastolic phase peak
475	ABP/PPG_D_RT_Are_DN_wrtDPE_ABP/PPG_D_Are_SPO_wrtDPP	The ratio of the area under the ABP/PPG cardiac cycle dicrotic notch with respect to diastolic phase end point to the area under the ABP/PPG cardiac cycle systolic phase onset with respect to diastolic phase peak
476	ABP/PPG_D_RT_Are_DPP_wrtDPE_ABP/PPG_D_Are_SPO_wrtDPP	The ratio of the area under the ABP/PPG cardiac cycle diastolic phase peak with respect to diastolic phase end point to the area under the ABP/PPG cardiac cycle systolic phase onset with respect to diastolic phase peak
477	ABP/PPG_D_RT_Are_SPP_wrtDN_ABP/PPG_D_Are_SPO_wrtDPE	The ratio of the area under the ABP/PPG cardiac cycle systolic phase peak with respect to dicrotic notch to the area under the ABP/PPG cardiac cycle systolic phase onset with respect to diastolic phase end point
478	ABP/PPG_D_RT_Are_SPP_wrtDPP_ABP/PPG_D_Are_SPO_wrtDPE	The ratio of the area under the ABP/PPG cardiac cycle systolic phase peak with respect to diastolic phase peak to the area under the ABP/PPG cardiac cycle systolic phase onset with respect to diastolic phase end point
479	ABP/PPG_D_RT_Are_SPP_wrtDPE_ABP/PPG_D_Are_SPO_wrtDPE	The ratio of the area under the ABP/PPG cardiac cycle systolic phase peak with respect to diastolic phase end point to the area under the ABP/PPG cardiac cycle systolic phase onset with respect to diastolic phase end point
480	ABP/PPG_D_RT_Are_DN_wrtDPP_ABP/PPG_D_Are_SPO_wrtDPE	The ratio of the area under the ABP/PPG cardiac cycle dicrotic notch with respect to diastolic phase peak to the area under the ABP/PPG cardiac cycle systolic phase onset with respect to diastolic phase end point
481	ABP/PPG_D_RT_Are_DN_wrtDPE_ABP/PPG_D_Are_SPO_wrtDPE	The ratio of the area under the ABP/PPG cardiac cycle dicrotic notch with respect to diastolic phase end point to the area under the ABP/PPG cardiac cycle systolic phase onset with respect to diastolic phase end point
482	ABP/PPG_D_RT_Are_DPP_wrtDPE_ABP/PPG_D_Are_SPO_wrtDPE	The ratio of the area under the ABP/PPG cardiac cycle diastolic phase peak with respect to diastolic phase end point to the area under the

		ABP/PPG cardiac cycle systolic phase onset with respect to diastolic phase end point
483	ABP/PPG_D_RT_Are_SPP_wrtDPP_ABP/PPG_D_Are_SPP_wrtDN	The ratio of the area under the ABP/PPG cardiac cycle systolic phase peak with respect to diastolic phase peak to the area under the ABP/PPG cardiac cycle systolic phase peak with respect to dicrotic notch
484	ABP/PPG_D_RT_Are_SPP_wrtDPE_ABP/PPG_D_Are_SPP_wrtDN	The ratio of the area under the ABP/PPG cardiac cycle systolic phase peak with respect to diastolic phase end point to the area under the ABP/PPG cardiac cycle systolic phase peak with respect to dicrotic notch
485	ABP/PPG_D_RT_Are_DN_wrtDPP_ABP/PPG_D_Are_SPP_wrtDN	The ratio of the area under the ABP/PPG cardiac cycle dicrotic notch with respect to diastolic phase peak to the area under the ABP/PPG cardiac cycle systolic phase peak with respect to dicrotic notch
486	ABP/PPG_D_RT_Are_DN_wrtDPE_ABP/PPG_D_Are_SPP_wrtDN	The ratio of the area under the ABP/PPG cardiac cycle dicrotic notch with respect to diastolic phase end point to the area under the ABP/PPG cardiac cycle systolic phase peak with respect to dicrotic notch
487	ABP/PPG_D_RT_Are_DPP_wrtDPE_ABP/PPG_D_Are_SPP_wrtDN	The ratio of the area under the ABP/PPG cardiac cycle diastolic phase peak with respect to diastolic phase end point to the area under the ABP/PPG cardiac cycle systolic phase peak with respect to dicrotic notch
488	ABP/PPG_D_RT_Are_SPP_wrtDPE_ABP/PPG_D_Are_SPP_wrtDPP	The ratio of the area under the ABP/PPG cardiac cycle systolic phase peak with respect to diastolic phase end point to the area under the ABP/PPG cardiac cycle systolic phase peak with respect to diastolic phase peak
489	ABP/PPG_D_RT_Are_DN_wrtDPP_ABP/PPG_D_Are_SPP_wrtDPP	The ratio of the area under the ABP/PPG cardiac cycle dicrotic notch with respect to diastolic phase peak to the area under the ABP/PPG cardiac cycle systolic phase peak with respect to diastolic phase peak
490	ABP/PPG_D_RT_Are_DN_wrtDPE_ABP/PPG_D_Are_SPP_wrtDPP	The ratio of the area under the ABP/PPG cardiac cycle dicrotic notch with respect to diastolic phase end point to the area under the ABP/PPG cardiac cycle systolic phase peak with respect to diastolic phase peak
491	ABP/PPG_D_RT_Are_DPP_wrtDPE_ABP/PPG_D_Are_SPP_wrtDPP	The ratio of the area under the ABP/PPG cardiac cycle diastolic phase peak with respect to

		diastolic phase end point to the area under the ABP/PPG cardiac cycle systolic phase peak with respect to diastolic phase peak
492	ABP/PPG_D_RT_Are_DN_wrtDPP_ABP/PPG_D_Are_SPP_wrtDPE	The ratio of the area under the ABP/PPG cardiac cycle dicrotic notch with respect to diastolic phase peak to the area under the ABP/PPG cardiac cycle systolic phase peak with respect to diastolic phase end point
493	ABP/PPG_D_RT_Are_DN_wrtDPE_ABP/PPG_D_Are_SPP_wrtDPE	The ratio of the area under the ABP/PPG cardiac cycle dicrotic notch with respect to diastolic phase end point to the area under the ABP/PPG cardiac cycle systolic phase peak with respect to diastolic phase end point
494	ABP/PPG_D_RT_Are_DPP_wrtDPE_ABP/PPG_D_Are_SPP_wrtDPE	The ratio of the area under the ABP/PPG cardiac cycle diastolic phase peak with respect to diastolic phase end point to the area under the ABP/PPG cardiac cycle systolic phase peak with respect to diastolic phase end point
495	ABP/PPG_D_RT_Are_DN_wrtDPE_ABP/PPG_D_Are_DN_wrtDPP	The ratio of the area under the ABP/PPG cardiac cycle dicrotic notch with respect to diastolic phase end point to the area under the ABP/PPG cardiac cycle dicrotic notch with respect to diastolic phase peak
496	ABP/PPG_D_RT_Are_DPP_wrtDPE_ABP/PPG_D_Are_DN_wrtDPP	The ratio of the area under the ABP/PPG cardiac cycle diastolic phase peak with respect to diastolic phase end point to the area under the ABP/PPG cardiac cycle dicrotic notch with respect to diastolic phase peak
497	ABP/PPG_D_RT_Are_DPP_wrtDPE_ABP/PPG_D_Are_DN_wrtDPE	The ratio of the area under the ABP/PPG cardiac cycle diastolic phase peak with respect to diastolic phase end point to the area under the ABP/PPG cardiac cycle dicrotic notch with respect to diastolic phase end point
498	ABP/PPG_D_RT_Are_wsDPE_SPO_wrtDN_ABP/PPG_D_Are_wsDPE_SPO_wrtSPP	The ratio of the area under the ABP/PPG cardiac cycle systolic phase onset with respect to dicrotic notch, with subtracted diastolic phase end point to the area under the ABP/PPG cardiac cycle systolic phase onset with respect to systolic phase peak, with subtracted diastolic phase end point
499	ABP/PPG_D_RT_Are_wsDPE_SPO_wrtDPP_ABP/PPG_D_Are_wsDPE_SPO_wrtSPP	The ratio of the area under the ABP/PPG cardiac cycle systolic phase onset with respect to diastolic phase peak, with subtracted diastolic phase end point to the area under the ABP/PPG

		cardiac cycle systolic phase onset with respect to systolic phase peak, with subtracted diastolic phase end point
500	ABP/PPG_D_RT_Are_wsDPE_SPO_wrtDPE_ABP/PPG_D_Are_wsDPE_SPO_wrtSPP	The ratio of the area under the ABP/PPG cardiac cycle systolic phase onset with respect to diastolic phase end point, with subtracted diastolic phase end point to the area under the ABP/PPG cardiac cycle systolic phase onset with respect to systolic phase peak, with subtracted diastolic phase end point
501	ABP/PPG_D_RT_Are_wsDPE_SPP_wrtDN_ABP/PPG_D_Are_wsDPE_SPO_wrtSPP	The ratio of the area under the ABP/PPG cardiac cycle systolic phase peak with respect to dicrotic notch, with subtracted diastolic phase end point to the area under the ABP/PPG cardiac cycle systolic phase onset with respect to systolic phase peak, with subtracted diastolic phase end point
502	ABP/PPG_D_RT_Are_wsDPE_SPP_wrtDPP_ABP/PPG_D_Are_wsDPE_SPO_wrtSPP	The ratio of the area under the ABP/PPG cardiac cycle systolic phase peak with respect to diastolic phase peak, with subtracted diastolic phase end point to the area under the ABP/PPG cardiac cycle systolic phase onset with respect to systolic phase peak, with subtracted diastolic phase end point
503	ABP/PPG_D_RT_Are_wsDPE_SPP_wrtDPE_ABP/PPG_D_Are_wsDPE_SPO_wrtSPP	The ratio of the area under the ABP/PPG cardiac cycle systolic phase peak with respect to diastolic phase end point, with subtracted diastolic phase end point to the area under the ABP/PPG cardiac cycle systolic phase onset with respect to systolic phase peak, with subtracted diastolic phase end point
504	ABP/PPG_D_RT_Are_wsDPE_DN_wrtDPP_ABP/PPG_D_Are_wsDPE_SPO_wrtSPP	The ratio of the area under the ABP/PPG cardiac cycle dicrotic notch with respect to diastolic phase peak, with subtracted diastolic phase end point to the area under the ABP/PPG cardiac cycle systolic phase onset with respect to systolic phase peak, with subtracted diastolic phase end point
505	ABP/PPG_D_RT_Are_wsDPE_DN_wrtDPE_ABP/PPG_D_Are_wsDPE_SPO_wrtSPP	The ratio of the area under the ABP/PPG cardiac cycle dicrotic notch with respect to diastolic phase end point, with subtracted diastolic phase end point to the area under the ABP/PPG cardiac cycle systolic phase onset with respect

		to systolic phase peak, with subtracted diastolic phase end point
506	ABP/PPG_D_RT_Are_wsDPE_DPP_wrtDPE_ABP/PPG_D_Are_wsDPE_SPO_wrtSPP	The ratio of the area under the ABP/PPG cardiac cycle diastolic phase peak with respect to diastolic phase end point, with subtracted diastolic phase end point to the area under the ABP/PPG cardiac cycle systolic phase onset with respect to systolic phase peak, with subtracted diastolic phase end point
507	ABP/PPG_D_RT_Are_wsDPE_SPO_wrtDPP_ABP/PPG_D_Are_wsDPE_SPO_DN	The ratio of the area under the ABP/PPG cardiac cycle systolic phase onset with respect to diastolic phase peak, with subtracted diastolic phase end point to the area under the ABP/PPG cardiac cycle systolic phase onset with respect to dicrotic notch, with subtracted diastolic phase end point
508	ABP/PPG_D_RT_Are_wsDPE_SPO_wrtDPE_ABP/PPG_D_Are_wsDPE_SPO_DN	The ratio of the area under the ABP/PPG cardiac cycle systolic phase onset with respect to diastolic phase end point, with subtracted diastolic phase end point to the area under the ABP/PPG cardiac cycle systolic phase onset with respect to dicrotic notch, with subtracted diastolic phase end point
509	ABP/PPG_D_RT_Are_wsDPE_SPP_wrtDN_ABP/PPG_D_Are_wsDPE_SPO_DN	The ratio of the area under the ABP/PPG cardiac cycle systolic phase peak with respect to dicrotic notch, with subtracted diastolic phase end point to the area under the ABP/PPG cardiac cycle systolic phase onset with respect to dicrotic notch, with subtracted diastolic phase end point
510	ABP/PPG_D_RT_Are_wsDPE_SPP_wrtDPP_ABP/PPG_D_Are_wsDPE_SPO_DN	The ratio of the area under the ABP/PPG cardiac cycle systolic phase peak with respect to diastolic phase peak, with subtracted diastolic phase end point to the area under the ABP/PPG cardiac cycle systolic phase onset with respect to dicrotic notch, with subtracted diastolic phase end point
511	ABP/PPG_D_RT_Are_wsDPE_SPP_wrtDPE_ABP/PPG_D_Are_wsDPE_SPO_DN	The ratio of the area under the ABP/PPG cardiac cycle systolic phase peak with respect to diastolic phase end point, with subtracted diastolic phase end point to the area under the ABP/PPG cardiac cycle systolic phase onset with respect to dicrotic notch, with subtracted diastolic phase end point

512	ABP/PPG_D_RT_Are_wsDPE_DN_wrtDPP_ABP/PPG_D_Are_wsDPE_SPO_DN	The ratio of the area under the ABP/PPG cardiac cycle dicrotic notch with respect to diastolic phase peak, with subtracted diastolic phase end point to the area under the ABP/PPG cardiac cycle systolic phase onset with respect to dicrotic notch, with subtracted diastolic phase end point
513	ABP/PPG_D_RT_Are_wsDPE_DN_wrtDPE_ABP/PPG_D_Are_wsDPE_SPO_DN	The ratio of the area under the ABP/PPG cardiac cycle dicrotic notch with respect to diastolic phase end point, with subtracted diastolic phase end point to the area under the ABP/PPG cardiac cycle systolic phase onset with respect to dicrotic notch, with subtracted diastolic phase end point
514	ABP/PPG_D_RT_Are_wsDPE_DPP_wrtDPE_ABP/PPG_D_Are_wsDPE_SPO_DN	The ratio of the area under the ABP/PPG cardiac cycle diastolic phase peak with respect to diastolic phase end point, with subtracted diastolic phase end point to the area under the ABP/PPG cardiac cycle systolic phase onset with respect to dicrotic notch, with subtracted diastolic phase end point
515	ABP/PPG_D_RT_Are_wsDPE_SPO_wrtDPE_ABP/PPG_D_Are_wsDPE_SPO_wrtDPP	The ratio of the area under the ABP/PPG cardiac cycle systolic phase onset with respect to diastolic phase end point, with subtracted diastolic phase end point to the area under the ABP/PPG cardiac cycle systolic phase onset with respect to diastolic phase peak, with subtracted diastolic phase end point
516	ABP/PPG_D_RT_Are_wsDPE_SPP_wrtDN_ABP/PPG_D_Are_wsDPE_SPO_wrtDPP	The ratio of the area under the ABP/PPG cardiac cycle systolic phase peak with respect to dicrotic notch, with subtracted diastolic phase end point to the area under the ABP/PPG cardiac cycle systolic phase onset with respect to diastolic phase peak, with subtracted diastolic phase end point
517	ABP/PPG_D_RT_Are_wsDPE_SPP_wrtDPP_ABP/PPG_D_Are_wsDPE_SPO_wrtDPP	The ratio of the area under the ABP/PPG cardiac cycle systolic phase peak with respect to diastolic phase peak, with subtracted diastolic phase end point to the area under the ABP/PPG cardiac cycle systolic phase onset with respect to diastolic phase peak, with subtracted diastolic phase end point
518	ABP/PPG_D_RT_Are_wsDPE_SPP_wrtDPE_ABP/PPG_D_Are_wsDPE_SPO_wrtDPP	The ratio of the area under the ABP/PPG cardiac cycle systolic phase peak with respect to

		diastolic phase end point, with subtracted diastolic phase end point to the area under the ABP/PPG cardiac cycle systolic phase onset with respect to diastolic phase peak, with subtracted diastolic phase end point
519	ABP/PPG_D_RT_Are_wsDPE_DN_wrtDPP_ABP/PPG_D_Are_wsDPE_SPO_wrtDPP	The ratio of the area under the ABP/PPG cardiac cycle dicrotic notch with respect to diastolic phase peak, with subtracted diastolic phase end point to the area under the ABP/PPG cardiac cycle systolic phase onset with respect to diastolic phase peak, with subtracted diastolic phase end point
520	ABP/PPG_D_RT_Are_wsDPE_DN_wrtDPE_ABP/PPG_D_Are_wsDPE_SPO_wrtDPP	The ratio of the area under the ABP/PPG cardiac cycle dicrotic notch with respect to diastolic phase end point, with subtracted diastolic phase end point to the area under the ABP/PPG cardiac cycle systolic phase onset with respect to diastolic phase peak, with subtracted diastolic phase end point
521	ABP/PPG_D_RT_Are_wsDPE_DPP_wrtDPE_ABP/PPG_D_Are_wsDPE_SPO_wrtDPP	The ratio of the area under the ABP/PPG cardiac cycle diastolic phase peak with respect to diastolic phase end point, with subtracted diastolic phase end point to the area under the ABP/PPG cardiac cycle systolic phase onset with respect to diastolic phase peak, with subtracted diastolic phase end point
522	ABP/PPG_D_RT_Are_wsDPE_SPP_wrtDN_ABP/PPG_D_Are_wsDPE_SPO_wrtDPE	The ratio of the area under the ABP/PPG cardiac cycle systolic phase peak with respect to dicrotic notch, with subtracted diastolic phase end point to the area under the ABP/PPG cardiac cycle systolic phase onset with respect to diastolic phase end point, with subtracted diastolic phase end point
523	ABP/PPG_D_RT_Are_wsDPE_SPP_wrtDPP_ABP/PPG_D_Are_wsDPE_SPO_wrtDPE	The ratio of the area under the ABP/PPG cardiac cycle systolic phase peak with respect to diastolic phase peak, with subtracted diastolic phase end point to the area under the ABP/PPG cardiac cycle systolic phase onset with respect to diastolic phase end point, with subtracted diastolic phase end point
524	ABP/PPG_D_RT_Are_wsDPE_SPP_wrtDPE_ABP/PPG_D_Are_wsDPE_SPO_wrtDPE	The ratio of the area under the ABP/PPG cardiac cycle systolic phase peak with respect to diastolic phase end point, with subtracted diastolic phase end point to the area under the

		ABP/PPG cardiac cycle systolic phase onset with respect to diastolic phase end point, with subtracted diastolic phase end point
525	ABP/PPG_D_RT_Are_wsDPE_DN_wrtDPP_ABP/PPG_D_Are_wsDPE_SPO_wrtDPE	The ratio of the area under the ABP/PPG cardiac cycle dicrotic notch with respect to diastolic phase peak, with subtracted diastolic phase end point to the area under the ABP/PPG cardiac cycle systolic phase onset with respect to diastolic phase end point, with subtracted diastolic phase end point
526	ABP/PPG_D_RT_Are_wsDPE_DN_wrtDPE_ABP/PPG_D_Are_wsDPE_SPO_wrtDPE	The ratio of the area under the ABP/PPG cardiac cycle dicrotic notch with respect to diastolic phase end point, with subtracted diastolic phase end point to the area under the ABP/PPG cardiac cycle systolic phase onset with respect to diastolic phase end point, with subtracted diastolic phase end point
527	ABP/PPG_D_RT_Are_wsDPE_DPP_wrtDPE_ABP/PPG_D_Are_wsDPE_SPO_wrtDPE	The ratio of the area under the ABP/PPG cardiac cycle diastolic phase peak with respect to diastolic phase end point, with subtracted diastolic phase end point to the area under the ABP/PPG cardiac cycle systolic phase onset with respect to diastolic phase end point, with subtracted diastolic phase end point
528	ABP/PPG_D_RT_Are_wsDPE_SPP_wrtDPP_ABP/PPG_D_Are_wsDPE_SPP_wrtDN	The ratio of the area under the ABP/PPG cardiac cycle systolic phase peak with respect to diastolic phase peak, with subtracted diastolic phase end point to the area under the ABP/PPG cardiac cycle systolic phase peak with respect to dicrotic notch, with subtracted diastolic phase end point
529	ABP/PPG_D_RT_Are_wsDPE_SPP_wrtDPE_ABP/PPG_D_Are_wsDPE_SPP_wrtDN	The ratio of the area under the ABP/PPG cardiac cycle systolic phase peak with respect to diastolic phase end point, with subtracted diastolic phase end point to the area under the ABP/PPG cardiac cycle systolic phase peak with respect to dicrotic notch, with subtracted diastolic phase end point
530	ABP/PPG_D_RT_Are_wsDPE_DN_wrtDPP_ABP/PPG_D_Are_wsDPE_SPP_wrtDN	The ratio of the area under the ABP/PPG cardiac cycle dicrotic notch with respect to diastolic phase end peak, with subtracted diastolic phase end point to the area under the ABP/PPG cardiac cycle systolic phase peak with respect to

		dicrotic notch, with subtracted diastolic phase end point
531	ABP/PPG_D_RT_Are_wsDPE_DN_wrtDPE_ABP/PPG_D_Are_wsDPE_SPP_wrtDN	The ratio of the area under the ABP/PPG cardiac cycle dicrotic notch with respect to diastolic phase end point, with subtracted diastolic phase end point to the area under the ABP/PPG cardiac cycle systolic phase peak with respect to dicrotic notch, with subtracted diastolic phase end point
532	ABP/PPG_D_RT_Are_wsDPE_DPP_wrtDPE_ABP/PPG_D_Are_wsDPE_SPP_wrtDN	The ratio of the area under the ABP/PPG cardiac cycle diastolic phase peak with respect to diastolic phase end point, with subtracted diastolic phase end point to the area under the ABP/PPG cardiac cycle systolic phase peak with respect to dicrotic notch, with subtracted diastolic phase end point
533	ABP/PPG_D_RT_Are_wsDPE_SPP_wrtDPE_ABP/PPG_D_Are_wsDPE_SPP_wrtDPP	The ratio of the area under the ABP/PPG cardiac cycle systolic phase peak with respect to diastolic phase end point, with subtracted diastolic phase end point to the area under the ABP/PPG cardiac cycle systolic phase peak with respect to diastolic phase peak, with subtracted diastolic phase end point
534	ABP/PPG_D_RT_Are_wsDPE_DN_wrtDPP_ABP/PPG_D_Are_wsDPE_SPP_wrtDPP	The ratio of the area under the ABP/PPG cardiac cycle dicrotic notch with respect to diastolic phase peak, with subtracted diastolic phase end point to the area under the ABP/PPG cardiac cycle systolic phase peak with respect to diastolic phase peak, with subtracted diastolic phase end point
535	ABP/PPG_D_RT_Are_wsDPE_DN_wrtDPE_ABP/PPG_D_Are_wsDPE_SPP_wrtDPP	The ratio of the area under the ABP/PPG cardiac cycle dicrotic notch with respect to diastolic phase end point, with subtracted diastolic phase end point to the area under the ABP/PPG cardiac cycle systolic phase peak with respect to diastolic phase peak, with subtracted diastolic phase end point
536	ABP/PPG_D_RT_Are_wsDPE_DPP_wrtDPE_ABP/PPG_D_Are_wsDPE_SPP_wrtDPP	The ratio of the area under the ABP/PPG cardiac cycle diastolic phase peak with respect to diastolic phase end point, with subtracted diastolic phase end point to the area under the ABP/PPG cardiac cycle systolic phase peak with respect to diastolic phase peak, with subtracted diastolic phase end point

537	ABP/PPG_D_RT_Are_wsDPE_DN_wrtDPP_ABP/PPG_D_Are_wsDPE_SPP_wrtDPE	The ratio of the area under the ABP/PPG cardiac cycle dicrotic notch with respect to diastolic phase peak, with subtracted diastolic phase end point to the area under the ABP/PPG cardiac cycle systolic phase peak with respect to diastolic phase end point, with subtracted diastolic phase end point
538	ABP/PPG_D_RT_Are_wsDPE_DN_wrtDPE_ABP/PPG_D_Are_wsDPE_SPP_wrtDPE	The ratio of the area under the ABP/PPG cardiac cycle dicrotic notch with respect to diastolic phase end point, with subtracted diastolic phase end point to the area under the ABP/PPG cardiac cycle systolic phase peak with respect to diastolic phase end point, with subtracted diastolic phase end point
539	ABP/PPG_D_RT_Are_wsDPE_DPP_wrtDPE_ABP/PPG_D_Are_wsDPE_SPP_wrtDPE	The ratio of the area under the ABP/PPG cardiac cycle diastolic phase peak with respect to diastolic phase end point, with subtracted diastolic phase end point to the area under the ABP/PPG cardiac cycle systolic phase peak with respect to diastolic phase end point, with subtracted diastolic phase end point
540	ABP/PPG_D_RT_Are_wsDPE_DN_wrtDPE_ABP/PPG_D_Are_wsDPE_DN_wrtDPP	The ratio of the area under the ABP/PPG cardiac cycle dicrotic notch with respect to diastolic phase end point, with subtracted diastolic phase end point to the area under the ABP/PPG cardiac cycle dicrotic notch with respect to diastolic phase peak, with subtracted diastolic phase end point
541	ABP/PPG_D_RT_Are_wsDPE_DPP_wrtDPE_ABP/PPG_D_Are_wsDPE_DN_wrtDPP	The ratio of the area under the ABP/PPG cardiac cycle diastolic phase peak with respect to diastolic phase end point, with subtracted diastolic phase end point to the area under the ABP/PPG cardiac cycle dicrotic notch with respect to diastolic phase peak, with subtracted diastolic phase end point
542	ABP/PPG_D_RT_Are_wsDPE_DPP_wrtDPE_ABP/PPG_D_Are_wsDPE_DN_wrtDPE	The ratio of the area under the ABP/PPG cardiac cycle diastolic phase peak with respect to diastolic phase end point, with subtracted diastolic phase end point to the area under the ABP/PPG cardiac cycle dicrotic notch with respect to diastolic phase end point, with subtracted diastolic phase end point
543	ABP/PPG_D_RT_NAre_SPO_wrtDN_ABP/PPG_D_NAre_SPO_wrtSPP	The ratio of the area under the normalized ABP/PPG cardiac cycle systolic phase onset with

		respect to dicrotic notch to the area under the normalized ABP/PPG cardiac cycle systolic phase onset with respect to systolic phase peak
544	ABP/PPG_D_RT_NAre_SPO_wrtDPP_ABP/PPG_D_NAre_SPO_wrtSPP	The ratio of the area under the normalized ABP/PPG cardiac cycle systolic phase onset with respect to diastolic phase peak to the area under the normalized ABP/PPG cardiac cycle systolic phase onset with respect to systolic phase peak
545	ABP/PPG_D_RT_NAre_SPO_wrtDPE_ABP/PPG_D_NAre_SPO_wrtSPP	The ratio of the area under the normalized ABP/PPG cardiac cycle systolic phase onset with respect to diastolic phase end point to the area under the normalized ABP/PPG cardiac cycle systolic phase onset with respect to systolic phase peak
546	ABP/PPG_D_RT_NAre_SPP_wrtDN_ABP/PPG_D_NAre_SPO_wrtSPP	The ratio of the area under the normalized ABP/PPG cardiac cycle systolic phase peak with respect to dicrotic notch to the area under the normalized ABP/PPG cardiac cycle systolic phase onset with respect to systolic phase peak
547	ABP/PPG_D_RT_NAre_SPP_wrtDPP_ABP/PPG_D_NAre_SPO_wrtSPP	The ratio of the area under the normalized ABP/PPG cardiac cycle systolic phase peak with respect to diastolic phase peak to the area under the normalized ABP/PPG cardiac cycle systolic phase onset with respect to systolic phase peak
548	ABP/PPG_D_RT_NAre_SPP_wrtDPE_ABP/PPG_D_NAre_SPO_wrtSPP	The ratio of the area under the normalized ABP/PPG cardiac cycle systolic phase peak with respect to diastolic phase end point to the area under the normalized ABP/PPG cardiac cycle systolic phase onset with respect to systolic phase peak
549	ABP/PPG_D_RT_NAre_DN_wrtDPP_ABP/PPG_D_NAre_SPO_wrtSPP	The ratio of the area under the normalized ABP/PPG cardiac cycle dicrotic notch with respect to diastolic phase peak to the area under the normalized ABP/PPG cardiac cycle systolic phase onset with respect to systolic phase peak
550	ABP/PPG_D_RT_NAre_DN_wrtDPE_ABP/PPG_D_NAre_SPO_wrtSPP	The ratio of the area under the normalized ABP/PPG cardiac cycle dicrotic notch with respect to diastolic phase end point to the area under the normalized ABP/PPG cardiac cycle systolic phase onset with respect to systolic phase peak

551	ABP/PPG_D_RT_NAre_DPP_wrtDPE_ABP/PPG_D_NAre_SPO_wrtSPP	The ratio of the area under the normalized ABP/PPG cardiac cycle diastolic phase peak with respect to diastolic phase end point to the area under the normalized ABP/PPG cardiac cycle systolic phase onset with respect to systolic phase peak
552	ABP/PPG_D_RT_NAre_SPO_wrtDPP_ABP/PPG_D_NAre_SPO_wrtDN	The ratio of the area under the normalized ABP/PPG cardiac cycle systolic phase onset with respect to diastolic phase peak to the area under the normalized ABP/PPG cardiac cycle systolic phase onset with respect to dicrotic notch
553	ABP/PPG_D_RT_NAre_SPO_wrtDPE_ABP/PPG_D_NAre_SPO_wrtDN	The ratio of the area under the normalized ABP/PPG cardiac cycle systolic phase onset with respect to diastolic phase end point to the area under the normalized ABP/PPG cardiac cycle systolic phase onset with respect to dicrotic notch
554	ABP/PPG_D_RT_NAre_SPP_wrtDN_ABP/PPG_D_NAre_SPO_wrtDN	The ratio of the area under the normalized ABP/PPG cardiac cycle systolic phase peak with respect to dicrotic notch to the area under the normalized ABP/PPG cardiac cycle systolic phase onset with respect to dicrotic notch
555	ABP/PPG_D_RT_NAre_SPP_wrtDPP_ABP/PPG_D_NAre_SPO_wrtDN	The ratio of the area under the normalized ABP/PPG cardiac cycle systolic phase peak with respect to diastolic phase peak to the area under the normalized ABP/PPG cardiac cycle systolic phase onset with respect to dicrotic notch
556	ABP/PPG_D_RT_NAre_SPP_wrtDPE_ABP/PPG_D_NAre_SPO_wrtDN	The ratio of the area under the normalized ABP/PPG cardiac cycle systolic phase peak with respect to diastolic phase end point to the area under the normalized ABP/PPG cardiac cycle systolic phase onset with respect to dicrotic notch
557	ABP/PPG_D_RT_NAre_DN_wrtDPP_ABP/PPG_D_NAre_SPO_wrtDN	The ratio of the area under the normalized ABP/PPG cardiac cycle dicrotic notch with respect to diastolic phase peak to the area under the normalized ABP/PPG cardiac cycle systolic phase onset with respect to dicrotic notch
558	ABP/PPG_D_RT_NAre_DN_wrtDPE_ABP/PPG_D_NAre_SPO_wrtDN	The ratio of the area under the normalized ABP/PPG cardiac cycle dicrotic notch with respect to diastolic phase end point to the area

		under the normalized ABP/PPG cardiac cycle systolic phase onset with respect to dicrotic notch
559	ABP/PPG_D_RT_NAre_DPP_wrtDPE_ABP/PPG_D_NAre_SPO_wrtDN	The ratio of the area under the normalized ABP/PPG cardiac cycle diastolic phae peak with respect to diastolic phase end point to the area under the normalized ABP/PPG cardiac cycle systolic phase onset with respect to dicrotic notch
560	ABP/PPG_D_RT_NAre_SPO_wrtDPE_ABP/PPG_D_NAre_SPO_wrtDPP	The ratio of the area under the normalized ABP/PPG cardiac cycle systolic phase onset with respect to diastolic phase end point to the area under the normalized ABP/PPG cardiac cycle systolic phase onset with respect to diastolic phase peak
561	ABP/PPG_D_RT_NAre_SPP_wrtDN_ABP/PPG_D_NAre_SPO_wrtDPP	The ratio of the area under the normalized ABP/PPG cardiac cycle systolic phase peak with respect to dicrotic notch to the area under the normalized ABP/PPG cardiac cycle systolic phase onset with respect to diastolic phase peak
562	ABP/PPG_D_RT_NAre_SPP_wrtDPP_ABP/PPG_D_NAre_SPO_wrtDPP	The ratio of the area under the normalized ABP/PPG cardiac cycle systolic phase peak with respect to diastolic phase peak to the area under the normalized ABP/PPG cardiac cycle systolic phase onset with respect to diastolic phase peak
563	ABP/PPG_D_RT_NAre_SPP_wrtDPE_ABP/PPG_D_NAre_SPO_wrtDPP	The ratio of the area under the normalized ABP/PPG cardiac cycle systolic phase peak with respect to diastolic phase end point to the area under the normalized ABP/PPG cardiac cycle systolic phase onset with respect to diastolic phase peak
564	ABP/PPG_D_RT_NAre_DN_wrtDPP_ABP/PPG_D_NAre_SPO_wrtDPP	The ratio of the area under the normalized ABP/PPG cardiac cycle dicrotic notch with respect to diastolic phase peak to the area under the normalized ABP/PPG cardiac cycle systolic phase onset with respect to diastolic phase peak
565	ABP/PPG_D_RT_NAre_DN_wrtDPE_ABP/PPG_D_NAre_SPO_wrtDPP	The ratio of the area under the normalized ABP/PPG cardiac cycle dicrotic notch with respect to diastolic phase end point to the area under the normalized ABP/PPG cardiac cycle

		systolic phase onset with respect to diastolic phase peak
566	ABP/PPG_D_RT_NAre_DPP_wrtDPE_ABP/PPG_D_NAre_SPO_wrtDPP	The ratio of the area under the normalized ABP/PPG cardiac cycle diastolic phase peak with respect to diastolic phase end point to the area under the normalized ABP/PPG cardiac cycle systolic phase onset with respect to diastolic phase peak
567	ABP/PPG_D_RT_NAre_SPP_wrtDN_ABP/PPG_D_NAre_SPO_wrtDPE	The ratio of the area under the normalized ABP/PPG cardiac cycle systolic phase peak with respect to dicrotic notch to the area under the normalized ABP/PPG cardiac cycle systolic phase onset with respect to diastolic phase end point
568	ABP/PPG_D_RT_NAre_SPP_wrtDPP_ABP/PPG_D_NAre_SPO_wrtDPE	The ratio of the area under the normalized ABP/PPG cardiac cycle systolic phase peak with respect to diastolic phase peak to the area under the normalized ABP/PPG cardiac cycle systolic phase onset with respect to diastolic phase end point
569	ABP/PPG_D_RT_NAre_SPP_wrtDPE_ABP/PPG_D_NAre_SPO_wrtDPE	The ratio of the area under the normalized ABP/PPG cardiac cycle systolic phase peak with respect to diastolic phase end point to the area under the normalized ABP/PPG cardiac cycle systolic phase onset with respect to diastolic phase end point
570	ABP/PPG_D_RT_NAre_DN_wrtDPP_ABP/PPG_D_NAre_SPO_wrtDPE	The ratio of the area under the normalized ABP/PPG cardiac cycle dicrotic notch peak with respect to diastolic phase peak to the area under the normalized ABP/PPG cardiac cycle systolic phase onset with respect to diastolic phase end point
571	ABP/PPG_D_RT_NAre_DN_wrtDPE_ABP/PPG_D_NAre_SPO_wrtDPE	The ratio of the area under the normalized ABP/PPG cardiac cycle dicrotic notch with respect to diastolic phase end point to the area under the normalized ABP/PPG cardiac cycle systolic phase onset with respect to diastolic phase end point
572	ABP/PPG_D_RT_NAre_DPP_wrtDPE_ABP/PPG_D_NAre_SPO_wrtDPE	The ratio of the area under the normalized ABP/PPG cardiac cycle diastolic phase peak with respect to diastolic phase end point to the area under the normalized ABP/PPG cardiac cycle systolic phase onset with respect to diastolic phase end point

573	ABP/PPG_D_RT_NAre_SPP_wrtDPP_ABP/PPG_D_NAre_SPP_wrtDN	The ratio of the area under the normalized ABP/PPG cardiac cycle systolic phase peak with respect to diastolic phase peak to the area under the normalized ABP/PPG cardiac cycle systolic phase peak with respect to dicrotic notch
574	ABP/PPG_D_RT_NAre_SPP_wrtDPE_ABP/PPG_D_NAre_SPP_wrtDN	The ratio of the area under the normalized ABP/PPG cardiac cycle systolic phase peak with respect to diastolic phase end point to the area under the normalized ABP/PPG cardiac cycle systolic phase peak with respect to dicrotic notch
575	ABP/PPG_D_RT_NAre_DN_wrtDPP_ABP/PPG_D_NAre_SPP_wrtDN	The ratio of the area under the normalized ABP/PPG cardiac cycle dicrotic notch with respect to diastolic phase peak to the area under the normalized ABP/PPG cardiac cycle systolic phase peak with respect to dicrotic notch
576	ABP/PPG_D_RT_NAre_DN_wrtDPE_ABP/PPG_D_NAre_SPP_wrtDN	The ratio of the area under the normalized ABP/PPG cardiac cycle dicrotic notch with respect to diastolic phase end point to the area under the normalized ABP/PPG cardiac cycle systolic phase peak with respect to dicrotic notch
577	ABP/PPG_D_RT_NAre_DPP_wrtDPE_ABP/PPG_D_NAre_SPP_wrtDN	The ratio of the area under the normalized ABP/PPG cardiac cycle diastolic phase peak with respect to diastolic phase end point to the area under the normalized ABP/PPG cardiac cycle systolic phase peak with respect to dicrotic notch
578	ABP/PPG_D_RT_NAre_SPP_wrtDPE_ABP/PPG_D_NAre_SPP_wrtDPP	The ratio of the area under the normalized ABP/PPG cardiac cycle systolic phase peak with respect to diastolic phase end point to the area under the normalized ABP/PPG cardiac cycle systolic phase peak with respect to diastolic phase peak
579	ABP/PPG_D_RT_NAre_DN_wrtDPP_ABP/PPG_D_NAre_SPP_wrtDPP	The ratio of the area under the normalized ABP/PPG cardiac cycle dicrotic notch with respect to diastolic phase peak to the area under the normalized ABP/PPG cardiac cycle systolic phase peak with respect to diastolic phase peak
580	ABP/PPG_D_RT_NAre_DN_wrtDPE_ABP/PPG_D_NAre_SPP_wrtDPP	The ratio of the area under the normalized ABP/PPG cardiac cycle dicrotic notch with

		respect to diastolic phase end point to the area under the normalized ABP/PPG cardiac cycle systolic phase peak with respect to diastolic phase peak
581	ABP/PPG_D_RT_NAre_DPP_wrtDPE_ABP/PPG_D_NAre_SPP_wrtDPP	The ratio of the area under the normalized ABP/PPG cardiac cycle diastolic phase peak with respect to diastolic phase end point to the area under the normalized ABP/PPG cardiac cycle systolic phase peak with respect to diastolic phase peak
582	ABP/PPG_D_RT_NAre_DN_wrtDPP_ABP/PPG_D_NAre_SPP_wrtDPE	The ratio of the area under the normalized ABP/PPG cardiac cycle dicrotic notch with respect to diastolic phase peak to the area under the normalized ABP/PPG cardiac cycle systolic phase peak with respect to diastolic phase end point
583	ABP/PPG_D_RT_NAre_DN_wrtDPE_ABP/PPG_D_NAre_SPP_wrtDPE	The ratio of the area under the normalized ABP/PPG cardiac cycle dicrotic notch with respect to diastolic phase end point to the area under the normalized ABP/PPG cardiac cycle systolic phase peak with respect to diastolic phase end point
584	ABP/PPG_D_RT_NAre_DPP_wrtDPE_ABP/PPG_D_NAre_SPP_wrtDPE	The ratio of the area under the normalized ABP/PPG cardiac cycle diastolic phase peak with respect to diastolic phase end point to the area under the normalized ABP/PPG cardiac cycle systolic phase peak with respect to diastolic phase end point
585	ABP/PPG_D_RT_NAre_DN_wrtDPE_ABP/PPG_D_NAre_DN_wrtDPP	The ratio of the area under the normalized ABP/PPG cardiac cycle dicrotic notch with respect to diastolic phase end point to the area under the normalized ABP/PPG cardiac cycle dicrotic notch with respect to diastolic phase peak
586	ABP/PPG_D_RT_NAre_DPP_wrtDPE_ABP/PPG_D_NAre_DN_wrtDPP	The ratio of the area under the normalized ABP/PPG cardiac cycle diastolic phase peak with respect to diastolic phase end point to the area under the normalized ABP/PPG cardiac cycle dicrotic notch with respect to diastolic phase peak
587	ABP/PPG_D_RT_NAre_DPP_wrtDPE_ABP/PPG_D_NAre_DN_wrtDPE	The ratio of the area under the normalized ABP/PPG cardiac cycle diastolic phase peak with respect to diastolic phase end point to the area under the normalized ABP/PPG cardiac cycle

		dicrotic notch with respect to diastolic phase end point
588	ABP/PPG_D_RT_NAre_wsDPE_SPO_wrtDN_ABP/PPG_D_NAre_wsDPE_SPO_wrtSPP	The ratio of the area under the normalized ABP/PPG cardiac cycle systolic phase onset with respect to dicrotic notch duration, with subtracted diastolic phase end point to the area under the normalized ABP/PPG cardiac cycle systolic phase onset with respect to systolic phase peak, with subtracted diastolic phase end point
589	ABP/PPG_D_RT_NAre_wsDPE_SPO_wrtDPP_ABP/PPG_D_NAre_wsDPE_SPO_wrtSPP	The ratio of the area under the normalized ABP/PPG cardiac cycle systolic phase onset with respect to diastolic phase peak, with subtracted diastolic phase end point to the area under the normalized ABP/PPG cardiac cycle systolic phase onset with respect to systolic phase peak, with subtracted diastolic phase end point
590	ABP/PPG_D_RT_NAre_WsDPE_SPO_WrtDPE_ABP/PPG_D_NAre_wsDPE_SPO_wrtSPP	The ratio of the area under the normalized ABP/PPG cardiac cycle systolic phase onset with respect to diastolic phase end point, with subtracted diastolic phase end point to the area under the normalized ABP/PPG cardiac cycle systolic phase onset with respect to systolic phase peak, with subtracted diastolic phase end point
591	ABP/PPG_D_RT_NAre_wsDPE_SPP_wrtDN_ABP/PPG_D_NAre_wsDPE_SPO_wrtSPP	The ratio of the area under the normalized ABP/PPG cardiac cycle systolic phase peak with respect to dicrotic notch, with subtracted diastolic phase end point to the area under the normalized ABP/PPG cardiac cycle systolic phase onset with respect to systolic phase peak, with subtracted diastolic phase end point
592	ABP/PPG_D_RT_NAre_wsDPE_SPP_wrtDPP_ABP/PPG_D_NAre_wsDPE_SPO_wrtSPP	The ratio of the area under the normalized ABP/PPG cardiac cycle systolic phase peak with respect to diastolic phase peak, with subtracted diastolic phase end point to the area under the normalized ABP/PPG cardiac cycle systolic phase onset with respect to systolic phase peak, with subtracted diastolic phase end point
593	ABP/PPG_D_RT_NAre_wsDPE_SPP_wrtDPE_ABP/PPG_D_NAre_wsDPE_SPO_wrtSPP	The ratio of the area under the normalized ABP/PPG cardiac cycle systolic phase peak with respect to diastolic phase end point, with subtracted diastolic phase end point to the area under the normalized ABP/PPG cardiac cycle

		systolic phase onset with respect to systolic phase peak, with subtracted diastolic phase end point
594	ABP/PPG_D_RT_NAre_wsDPE_DN_wrtDPP_ABP/PPG_D_NAre_wsDPE_SPO_wrtSPP	The ratio of the area under the normalized ABP/PPG cardiac cycle dicrotic notch with respect to diastolic phase peak, with subtracted diastolic phase end point to the area under the normalized ABP/PPG cardiac cycle systolic phase onset with respect to systolic phase peak, with subtracted diastolic phase end point
595	ABP/PPG_D_RT_NAre_wsDPE_DN_wrtDPE_ABP/PPG_D_NAre_wsDPE_SPO_wrtSPP	The ratio of the area under the normalized ABP/PPG cardiac cycle dicrotic notch with respect to diastolic phase end point, with subtracted diastolic phase end point to the area under the normalized ABP/PPG cardiac cycle systolic phase onset with respect to systolic phase peak, with subtracted diastolic phase end point
596	ABP/PPG_D_RT_NAre_wsDPE_DPP_wrtDPE_ABP/PPG_D_NAre_wsDPE_SPO_wrtSPP	The ratio of the area under the normalized ABP/PPG cardiac cycle diastolic phase peak with respect to diastolic phase end point, with subtracted diastolic phase end point to the area under the normalized ABP/PPG cardiac cycle systolic phase onset with respect to systolic phase peak, with subtracted diastolic phase end point
597	ABP/PPG_D_RT_NAre_wsDPE_SPO_wrtDPP_ABP/PPG_D_NAre_wsDPE_SPO_wrtDN	The ratio of the area under the normalized ABP/PPG cardiac cycle systolic phase onset with respect to diastolic phase peak, with subtracted diastolic phase end point to the area under the normalized ABP/PPG cardiac cycle systolic phase onset with respect to dicrotic notch, with subtracted diastolic phase end point
598	ABP/PPG_D_RT_NAre_wsDPE_SPO_wrtDPE_ABP/PPG_D_NAre_wsDPE_SPO_wrtDN	The ratio of the area under the normalized ABP/PPG cardiac cycle systolic phase onset with respect to diastolic phase end point, with subtracted diastolic phase end point to the area under the normalized ABP/PPG cardiac cycle systolic phase onset with respect to dicrotic notch, with subtracted diastolic phase end point
599	ABP/PPG_D_RT_NAre_wsDPE_SPP_wrtDN_ABP/PPG_D_NAre_wsDPE_SPO_wrtDN	The ratio of the area under the normalized ABP/PPG cardiac cycle systolic phase peak with respect to dicrotic notch, with subtracted diastolic phase end point to the area under the

		normalized ABP/PPG cardiac cycle systolic phase onset with respect to dicrotic notch, with subtracted diastolic phase end point
600	ABP/PPG_D_RT_NAre_wsDPE_SPP_wrtDPP_ABP/PPG_D_NAre_wsDPE_SPO_wrtDN	The ratio of the area under the normalized ABP/PPG cardiac cycle systolic phase peak with respect to diastolic phase peak, with subtracted diastolic phase end point to the area under the normalized ABP/PPG cardiac cycle systolic phase onset with respect to dicrotic notch, with subtracted diastolic phase end point
601	ABP/PPG_D_RT_NAre_wsDPE_SPP_wrtDPE_ABP/PPG_D_NAre_wsDPE_SPO_wrtDN	The ratio of the area under the normalized ABP/PPG cardiac cycle systolic phase peak with respect to diastolic phase end point, with subtracted diastolic phase end point to the area under the normalized ABP/PPG cardiac cycle systolic phase onset with respect to dicrotic notch, with subtracted diastolic phase end point
602	ABP/PPG_D_RT_NAre_wsDPE_DN_wrtDPP_ABP/PPG_D_NAre_wsDPE_SPO_wrtDN	The ratio of the area under the normalized ABP/PPG cardiac cycle dicrotic notch with respect to diastolic phase peak, with subtracted diastolic phase end point to the area under the normalized ABP/PPG cardiac cycle systolic phase onset with respect to dicrotic notch, with subtracted diastolic phase end point
603	ABP/PPG_D_RT_NAre_wsDPE_DN_wrtDPE_ABP/PPG_D_NAre_wsDPE_SPO_wrtDN	The ratio of the area under the normalized ABP/PPG cardiac cycle dicrotic notch with respect to diastolic phase end point, with subtracted diastolic phase end point to the area under the normalized ABP/PPG cardiac cycle systolic phase onset with respect to dicrotic notch, with subtracted diastolic phase end point
604	ABP/PPG_D_RT_NAre_wsDPE_DPP_wrtDPE_ABP/PPG_D_NAre_wsDPE_SPO_wrtDN	The ratio of the area under the normalized ABP/PPG cardiac cycle diastolic phase peak with respect to diastolic phase end point, with subtracted diastolic phase end point to the area under the normalized ABP/PPG cardiac cycle systolic phase onset with respect to dicrotic notch, with subtracted diastolic phase end point
605	ABP/PPG_D_RT_NAre_wsDPE_SPO_wrtDPE_ABP/PPG_D_NAre_wsDPE_SPO_wrtDPP	The ratio of normalized area of systolic phase onset with respect to diastolic phase end point, with subtracted diastolic phase end point to the area under the normalized ABP/PPG cardiac cycle systolic phase onset with respect to

		diastolic phase peak, with subtracted diastolic phase end point
606	ABP/PPG_D_RT_NAre_wsDPE_SPP_wrtDN_ABP/PPG_D_NAre_wsDPE_SPO_wrtDPP	The ratio of normalized area of systolic phase peak with respect to dicrotic notch, with subtracted diastolic phase end point to the area under the normalized ABP/PPG cardiac cycle systolic phase onset with respect to diastolic phase peak, with subtracted diastolic phase end point
607	ABP/PPG_D_RT_NAre_wsDPE_SPP_wrtDPP_ABP/PPG_D_NAre_wsDPE_SPO_wrtDPP	The ratio of the area under the normalized ABP/PPG cardiac cycle systolic phase peak with respect to diastolic phase peak, with subtracted diastolic phase end point to the area under the normalized ABP/PPG cardiac cycle systolic phase onset with respect to diastolic phase peak, with subtracted diastolic phase end point
608	ABP/PPG_D_RT_NAre_wsDPE_SPP_wrtDPE_ABP/PPG_D_NAre_wsDPE_SPO_wrtDPP	The ratio of normalized area of systolic phase peak with respect to diastolic phase end point, with subtracted diastolic phase end point to the area under the normalized ABP/PPG cardiac cycle systolic phase onset with respect to diastolic phase peak, with subtracted diastolic phase end point
609	ABP/PPG_D_RT_NAre_wsDPE_DN_wrtDPP_ABP/PPG_D_NAre_wsDPE_SPO_wrtDPP	The ratio of the area under the normalized ABP/PPG cardiac cycle dicrotic notch with respect to diastolic phase peak, with subtracted diastolic phase end point to the area under the normalized ABP/PPG cardiac cycle systolic phase onset with respect to diastolic phase peak, with subtracted diastolic phase end point
610	ABP/PPG_D_RT_NAre_wsDPE_DN_wrtDPE_ABP/PPG_D_NAre_wsDPE_SPO_wrtDPP	The ratio of normalized area of dicrotic notch with respect to diastolic phase end point, with subtracted diastolic phase end point to the area under the normalized ABP/PPG cardiac cycle systolic phase onset with respect to diastolic phase peak, with subtracted diastolic phase end point
611	ABP/PPG_D_RT_NAre_wsDPE_DPP_wrtDPE_ABP/PPG_D_NAre_wsDPE_SPO_wrtDPP	The ratio of normalized area of diastolic phase peak with respect to diastolic phase end point, with subtracted diastolic phase end point to the area under the normalized ABP/PPG cardiac cycle systolic phase onset with respect to diastolic phase peak, with subtracted diastolic phase end point

612	ABP/PPG_D_RT_NAre_wsDPE_SPP_wrtDN_ABP/PPG_D_NAre_wsDPE_SPO_wrtDPE	The ratio of normalized area of systolic phase peak with respect to dicrotic notch, with subtracted diastolic phase end point to the area under the normalized ABP/PPG cardiac cycle systolic phase onset with respect to diastolic phase end point, with subtracted diastolic phase end point
613	ABP/PPG_D_RT_NAre_wsDPE_SPP_wrtDPP_ABP/PPG_D_NAre_wsDPE_SPO_wrtDPE	The ratio of the area under the normalized ABP/PPG cardiac cycle systolic phase peak with respect to diastolic phase peak, with subtracted diastolic phase end point to the area under the normalized ABP/PPG cardiac cycle systolic phase onset with respect to diastolic phase end point, with subtracted diastolic phase end point
614	ABP/PPG_D_RT_NAre_wsDPE_SPP_wrtDPE_ABP/PPG_D_NAre_wsDPE_SPO_wrtDPE	The ratio of the area under the normalized ABP/PPG cardiac cycle systolic phase peak with respect to diastolic phase end point, with subtracted diastolic phase end point to the area under the normalized ABP/PPG cardiac cycle systolic phase onset with respect to diastolic phase end point, with subtracted diastolic phase end point
615	ABP/PPG_D_RT_NAre_wsDPE_DN_wrtDPP_ABP/PPG_D_NAre_wsDPE_SPO_wrtDPE	The ratio of the area under the normalized ABP/PPG cardiac cycle dicrotic notch with respect to diastolic phase peak, with subtracted diastolic phase end point to the area under the normalized ABP/PPG cardiac cycle systolic phase onset with respect to diastolic phase end point
616	ABP/PPG_D_RT_NAre_wsDPE_DN_wrtDPE_ABP/PPG_D_NAre_wsDPE_SPO_wrtDPE	The ratio of the area under the normalized ABP/PPG cardiac cycle dicrotic notch with respect to diastolic phase end point, with subtracted diastolic phase end point to the area under the normalized ABP/PPG cardiac cycle systolic phase onset with respect to diastolic phase end point, with subtracted diastolic phase end point
617	ABP/PPG_D_RT_NAre_wsDPE_DPP_wrtDPE_ABP/PPG_D_NAre_wsDPE_SPO_wrtDPE	The ratio of the area under the normalized ABP/PPG cardiac cycle diastolic phase peak with respect to diastolic phase end point, with subtracted diastolic phase end point to the area under the normalized ABP/PPG cardiac cycle systolic phase onset with respect to diastolic

		phase end point, with subtracted diastolic phase end point
618	ABP/PPG_D_RT_NAre_wsDPE_SPP_wrtDPP_ABP/PPG_D_NAre_wsDPE_SPP_wrtDN	The ratio of the area under the normalized ABP/PPG cardiac cycle systolic phase peak with respect to diastolic phase peak, with subtracted diastolic phase end point to the area under the normalized ABP/PPG cardiac cycle systolic phase peak with respect to dicrotic notch, with subtracted diastolic phase end point
619	ABP/PPG_D_RT_NAre_wsDPE_SPP_wrtDPE_ABP/PPG_D_NAre_wsDPE_SPP_wrtDN	The ratio of the area under the normalized ABP/PPG cardiac cycle systolic phase peak with respect to diastolic phase end point, with subtracted diastolic phase end point to the area under the normalized ABP/PPG cardiac cycle systolic phase peak with respect to dicrotic notch, with subtracted diastolic phase end point
620	ABP/PPG_D_RT_NAre_wsDPE_DN_wrtDPP_ABP/PPG_D_NAre_wsDPE_SPP_wrtDN	The ratio of the area under the normalized ABP/PPG cardiac cycle dicrotic notch with respect to diastolic phase peak, with subtracted diastolic phase end point to the area under the normalized ABP/PPG cardiac cycle systolic phase peak with respect to dicrotic notch, with subtracted diastolic phase end point
621	ABP/PPG_D_RT_NAre_wsDPE_DN_wrtDPE_ABP/PPG_D_NAre_wsDPE_SPP_wrtDN	The ratio of the area under the normalized ABP/PPG cardiac cycle dicrotic notch with respect to diastolic phase end point, with subtracted diastolic phase end point to the area under the normalized ABP/PPG cardiac cycle systolic phase peak with respect to dicrotic notch, with subtracted diastolic phase end point
622	ABP/PPG_D_RT_NAre_wsDPE_DPP_wrtDPE_ABP/PPG_D_NAre_wsDPE_SPP_wrtDN	The ratio of the area under the normalized ABP/PPG cardiac cycle diastolic phase peak with respect to diastolic phase end point, with subtracted diastolic phase end point to the area under the normalized ABP/PPG cardiac cycle systolic phase peak with respect to dicrotic notch, with subtracted diastolic phase end point
623	ABP/PPG_D_RT_NAre_wsDPE_SPP_wrtDPE_ABP/PPG_D_NAre_wsDPE_SPP_wrtDPP	The ratio of the area under the normalized ABP/PPG cardiac cycle systolic phase peak with respect to diastolic phase end point, with subtracted diastolic phase end point to the area under the normalized ABP/PPG cardiac cycle systolic phase peak with respect to diastolic

		phase peak, with subtracted diastolic phase end point
624	ABP/PPG_D_RT_NAre_wsDPE_DN_wrtDPP_ABP/PPG_D_NAre_wsDPE_SPP_wrtDPP	The ratio of the area under the normalized ABP/PPG cardiac cycle dicrotic notch with respect to diastolic phase peak, with subtracted diastolic phase end point to the area under the normalized ABP/PPG cardiac cycle systolic phase peak with respect to diastolic phase peak, with subtracted diastolic phase end point
625	ABP/PPG_D_RT_NAre_wsDPE_DN_wrtDPE_ABP/PPG_D_NAre_wsDPE_SPP_wrtDPP	The ratio of normalized area of dicrotic notch with respect to diastolic phase end point, with subtracted diastolic phase end point to the area under the normalized ABP/PPG cardiac cycle systolic phase peak with respect to diastolic phase peak, with subtracted diastolic phase end point
626	ABP/PPG_D_RT_NAre_wsDPE_DPP_wrtDPE_ABP/PPG_D_NAre_wsDPE_SPP_wrtDPP	The ratio of the area under the normalized ABP/PPG cardiac cycle diastolic phase peak with respect to diastolic phase end point, with subtracted diastolic phase end point to the area under the normalized ABP/PPG cardiac cycle systolic phase peak with respect to diastolic phase peak, with subtracted diastolic phase end point
627	ABP/PPG_D_RT_NAre_wsDPE_DN_wrtDPP_ABP/PPG_D_NAre_wsDPE_SPP_wrtDPE	The ratio of the area under the normalized ABP/PPG cardiac cycle dicrotic notch with respect to diastolic phase peak, with subtracted diastolic phase end point to the area under the normalized ABP/PPG cardiac cycle systolic phase peak with respect to diastolic phase end point, with subtracted diastolic phase end point
628	ABP/PPG_D_RT_NAre_wsDPE_DN_wrtDPE_ABP/PPG_D_NAre_wsDPE_SPP_wrtDPE	The ratio of the area under the normalized ABP/PPG cardiac cycle dicrotic notch with respect to diastolic phase end point, with subtracted diastolic phase end point to the area under the normalized ABP/PPG cardiac cycle systolic phase peak with respect to diastolic phase end point, with subtracted diastolic phase end point
629	ABP/PPG_D_RT_NAre_wsDPE_DPP_wrtDPE_ABP/PPG_D_NAre_wsDPE_SPP_wrtDPE	The ratio of the area under the normalized ABP/PPG cardiac cycle diastolic phase peak with respect to diastolic phase end point, with subtracted diastolic phase end point to the area under the normalized ABP/PPG cardiac cycle

		systolic phase peak with respect to diastolic phase end point, with subtracted diastolic phase end point
630	ABP/PPG_D_RT_NAre_wsDPE_DN_wrtDPE_ABP/PPG_D_NAre_wsDPE_DN_wrtDPP	The ratio of normalized area of dicrotic notch with respect to diastolic phase end point, with subtracted diastolic phase end point to the area under the normalized ABP/PPG cardiac cycle dicrotic notch with respect to diastolic phase peak, with subtracted diastolic phase end point
631	ABP/PPG_D_RT_NAre_wsDPE_DPP_wrtDPE_ABP/PPG_D_NAre_wsDPE_DN_wrtDPP	The ratio of normalized area of diastolic phase peak with respect to diastolic phase end point, with subtracted diastolic phase end point to the area under the normalized ABP/PPG cardiac cycle dicrotic notch with respect to diastolic phase peak, with subtracted diastolic phase end point
632	ABP/PPG_D_RT_NAre_wsDPE_DPP_wrtDPE_ABP/PPG_D_NAre_wsDPE_DN_wrtDPE	The ratio of the area under the normalized ABP/PPG cardiac cycle diastolic phase peak with respect to diastolic phase end point, with subtracted diastolic phase end point to the area under the normalized ABP/PPG cardiac cycle dicrotic notch with respect to diastolic phase end point, with subtracted diastolic phase end point

18. First derivative features (636-642)

636	ABP/PPG_FD_NO_ZC	ABP/PPG cardiac cycle first derivative number of zero crossings
637	ABP/PPG_AM_FD_Upeak_wrtzero	ABP/PPG cardiac cycle first derivative U peak amplitude with respect to zero
638	ABP/PPG_AM_FD_Vpeak_wrtzero	ABP/PPG cardiac cycle first derivative Vpeak amplitude with respect to zero
639	ABP/PPG_AM_FD_Wpeak_wrtzero	ABP/PPG cardiac cycle first derivative Wpeak amplitude with respect to zero
640	ABP/PPG_AM_FD_Upeak_wrtVpeak	ABP/PPG cardiac cycle first derivative Upeak amplitude with respect to Vpeak

641	ABP/PPG_AM_FD_Upeak_wrtWpeak	ABP/PPG cardiac cycle first derivative Upeak amplitude with respect to Wpeak
642	ABP/PPG_AM_FD_Vpeak_wrtWpeak	ABP/PPG cardiac cycle first derivative Vpeak amplitude with respect to Wpeak

19. First derivative amplitude ratio features (643-657)

643	ABP/PPG_AM_RT_FD_Vpeak_wrtzero_ABP/PPG_AM_FD_Upeak_wrtzero	The ratio of the ABP/PPG cardiac cycle first derivative Vpeak amplitude with respect to zero to the ABP/PPG cardiac cycle first derivative Upeak amplitude with respect to zero
644	ABP/PPG_AM_RT_FD_Wpeak_wrtzero_ABP/PPG_AM_FD_Upeak_wrtzero	The ratio of the ABP/PPG cardiac cycle first derivative Wpeak amplitude with respect to zero to the ABP/PPG cardiac cycle first derivative Upeak amplitude with respect to zero
645	ABP/PPG_AM_RT_FD_Upeak_wrtVpeak_ABP/PPG_AM_FD_Upeak_wrtzero	The ratio of the ABP/PPG cardiac cycle first derivative Upeak amplitude with respect to Vpeak to the ABP/PPG cardiac cycle first derivative Upeak amplitude with respect to zero
646	ABP/PPG_AM_RT_FD_Upeak_wrtWpeak_ABP/PPG_AM_FD_Upeak_wrtzero	The ratio of the ABP/PPG cardiac cycle first derivative Upeak amplitude with respect to Wpeak to the ABP/PPG cardiac cycle first derivative Upeak amplitude with respect to zero
647	ABP/PPG_AM_RT_FD_Vpeak_wrtWpeak_ABP/PPG_AM_FD_Upeak_wrtzero	The ratio of the ABP/PPG cardiac cycle first derivative Vpeak amplitude with respect to Wpeak to the ABP/PPG cardiac cycle first derivative Upeak amplitude with respect to zero
648	ABP/PPG_AM_RT_FD_Wpeak_wrtzero_ABP/PPG_AM_FD_Vpeak_wrtzero	The ratio of the ABP/PPG cardiac cycle first derivative Wpeak amplitude with respect to zero to the ABP/PPG cardiac cycle first derivative Vpeak amplitude with respect to zero
649	ABP/PPG_AM_RT_FD_Upeak_wrtVpeak_ABP/PPG_AM_FD_Vpeak_wrtzero	The ratio of the ABP/PPG cardiac cycle first derivative Upeak amplitude with respect to Vpeak to the ABP/PPG cardiac cycle first derivative Vpeak amplitude with respect to zero

650	ABP/PPG_AM_RT_FD_Upeak_wrtWpeak_ABP/PPG_AM_FD_Vpeak_wrtzero	The ratio of the ABP/PPG cardiac cycle first derivative Upeak amplitude with respect to Wpeak to the ABP/PPG cardiac cycle first derivative Vpeak amplitude with respect to zero
651	ABP/PPG_AM_RT_FD_Vpeak_wrtWpeak_ABP/PPG_AM_FD_Vpeak_wrtzero	The ratio of the ABP/PPG cardiac cycle first derivative Vpeak amplitude with respect to Wpeak to the ABP/PPG cardiac cycle first derivative Vpeak amplitude with respect to zero
652	ABP/PPG_AM_RT_FD_Upeak_wrtVpeak_ABP/PPG_AM_FD_Wpeak_wrtzero	The ratio of the ABP/PPG cardiac cycle first derivative Upeak amplitude with respect to Vpeak to the ABP/PPG cardiac cycle first derivative W peak amplitude with respect to zero
653	ABP/PPG_AM_RT_FD_Upeak_wrtWpeak_ABP/PPG_AM_FD_Wpeak_wrtzero	The ratio of the ABP/PPG cardiac cycle first derivative Upeak amplitude with respect to Wpeak to the ABP/PPG cardiac cycle first derivative Wpeak amplitude with respect to zero
654	ABP/PPG_AM_RT_FD_Vpeak_wrtWpeak_ABP/PPG_AM_FD_Wpeak_wrtzero	The ratio of the ABP/PPG cardiac cycle first derivative Vpeak amplitude with respect to Wpeak to the ABP/PPG cardiac cycle first derivative Wpeak amplitude with respect to zero
655	ABP/PPG_AM_RT_FD_Upeak_wrtWpeak_ABP/PPG_AM_FD_Upeak_wrtVpeak	The ratio of the ABP/PPG cardiac cycle first derivative Upeak amplitude with respect to Wpeak to the ABP/PPG cardiac cycle first derivative Upeak amplitude with respect to Vpeak
656	ABP/PPG_AM_RT_FD_Vpeak_wrtWpeak_ABP/PPG_AM_FD_Upeak_wrtVpeak	The ratio of the ABP/PPG cardiac cycle first derivative Vpeak amplitude with respect to Wpeak to the ABP/PPG cardiac cycle first derivative Upeak amplitude with respect to Vpeak
657	ABP/PPG_AM_RT_FD_Vpeak_wrtWpeak_ABP/PPG_AM_FD_Upeak_wrtWpeak	The ratio of the ABP/PPG cardiac cycle first derivative Vpeak amplitude with respect to Wpeak to the ABP/PPG cardiac cycle first derivative Upeak amplitude with respect to Wpeak

20. First derivative duration features (658-660)

658	ABP/PPG_D_FD_Upeak_wrtVpeak	Duration of the ABP/PPG cardiac cycle first derivative Upeak with respect to Vpeak
659	ABP/PPG_D_FD_Upeak_wrtWpeak	Duration of the ABP/PPG cardiac cycle first derivative Upeak with respect to Wpeak
660	ABP/PPG_D_FD_Vpeak_wrtWpeak	Duration of the ABP/PPG cardiac cycle first derivative Vpeak with respect to Wpeak

21. First derivative duration ratio features (661-663)

661	ABP/PPG_D_RT_FD_Upeak_wrtWpeak_ABP/PPG_D_FD_Upeak_wrtVpeak	The ratio of the ABP/PPG cardiac cycle first derivative Upeak duration with respect to Wpeak to the ABP/PPG cardiac cycle first derivative Upeak duration with respect to Vpeak
662	ABP/PPG_D_RT_FD_Vpeak_wrtWpeak_ABP/PPG_D_FD_Upeak_wrtVpeak	The ratio of the ABP/PPG cardiac cycle first derivative Vpeak duration with respect to Wpeak to the ABP/PPG cardiac cycle first derivative Upeak duration with respect to Vpeak
663	ABP/PPG_D_RT_FD_Vpeak_wrtWpeak_ABP/PPG_D_FD_Upeak_wrtWpeak	The ratio of the ABP/PPG cardiac cycle first derivative Vpeak duration with respect to Wpeak to the ABP/PPG cardiac cycle first derivative Upeak duration with respect to Wpeak

22. Second derivative amplitude features with respect to zero (664-669)

664	ABP/PPG_SD_NO_ZC	ABP/PPG cardiac cycle second derivative number of zero crossings
665	ABP/PPG_AM_SD_Apeak_wrtzero	ABP/PPG cardiac cycle second derivative Apeak amplitude with respect to zero

666	ABP/PPG_AM_SD_Bpeak_wrtzero	ABP/PPG cardiac cycle second derivative Bpeak amplitude with respect to zero
667	ABP/PPG_AM_SD_Cpeak_wrtzero	ABP/PPG cardiac cycle second derivative Cpeak amplitude with respect to zero
668	ABP/PPG_AM_SD_Dpeak_wrtzero	ABP/PPG cardiac cycle second derivative Dpeak amplitude with respect to zero
669	ABP/PPG_AM_SD_Epeak_wrtzero	ABP/PPG cardiac cycle second derivative Epeak amplitude with respect to zero

23. Second derivative amplitude features with respect to peaks (670-680)

670	ABP/PPG_AM_SD_Apeak_wrtBpeak	ABP/PPG cardiac cycle second derivative Apeak amplitude with respect to Bpeak
671	ABP/PPG_AM_SD_Apeak_wrtCpeak	ABP/PPG cardiac cycle second derivative Apeak amplitude with respect to Cpeak
672	ABP/PPG_AM_SD_Apeak_wrtDpeak	ABP/PPG cardiac cycle second derivative Apeak amplitude with respect to Dpeak
673	ABP/PPG_AM_SD_Apeak_wrtEpeak	ABP/PPG cardiac cycle second derivative Apeak amplitude with respect to Epeak
674	ABP/PPG_AM_SD_Bpeak_wrtCpeak	ABP/PPG cardiac cycle second derivative Bpeak amplitude with respect to Cpeak
675	ABP/PPG_AM_SD_Bpeak_wrtDpeak	ABP/PPG cardiac cycle second derivative Bpeak amplitude with respect to Dpeak
676	ABP/PPG_AM_SD_Bpeak_wrtEpeak	ABP/PPG cardiac cycle second derivative Bpeak amplitude with respect to Epeak
678	ABP/PPG_AM_SD_Cpeak_wrtDpeak	ABP/PPG cardiac cycle second derivative Cpeak amplitude with respect to Dpeak
679	ABP/PPG_AM_SD_Cpeak_wrtEpeak	ABP/PPG cardiac cycle second derivative Cpeak amplitude with respect to Epeak

680	ABP/PPG_AM_SD_Dpeak_wrtEpeak	ABP/PPG cardiac cycle second derivative Dpeak amplitude with respect to Epeak

24. Second derivative amplitude ratio features (681-785)

681	ABP/PPG_AM_RT_SD_Bpeak_wrtzero_ABP/PPG_AM_SD_Apeak_wrtzero	The ratio of the ABP/PPG cardiac cycle second derivative Bpeak amplitude with respect to zero to the ABP/PPG cardiac cycle second derivative Apeak amplitude with respect to zero
682	ABP/PPG_AM_RT_SD_Cpeak_wrtzero_ABP/PPG_AM_SD_Apeak_wrtzero	The ratio of the ABP/PPG cardiac cycle second derivative Cpeak amplitude with respect to zero to the ABP/PPG cardiac cycle second derivative Apeak amplitude with respect to zero
683	ABP/PPG_AM_RT_SD_Dpeak_wrtzero_ABP/PPG_AM_SD_Apeak_wrtzero	The ratio of the ABP/PPG cardiac cycle second derivative Dpeak amplitude with respect to zero to the ABP/PPG cardiac cycle second derivative Apeak amplitude with respect to zero
684	ABP/PPG_AM_RT_SD_Epeak_wrtzero_ABP/PPG_AM_SD_Apeak_wrtzero	The ratio of the ABP/PPG cardiac cycle second derivative Epeak amplitude with respect to zero to the ABP/PPG cardiac cycle second derivative Apeak amplitude with respect to zero
685	ABP/PPG_AM_RT_SD_Apeak_wrtBpeak_ABP/PPG_AM_SD_Apeak_wrtzero	The ratio of the ABP/PPG cardiac cycle second derivative Apeak amplitude with respect to Bpeak to the ABP/PPG cardiac cycle second derivative Apeak amplitude with respect to zero
686	ABP/PPG_AM_RT_SD_Apeak_wrtCpeak_ABP/PPG_AM_SD_Apeak_wrtzero	The ratio of the ABP/PPG cardiac cycle second derivative Apeak amplitude with respect to Cpeak to the ABP/PPG cardiac cycle second derivative Apeak amplitude with respect to zero
687	ABP/PPG_AM_RT_SD_Apeak_wrtDpeak_ABP/PPG_AM_SD_Apeak_wrtzero	The ratio of the ABP/PPG cardiac cycle second derivative

		Apeak amplitude with respect to Dpeak to the ABP/PPG cardiac cycle second derivative Apeak amplitude with respect to zero
688	ABP/PPG_AM_RT_SD_Apeak_wrtEpeak_ABP/PPG_AM_SD_Apeak_wrtzero	The ratio of the ABP/PPG cardiac cycle second derivative Apeak amplitude with respect to Epeak to the ABP/PPG cardiac cycle second derivative Apeak amplitude with respect to zero
689	ABP/PPG_AM_RT_SD_Bpeak_wrtCpeak_ABP/PPG_AM_SD_Apeak_wrtzero	The ratio of the ABP/PPG cardiac cycle second derivative Bpeak amplitude with respect to Cpeak to the ABP/PPG cardiac cycle second derivative Apeak amplitude with respect to zero
690	ABP/PPG_AM_RT_SD_Bpeak_wrtDpeak_ABP/PPG_AM_SD_Apeak_wrtzero	The ratio of the ABP/PPG cardiac cycle second derivative Bpeak amplitude with respect to D peak to the ABP/PPG cardiac cycle second derivative Apeak amplitude with respect to zero
691	ABP/PPG_AM_RT_SD_Bpeak_wrtEpeak_ABP/PPG_AM_SD_Apeak_wrtzero	The ratio of the ABP/PPG cardiac cycle second derivative Bpeak amplitude with respect to Epeak to the ABP/PPG cardiac cycle second derivative Apeak amplitude with respect to zero
692	ABP/PPG_AM_RT_SD_Cpeak_wrtDpeak_ABP/PPG_AM_SD_Apeak_wrtzero	The ratio of the ABP/PPG cardiac cycle second derivative Cpeak amplitude with respect to Dpeak to the ABP/PPG cardiac cycle second derivative Apeak amplitude with respect to zero
693	ABP/PPG_AM_RT_SD_Cpeak_wrtEpeak_ABP/PPG_AM_SD_Apeak_wrtzero	The ratio of the ABP/PPG cardiac cycle second derivative Cpeak amplitude with respect to Epeak to the ABP/PPG cardiac cycle second derivative Apeak amplitude with respect to zero
694	ABP/PPG_AM_RT_SD_Dpeak_wrtEpeak_ABP/PPG_AM_SD_Apeak_wrtzero	The ratio of the ABP/PPG cardiac cycle second derivative Dpeak amplitude with respect to Epeak to the ABP/PPG cardiac

		cycle second derivative Apeak amplitude with respect to zero
695	ABP/PPG_AM_RT_SD_Cpeak_wrtzero_ABP/PPG_AM_SD_Bpeak_wrtzero	The ratio of the ABP/PPG cardiac cycle second derivative Cpeak amplitude with respect to zero to the ABP/PPG cardiac cycle second derivative Bpeak amplitude with respect to zero
696	ABP/PPG_AM_RT_SD_Dpeak_wrtzero_ABP/PPG_AM_SD_Bpeak_wrtzero	The ratio of the ABP/PPG cardiac cycle second derivative Dpeak amplitude with respect to zero to the ABP/PPG cardiac cycle second derivative Bpeak amplitude with respect to zero
697	ABP/PPG_AM_RT_SD_Epeak_wrtzero_ABP/PPG_AM_SD_Bpeak_wrtzero	The ratio of the ABP/PPG cardiac cycle second derivative Epeak amplitude with respect to zero to the ABP/PPG cardiac cycle second derivative Bpeak amplitude with respect to zero
698	ABP/PPG_AM_RT_SD_Apeak_wrtBpeak_ABP/PPG_AM_SD_Bpeak_wrtzero	The ratio of the ABP/PPG cardiac cycle second derivative Apeak amplitude with respect to Bpeak to the ABP/PPG cardiac cycle second derivative Bpeak amplitude with respect to zero
699	ABP/PPG_AM_RT_SD_Apeak_wrtCpeak_ABP/PPG_AM_SD_Bpeak_wrtzero	The ratio of the ABP/PPG cardiac cycle second derivative Apeak amplitude with respect to Cpeak to the ABP/PPG cardiac cycle second derivative Bpeak amplitude with respect to zero
700	ABP/PPG_AM_RT_SD_Apeak_wrtDpeak_ABP/PPG_AM_SD_Bpeak_wrtzero	The ratio of the ABP/PPG cardiac cycle second derivative Apeak amplitude with respect to Dpeak to the ABP/PPG cardiac cycle second derivative Bpeak amplitude with respect to zero
701	ABP/PPG_AM_RT_SD_Apeak_wrtEpeak_ABP/PPG_AM_SD_Bpeak_wrtzero	The ratio of the ABP/PPG cardiac cycle second derivative Apeak amplitude with respect to Epeak to The ratio of the ABP/PPG cardiac cycle second

		derivative Bpeak amplitude with respect to zero
702	ABP/PPG_AM_RT_SD_Bpeak_wrtCpeak_ABP/PPG_AM_SD_Bpeak_wrtzero	The ratio of the ABP/PPG cardiac cycle second derivative Bpeak amplitude with respect to Cpeak to the ABP/PPG cardiac cycle second derivative Bpeak amplitude with respect to zero
703	ABP/PPG_AM_RT_SD_Bpeak_wrtDpeak_ABP/PPG_AM_SD_Bpeak_wrtzero	The ratio of the ABP/PPG cardiac cycle second derivative Bpeak amplitude with respect to Dpeak to the ABP/PPG cardiac cycle second derivative Bpeak amplitude with respect to zero
704	ABP/PPG_AM_RT_SD_Bpeak_wrtEpeak_ABP/PPG_AM_SD_Bpeak_wrtzero	The ratio of the ABP/PPG cardiac cycle second derivative Bpeak amplitude with respect to Epeak to the ABP/PPG cardiac cycle second derivative Bpeak amplitude with respect to zero
705	ABP/PPG_AM_RT_SD_Cpeak_wrtDpeak_ABP/PPG_AM_SD_Bpeak_wrtzero	The ratio of the ABP/PPG cardiac cycle second derivative Cpeak amplitude with respect to Dpeak to the ABP/PPG cardiac cycle second derivative Bpeak amplitude with respect to zero
706	ABP/PPG_AM_RT_SD_Cpeak_wrtEpeak_ABP/PPG_AM_SD_Bpeak_wrtzero	The ratio of the ABP/PPG cardiac cycle second derivative Cpeak amplitude with respect to Epeak to the ABP/PPG cardiac cycle second derivative Bpeak amplitude with respect to zero
707	ABP/PPG_AM_RT_SD_Dpeak_wrtEpeak_ABP/PPG_AM_SD_Bpeak_wrtzero	The ratio of the ABP/PPG cardiac cycle second derivative Dpeak amplitude with respect to Epeak to the ABP/PPG cardiac cycle second derivative Bpeak amplitude with respect to zero
708	ABP/PPG_AM_RT_SD_Dpeak_wrtzero_ABP/PPG_AM_SD_Cpeak_wrtzero	The ratio of the ABP/PPG cardiac cycle second derivative Dpeak amplitude with respect to zero to the ABP/PPG cardiac cycle second derivative Cpeak amplitude with respect to zero

709	ABP/PPG_AM_RT_SD_Epeak_wrtzero_ABP/PPG_AM_SD_Cpeak_wrtzero	The ratio of the ABP/PPG cardiac cycle second derivative Epeak amplitude with respect to zero to the ABP/PPG cardiac cycle second derivative Cpeak amplitude with respect to zero
710	ABP/PPG_AM_RT_SD_Apeak_wrtBpeak_ABP/PPG_AM_SD_Cpeak_wrtzero	The ratio of the ABP/PPG cardiac cycle second derivative Apeak amplitude with respect to Bpeak to the ABP/PPG cardiac cycle second derivative Cpeak amplitude with respect to zero
711	ABP/PPG_AM_RT_SD_Apeak_wrtCpeak_ABP/PPG_AM_SD_Cpeak_wrtzero	The ratio of the ABP/PPG cardiac cycle second derivative Apeak amplitude with respect to Cpeak to the ABP/PPG cardiac cycle second derivative Cpeak amplitude with respect to zero
712	ABP/PPG_AM_RT_SD_Apeak_wrtDpeak_ABP/PPG_AM_SD_Cpeak_wrtzero	The ratio of the ABP/PPG cardiac cycle second derivative Apeak amplitude with respect to Dpeak to the ABP/PPG cardiac cycle second derivative Cpeak amplitude with respect to zero
713	ABP/PPG_AM_RT_SD_Apeak_wrtEpeak_ABP/PPG_AM_SD_Cpeak_wrtzero	The ratio of the ABP/PPG cardiac cycle second derivative Apeak amplitude with respect to Epeak to the ABP/PPG cardiac cycle second derivative Cpeak amplitude with respect to zero
714	ABP/PPG_AM_RT_SD_Bpeak_wrtCpeak_ABP/PPG_AM_SD_Cpeak_wrtzero	The ratio of the ABP/PPG cardiac cycle second derivative Bpeak amplitude with respect to Cpeak to the ABP/PPG cardiac cycle second derivative Cpeak amplitude with respect to zero
715	ABP/PPG_AM_RT_SD_Bpeak_wrtDpeak_ABP/PPG_AM_SD_Cpeak_wrtzero	The ratio of the ABP/PPG cardiac cycle second derivative Bpeak amplitude with respect to Dpeak to the ABP/PPG cardiac cycle second derivative Cpeak amplitude with respect to zero
716	ABP/PPG_AM_RT_SD_Bpeak_wrtEpeak_ABP/PPG_AM_SD_Cpeak_wrtzero	The ratio of the ABP/PPG cardiac cycle second derivative

		Bpeak amplitude with respect to Epeak to the ABP/PPG cardiac cycle second derivative Cpeak amplitude with respect to zero
717	ABP/PPG_AM_RT_SD_Cpeak_wrtDpeak_ABP/PPG_AM_SD_Cpeak_wrtzero	The ratio of the ABP/PPG cardiac cycle second derivative Cpeak amplitude with respect to D peak to the ABP/PPG cardiac cycle second derivative Cpeak amplitude with respect to zero
718	ABP/PPG_AM_RT_SD_Cpeak_wrtEpeak_ABP/PPG_AM_SD_Cpeak_wrtzero	The ratio of the ABP/PPG cardiac cycle second derivative Cpeak amplitude with respect to Epeak to the ABP/PPG cardiac cycle second derivative Cpeak amplitude with respect to zero
719	ABP/PPG_AM_RT_SD_Dpeak_wrtEpeak_ABP/PPG_AM_SD_Cpeak_wrtzero	The ratio of the ABP/PPG cardiac cycle second derivative Dpeak amplitude with respect to Epeak to the ABP/PPG cardiac cycle second derivative Cpeak amplitude with respect to zero
720	ABP/PPG_AM_RT_SD_Epeak_wrtzero_ABP/PPG_AM_SD_Dpeak_wrtzero	The ratio of the ABP/PPG cardiac cycle second derivative Epeak amplitude with respect to zero to the ABP/PPG cardiac cycle second derivative Dpeak amplitude with respect to zero
721	ABP/PPG_AM_RT_SD_Apeak_wrtBpeak_ABP/PPG_AM_SD_Dpeak_wrtzero	The ratio of the ABP/PPG cardiac cycle second derivative Apeak amplitude with respect to Bpeak to the ABP/PPG cardiac cycle second derivative Dpeak amplitude with respect to zero
722	ABP/PPG_AM_RT_SD_Apeak_wrtCpeak_ABP/PPG_AM_SD_Dpeak_wrtzero	The ratio of the ABP/PPG cardiac cycle second derivative Apeak amplitude with respect to Cpeak to the ABP/PPG cardiac cycle second derivative Dpeak amplitude with respect to zero
723	ABP/PPG_AM_RT_SD_Apeak_wrtDpeak_ABP/PPG_AM_SD_Dpeak_wrtzero	The ratio of the ABP/PPG cardiac cycle second derivative Apeak amplitude with respect to Dpeak to the ABP/PPG cardiac

		cycle second derivative Dpeak amplitude with respect to zero
724	ABP/PPG_AM_RT_SD_Apeak_wrtEpeak_ABP/PPG_AM_SD_Dpeak_wrtzero	The ratio of the ABP/PPG cardiac cycle second derivative Apeak amplitude with respect to Epeak to the ABP/PPG cardiac cycle second derivative Dpeak amplitude with respect to zero
725	ABP/PPG_AM_RT_SD_Bpeak_wrtCpeak_ABP/PPG_AM_SD_Dpeak_wrtzero	The ratio of the ABP/PPG cardiac cycle second derivative Bpeak amplitude with respect to Cpeak to the ABP/PPG cardiac cycle second derivative Dpeak amplitude with respect to zero
726	ABP/PPG_AM_RT_SD_Bpeak_wrtDpeak_ABP/PPG_AM_SD_Dpeak_wrtzero	The ratio of the ABP/PPG cardiac cycle second derivative Bpeak amplitude with respect to Dpeak to the ABP/PPG cardiac cycle second derivative Dpeak amplitude with respect to zero
727	ABP/PPG_AM_RT_SD_Bpeak_wrtEpeak_ABP/PPG_AM_SD_Dpeak_wrtzero	The ratio of the ABP/PPG cardiac cycle second derivative Bpeak amplitude with respect to Epeak to the ABP/PPG cardiac cycle second derivative Dpeak amplitude with respect to zero
728	ABP/PPG_AM_RT_SD_Cpeak_wrtDpeak_ABP/PPG_AM_SD_Dpeak_wrtzero	The ratio of the ABP/PPG cardiac cycle second derivative Cpeak amplitude with respect to Dpeak to the ABP/PPG cardiac cycle second derivative Dpeak amplitude with respect to zero
729	ABP/PPG_AM_RT_SD_Cpeak_wrtEpeak_ABP/PPG_AM_SD_Dpeak_wrtzero	The ratio of the ABP/PPG cardiac cycle second derivative Cpeak amplitude with respect to Epeak to the ABP/PPG cardiac cycle second derivative Dpeak amplitude with respect to zero
730	ABP/PPG_AM_RT_SD_Dpeak_wrtEpeak_ABP/PPG_AM_SD_Dpeak_wrtzero	The ratio of the ABP/PPG cardiac cycle second derivative Dpeak amplitude with respect to Epeak to the ABP/PPG cardiac cycle second derivative Dpeak amplitude with respect to zero

731	ABP/PPG_AM_RT_SD_Apeak_wrtBpeak_ABP/PPG_AM_SD_Epeak_wrtzero	The ratio of the ABP/PPG cardiac cycle second derivative Apeak amplitude with respect to Bpeak to the ABP/PPG cardiac cycle second derivative Epeak amplitude with respect to zero
732	ABP/PPG_AM_RT_SD_Apeak_wrtCpeak_ABP/PPG_AM_SD_Epeak_wrtzero	The ratio of the ABP/PPG cardiac cycle second derivative Apeak amplitude with respect to Cpeak to the ABP/PPG cardiac cycle second derivative Epeak amplitude with respect to zero
733	ABP/PPG_AM_RT_SD_Apeak_wrtDpeak_ABP/PPG_AM_SD_Epeak_wrtzero	The ratio of the ABP/PPG cardiac cycle second derivative Apeak amplitude with respect to Dpeak to the ABP/PPG cardiac cycle second derivative Epeak amplitude with respect to zero
734	ABP/PPG_AM_RT_SD_Apeak_wrtEpeak_ABP/PPG_AM_SD_Epeak_wrtzero	The ratio of the ABP/PPG cardiac cycle second derivative Apeak amplitude with respect to Epeak to the ABP/PPG cardiac cycle second derivative Epeak amplitude with respect to zero
735	ABP/PPG_AM_RT_SD_Bpeak_wrtCpeak_ABP/PPG_AM_SD_Epeak_wrtzero	The ratio of the ABP/PPG cardiac cycle second derivative Bpeak amplitude with respect to Cpeak to the ABP/PPG cardiac cycle second derivative Epeak amplitude with respect to zero
736	ABP/PPG_AM_RT_SD_Bpeak_wrtDpeak_ABP/PPG_AM_SD_Epeak_wrtzero	The ratio of the ABP/PPG cardiac cycle second derivative Bpeak amplitude with respect to Dpeak to second derviative of PPG cardiac cycle Epeak amplitude with respect to zero
737	ABP/PPG_AM_RT_SD_Bpeak_wrtEpeak_ABP/PPG_AM_SD_Epeak_wrtzero	The ratio of the ABP/PPG cardiac cycle second derivative Bpeak amplitude with respect to Epeak to the ABP/PPG cardiac cycle second derivative Epeak amplitude with respect to zero
738	ABP/PPG_AM_RT_SD_Cpeak_wrtDpeak_ABP/PPG_AM_SD_Epeak_wrtzero	The ratio of the ABP/PPG cardiac cycle second derivative

		Cpeak amplitude with respect to Dpeak to the ABP/PPG cardiac cycle second derivative Epeak amplitude with respect to zero
739	ABP/PPG_AM_RT_SD_Cpeak_wrtEpeak_ABP/PPG_AM_SD_Epeak_wrtzero	The ratio of the ABP/PPG cardiac cycle second derivative Cpeak amplitude with respect to Epeak to the ABP/PPG cardiac cycle second derivative Epeak amplitude with respect to zero
740	ABP/PPG_AM_RT_SD_Dpeak_wrtEpeak_ABP/PPG_AM_SD_Epeak_wrtzero	The ratio of the ABP/PPG cardiac cycle second derivative Dpeak amplitude with respect to Epeak to the ABP/PPG cardiac cycle second derivative Epeak amplitude with respect to zero
741	ABP/PPG_AM_RT_SD_Apeak_wrtCpeak_ABP/PPG_AM_SD_Apeak_wrtBpeak	The ratio of the ABP/PPG cardiac cycle second derivative Apeak amplitude with respect to Cpeak to the ABP/PPG cardiac cycle second derivative Apeak amplitude with respect to Bpeak
742	ABP/PPG_AM_RT_SD_Apeak_wrtDpeak_ABP/PPG_AM_SD_Apeak_wrtBpeak	The ratio of the ABP/PPG cardiac cycle second derivative Apeak amplitude with respect to Dpeak to the ABP/PPG cardiac cycle second derivative Apeak amplitude with respect to Bpeak
743	ABP/PPG_AM_RT_SD_Apeak_wrtEpeak_ABP/PPG_AM_SD_Apeak_wrtBpeak	The ratio of the ABP/PPG cardiac cycle second derivative Apeak amplitude with respect to Epeak to the ABP/PPG cardiac cycle second derivative Apeak amplitude with respect to Bpeak
744	ABP/PPG_AM_RT_SD_Bpeak_wrtCpeak_ABP/PPG_AM_SD_Apeak_wrtBpeak	The ratio of the ABP/PPG cardiac cycle second derivative Bpeak amplitude with respect to Cpeak to the ABP/PPG cardiac cycle second derivative Apeak amplitude with respect to Bpeak
745	ABP/PPG_AM_RT_SD_Bpeak_wrtDpeak_ABP/PPG_AM_SD_Apeak_wrtBpeak	The ratio of the ABP/PPG cardiac cycle second derivative Bpeak amplitude with respect to Dpeak to the ABP/PPG cardiac

		cycle second derivative Apeak amplitude with respect to Bpeak
746	ABP/PPG_AM_RT_SD_Bpeak_wrtEpeak_ABP/PPG_AM_SD_Apeak_wrtBpeak	The ratio of the ABP/PPG cardiac cycle second derivative Bpeak amplitude with respect to Epeak to the ABP/PPG cardiac cycle second derivative Apeak amplitude with respect to Bpeak
747	ABP/PPG_AM_RT_SD_Cpeak_wrtDpeak_ABP/PPG_AM_SD_Apeak_wrtBpeak	The ratio of the ABP/PPG cardiac cycle second derivative Cpeak amplitude with respect to Dpeak to the ABP/PPG cardiac cycle second derivative Apeak amplitude with respect to Bpeak
748	ABP/PPG_AM_RT_SD_Cpeak_wrtEpeak_ABP/PPG_AM_SD_Apeak_wrtBpeak	The ratio of the ABP/PPG cardiac cycle second derivative Cpeak amplitude with respect to Epeak to the ABP/PPG cardiac cycle second derivative Apeak amplitude with respect to Bpeak
749	ABP/PPG_AM_RT_SD_Dpeak_wrtEpeak_ABP/PPG_AM_SD_Apeak_wrtBpeak	The ratio of the ABP/PPG cardiac cycle second derivative Dpeak amplitude with respect to Epeak to the ABP/PPG cardiac cycle second derivative Apeak amplitude with respect to Bpeak
750	ABP/PPG_AM_RT_SD_Apeak_wrtDpeak_ABP/PPG_AM_SD_Apeak_wrtCpeak	The ratio of the ABP/PPG cardiac cycle second derivative Apeak amplitude with respect to Dpeak to the ABP/PPG cardiac cycle second derivative Apeak amplitude with respect to Cpeak
751	ABP/PPG_AM_RT_SD_Apeak_wrtEpeak_ABP/PPG_AM_SD_Apeak_wrtCpeak	The ratio of the ABP/PPG cardiac cycle second derivative Apeak amplitude with respect to Epeak to the ABP/PPG cardiac cycle second derivative Apeak amplitude with respect to Cpeak
752	ABP/PPG_AM_RT_SD_Bpeak_wrtCpeak_ABP/PPG_AM_SD_Apeak_wrtCpeak	The ratio of the ABP/PPG cardiac cycle second derivative Bpeak amplitude with respect to Cpeak to the ABP/PPG cardiac cycle second derivative Apeak amplitude with respect to Cpeak

753	ABP/PPG_AM_RT_SD_Bpeak_wrtDpeak_ABP/PPG_AM_SD_Apeak_wrtCpeak	The ratio of the ABP/PPG cardiac cycle second derivative Bpeak amplitude with respect to Dpeak to the ABP/PPG cardiac cycle second derivative Apeak amplitude with respect to Cpeak
754	ABP/PPG_AM_RT_SD_Bpeak_wrtEpeak_ABP/PPG_AM_SD_Apeak_wrtCpeak	The ratio of the ABP/PPG cardiac cycle second derivative Bpeak amplitude with respect to Epeak to the ABP/PPG cardiac cycle second derivative Apeak amplitude with respect to Cpeak
755	ABP/PPG_AM_RT_SD_Cpeak_wrtDpeak_ABP/PPG_AM_SD_Apeak_wrtCpeak	The ratio of the ABP/PPG cardiac cycle second derivative Cpeak amplitude with respect to Dpeak to the ABP/PPG cardiac cycle second derivative Apeak amplitude with respect to Cpeak
756	ABP/PPG_AM_RT_SD_Cpeak_wrtEpeak_ABP/PPG_AM_SD_Apeak_wrtCpeak	The ratio of the ABP/PPG cardiac cycle second derivative Cpeak amplitude with respect to Epeak to the ABP/PPG cardiac cycle second derivative Apeak amplitude with respect to Cpeak
757	ABP/PPG_AM_RT_SD_Dpeak_wrtEpeak_ABP/PPG_AM_SD_Apeak_wrtCpeak	The ratio of the ABP/PPG cardiac cycle second derivative Dpeak amplitude with respect to Epeak to the ABP/PPG cardiac cycle second derivative Apeak amplitude with respect to Cpeak
758	ABP/PPG_AM_RT_SD_Apeak_wrtEpeak_ABP/PPG_AM_SD_Apeak_wrtDpeak	The ratio of the ABP/PPG cardiac cycle second derivative Apeak amplitude with respect to Epeak to the ABP/PPG cardiac cycle second derivative Apeak amplitude with respect to Dpeak
759	ABP/PPG_AM_RT_SD_Bpeak_wrtCpeak_ABP/PPG_AM_SD_Apeak_wrtDpeak	The ratio of the ABP/PPG cardiac cycle second derivative Bpeak amplitude with respect to Cpeak to the ABP/PPG cardiac cycle second derivative Apeak amplitude with respect to Dpeak

760	ABP/PPG_AM_RT_SD_Bpeak_wrtDpeak_ABP/PPG_AM_SD_Apeak_wrtDpeak	The ratio of the ABP/PPG cardiac cycle second derivative Bpeak amplitude with respect to Dpeak to the ABP/PPG cardiac cycle second derivative Apeak amplitude with respect to Dpeak
761	ABP/PPG_AM_RT_SD_Bpeak_wrtEpeak_ABP/PPG_AM_SD_Apeak_wrtDpeak	The ratio of the ABP/PPG cardiac cycle second derivative Bpeak amplitude with respect to Epeak to the ABP/PPG cardiac cycle second derivative Apeak amplitude with respect to Dpeak
762	ABP/PPG_AM_RT_SD_Cpeak_wrtDpeak_ABP/PPG_AM_SD_Apeak_wrtDpeak	The ratio of the ABP/PPG cardiac cycle second derivative Cpeak amplitude with respect to Dpeak to the ABP/PPG cardiac cycle second derivative Apeak amplitude with respect to Dpeak
763	ABP/PPG_AM_RT_SD_Cpeak_wrtEpeak_ABP/PPG_AM_SD_Apeak_wrtDpeak	The ratio of the ABP/PPG cardiac cycle second derivative Cpeak amplitude with respect to Epeak to the ABP/PPG cardiac cycle second derivative Apeak amplitude with respect to Dpeak
764	ABP/PPG_AM_RT_SD_Dpeak_wrtEpeak_ABP/PPG_AM_SD_Apeak_wrtDpeak	The ratio of the ABP/PPG cardiac cycle second derivative Dpeak amplitude with respect to Epeak to the ABP/PPG cardiac cycle second derivative Apeak amplitude with respect to Dpeak
765	ABP/PPG_AM_RT_SD_Bpeak_wrtCpeak_ABP/PPG_AM_SD_Apeak_wrtEpeak	The ratio of the ABP/PPG cardiac cycle second derivative Bpeak amplitude with respect to Cpeak to the ABP/PPG cardiac cycle second derivative Apeak amplitude with respect to Epeak
766	ABP/PPG_AM_RT_SD_Bpeak_wrtDpeak_ABP/PPG_AM_SD_Apeak_wrtEpeak	The ratio of the ABP/PPG cardiac cycle second derivative Bpeak amplitude with respect to

		Dpeak to the ABP/PPG cardiac cycle second derivative Apeak amplitude with respect to Epeak
767	ABP/PPG_AM_RT_SD_Bpeak_wrtEpeak_ABP/PPG_AM_SD_Apeak_wrtEpeak	The ratio of the ABP/PPG cardiac cycle second derivative Bpeak amplitude with respect to Epeak to the ABP/PPG cardiac cycle second derivative Apeak amplitude with respect to Epeak
768	ABP/PPG_AM_RT_SD_Cpeak_wrtDpeak_ABP/PPG_AM_SD_Apeak_wrtEpeak	The ratio of the ABP/PPG cardiac cycle second derivative Cpeak amplitude with respect to Dpeak to the ABP/PPG cardiac cycle second derivative Apeak amplitude with respect to Epeak
769	ABP/PPG_AM_RT_SD_Cpeak_wrtEpeak_ABP/PPG_AM_SD_Apeak_wrtEpeak	The ratio of the ABP/PPG cardiac cycle second derivative Cpeak amplitude with respect to Epeak to the ABP/PPG cardiac cycle second derivative Apeak amplitude with respect to Epeak
770	ABP/PPG_AM_RT_SD_Dpeak_wrtEpeak_ABP/PPG_AM_SD_Apeak_wrtEpeak	The ratio of the ABP/PPG cardiac cycle second derivative Dpeak amplitude with respect to Epeak to the ABP/PPG cardiac cycle second derivative Apeak amplitude with respect to Epeak
771	ABP/PPG_AM_RT_SD_Bpeak_wrtDpeak_ABP/PPG_AM_SD_Bpeak_wrtCpeak	The ratio of the ABP/PPG cardiac cycle second derivative Bpeak amplitude with respect to Dpeak to the ABP/PPG cardiac cycle second derivative Bpeak amplitude with respect to Cpeak
772	ABP/PPG_AM_RT_SD_Bpeak_wrtEpeak_ABP/PPG_AM_SD_Bpeak_wrtCpeak	The ratio of the ABP/PPG cardiac cycle second derivative Bpeak amplitude with respect to Epeak to the ABP/PPG cardiac cycle second derivative Bpeak amplitude with respect to Cpeak
773	ABP/PPG_AM_RT_SD_Cpeak_wrtDpeak_ABP/PPG_AM_SD_Bpeak_wrtCpeak	The ratio of the ABP/PPG cardiac cycle second derivative Cpeak amplitude with respect to Dpeak to the ABP/PPG cardiac

		cycle second derivative Bpeak amplitude with respect to Cpeak
774	ABP/PPG_AM_RT_SD_Cpeak_wrtEpeak_ABP/PPG_AM_SD_Bpeak_wrtCpeak	The ratio of the ABP/PPG cardiac cycle second derivative Cpeak amplitude with respect to Epeak to the ABP/PPG cardiac cycle second derivative Bpeak amplitude with respect to Cpeak
775	ABP/PPG_AM_RT_SD_Dpeak_wrtEpeak_ABP/PPG_AM_SD_Bpeak_wrtCpeak	The ratio of the ABP/PPG cardiac cycle second derivative Dpeak amplitude with respect to Epeak to the ABP/PPG cardiac cycle second derivative Bpeak amplitude with respect to Cpeak
776	ABP/PPG_AM_RT_SD_Bpeak_wrtEpeak_ABP/PPG_AM_SD_Bpeak_wrtDpeak	The ratio of the ABP/PPG cardiac cycle second derivative Bpeak amplitude with respect to Epeak to the ABP/PPG cardiac cycle second derivative Bpeak amplitude with respect to Dpeak
777	ABP/PPG_AM_RT_SD_Cpeak_wrtDpeak_ABP/PPG_AM_SD_Bpeak_wrtDpeak	The ratio of the ABP/PPG cardiac cycle second derivative Cpeak amplitude with respect to Dpeak to the ABP/PPG cardiac cycle second derivative Bpeak amplitude with respect to Dpeak
778	ABP/PPG_AM_RT_SD_Cpeak_wrtEpeak_ABP/PPG_AM_SD_Bpeak_wrtDpeak	The ratio of the ABP/PPG cardiac cycle second derivative Cpeak amplitude with respect to Epeak to the ABP/PPG cardiac cycle second derivative Bpeak amplitude with respect to Dpeak
779	ABP/PPG_AM_RT_SD_Dpeak_wrtEpeak_ABP/PPG_AM_SD_Bpeak_wrtDpeak	The ratio of the ABP/PPG cardiac cycle second derivative Dpeak amplitude with respect to Epeak to the ABP/PPG cardiac cycle second derivative Bpeak amplitude with respect to Dpeak
780	ABP/PPG_AM_RT_SD_Cpeak_wrtDpeak_ABP/PPG_AM_SD_Bpeak_wrtEpeak	The ratio of the ABP/PPG cardiac cycle second derivative

		Cpeak amplitude with respect to Dpeak to the ABP/PPG cardiac cycle second derivative Bpeak amplitude with respect to Epeak
781	ABP/PPG_AM_RT_SD_Cpeak_wrtEpeak_ABP/PPG_AM_SD_Bpeak_wrtEpeak	The ratio of the ABP/PPG cardiac cycle second derivative Cpeak amplitude with respect to Epeak to the ABP/PPG cardiac cycle second derivative Bpeak amplitude with respect to Epeak
782	ABP/PPG_AM_RT_SD_Dpeak_wrtEpeak_ABP/PPG_AM_SD_Bpeak_wrtEpeak	The ratio of the ABP/PPG cardiac cycle second derivative Dpeak amplitude with respect to Epeak to the ABP/PPG cardiac cycle second derivative Bpeak amplitude with respect to Epeak
783	ABP/PPG_AM_RT_SD_Cpeak_wrtEpeak_ABP/PPG_AM_SD_Cpeak_wrtDpeak	The ratio of the ABP/PPG cardiac cycle second derivative Cpeak amplitude with respect to Epeak to the ABP/PPG cardiac cycle second derivative Cpeak amplitude with respect to Dpeak
784	ABP/PPG_AM_RT_SD_Dpeak_wrtEpeak_ABP/PPG_AM_SD_Cpeak_wrtDpeak	The ratio of the ABP/PPG cardiac cycle second derivative Dpeak amplitude with respect to Epeak to the ABP/PPG cardiac cycle second derivative Cpeak amplitude with respect to Dpeak
785	ABP/PPG_AM_RT_SD_Dpeak_wrtEpeak_ABP/PPG_AM_SD_Cpeak_wrtEpeak	The ratio of the ABP/PPG cardiac cycle second derivative Dpeak amplitude with respect to Epeak to the ABP/PPG cardiac cycle second derivative Cpeak amplitude with respect to Epeak

25. Second derivative duration features (786-795)

786	ABP/PPG_D_SD_Apeak_wrtBpeak	Duration of the ABP/PPG cardiac cycle second derivative Apeak duration with respect to Bpeak

787	ABP/PPG_D_SD_Apeak_wrtCpeak	Duration of the ABP/PPG cardiac cycle second derivative Apeak duration with respect to Cpeak
788	ABP/PPG_D_SD_Apeak_wrtDpeak	Duration of the ABP/PPG cardiac cycle second derivative Apeak duration with respect to Dpeak
789	ABP/PPG_D_SD_Apeak_wrtEpeak	Duration of the ABP/PPG cardiac cycle second derivative Apeak duration with respect to Epeak
790	ABP/PPG_D_SD_Bpeak_wrtCpeak	Duration of the ABP/PPG cardiac cycle second derivative Bpeak duration with respect to Cpeak
791	ABP/PPG_D_SD_Bpeak_wrtDpeak	Duration of the ABP/PPG cardiac cycle second derivative Bpeak duration with respect to Dpeak
792	ABP/PPG_D_SD_Bpeak_wrtEpeak	Duration of the ABP/PPG cardiac cycle second derivative Bpeak duration with respect to Epeak
793	ABP/PPG_D_SD_Cpeak_wrtDpeak	Duration of the ABP/PPG cardiac cycle second derivative Cpeak duration with respect to Dpeak
794	ABP/PPG_D_SD_Cpeak_wrtEpeak	Duration of the ABP/PPG cardiac cycle second derivative Cpeak duration with respect to Epeak
795	ABP/PPG_D_SD_Dpeak_wrtEpeak	Duration of the ABP/PPG cardiac cycle second derivative Dpeak duration with respect to Epeak

26. Second derivative duration ratio features (796-840)

796	ABP/PPG_D_RT_SD_Apeak_wrtCpeak_ABP/PPG_D_SD_Apeak_wrtBpeak	The ratio of the ABP/PPG cardiac cycle second derivative Apeak duration with respect to Cpeak to the ABP/PPG cardiac cycle second derivative Apeak duration with respect to Bpeak
797	ABP/PPG_D_RT_SD_Apeak_wrtDpeak_ABP/PPG_D_SD_Apeak_wrtBpeak	The ratio of the ABP/PPG cardiac cycle second derivative Apeak duration with respect to Dpeak to the ABP/PPG cardiac cycle second derivative Apeak duration with respect to Bpeak
798	ABP/PPG_D_RT_SD_Apeak_wrtEpeak_ABP/PPG_D_SD_Apeak_wrtBpeak	The ratio of the ABP/PPG cardiac cycle second derivative

		Apeak duration with respect to Epeak to the ABP/PPG cardiac cycle second derivative Apeak duration with respect to Bpeak
799	ABP/PPG_D_RT_SD_Bpeak_wrtCpeak_ABP/PPG_D_SD_Apeak_wrtBpeak	The ratio of the ABP/PPG cardiac cycle second derivative Bpeak duration with respect to Cpeak to the ABP/PPG cardiac cycle second derivative Apeak duration with respect to Bpeak
800	ABP/PPG_D_RT_SD_Bpeak_wrtDpeak_ABP/PPG_D_SD_Apeak_wrtBpeak	The ratio of the ABP/PPG cardiac cycle second derivative Bpeak duration with respect to Dpeak to the ABP/PPG cardiac cycle second derivative Apeak duration with respect to Bpeak
801	ABP/PPG_D_RT_SD_Bpeak_wrtEpeak_ABP/PPG_D_SD_Apeak_wrtBpeak	The ratio of the ABP/PPG cardiac cycle second derivative Bpeak duration with respect to Epeak to the ABP/PPG cardiac cycle second derivative Apeak duration with respect to Bpeak
802	ABP/PPG_D_RT_SD_Cpeak_wrtDpeak_ABP/PPG_D_SD_Apeak_wrtBpeak	The ratio of the ABP/PPG cardiac cycle second derivative Cpeak duration with respect to Dpeak to the ABP/PPG cardiac cycle second derivative Apeak duration with respect to Bpeak
803	ABP/PPG_D_RT_SD_Cpeak_wrtEpeak_ABP/PPG_D_SD_Apeak_wrtBpeak	The ratio of the ABP/PPG cardiac cycle second derivative Cpeak duration with respect to Epeak to the ABP/PPG cardiac cycle second derivative Apeak duration with respect to Bpeak
804	ABP/PPG_D_RT_SD_Dpeak_wrtEpeak_ABP/PPG_D_SD_Apeak_wrtBpeak	The ratio of the ABP/PPG cardiac cycle second derivative Dpeak duration with respect to Epeak to the ABP/PPG cardiac cycle second derivative Apeak duration with respect to Bpeak
805	ABP/PPG_D_RT_SD_Apeak_wrtDpeak_ABP/PPG_D_SD_Apeak_wrtCpeak	The ratio of the ABP/PPG cardiac cycle second derivative Apeak duration with respect to Dpeak to the ABP/PPG cardiac

		cycle second derivative Apeak duration with respect to Cpeak
806	ABP/PPG_D_RT_SD_Apeak_wrtEpeak_ABP/PPG_D_SD_Apeak_wrtCpeak	The ratio of the ABP/PPG cardiac cycle second derivative Apeak duration with respect to Epeak to the ABP/PPG cardiac cycle second derivative Apeak duration with respect to Cpeak
807	ABP/PPG_D_RT_SD_Bpeak_wrtCpeak_ABP/PPG_D_SD_Apeak_wrtCpeak	The ratio of the ABP/PPG cardiac cycle second derivative Bpeak duration with respect to Cpeak to the ABP/PPG cardiac cycle second derivative Apeak duration with respect to Cpeak
808	ABP/PPG_D_RT_SD_Bpeak_wrtDpeak_ABP/PPG_D_SD_Apeak_wrtCpeak	The ratio of the ABP/PPG cardiac cycle second derivative Bpeak duration with respect to Dpeak to the ABP/PPG cardiac cycle second derivative Apeak duration with respect to Cpeak
809	ABP/PPG_D_RT_SD_Bpeak_wrtEpeak_ABP/PPG_D_SD_Apeak_wrtCpeak	The ratio of the ABP/PPG cardiac cycle second derivative Bpeak duration with respect to Epeak to the ABP/PPG cardiac cycle second derivative Apeak duration with respect to Cpeak
810	ABP/PPG_D_RT_SD_Cpeak_wrtDpeak_ABP/PPG_D_SD_Apeak_wrtCpeak	The ratio of the ABP/PPG cardiac cycle second derivative Cpeak duration with respect to Dpeak to the ABP/PPG cardiac cycle second derivative Apeak duration with respect to Cpeak
811	ABP/PPG_D_RT_SD_Cpeak_wrtEpeak_ABP/PPG_D_SD_Apeak_wrtCpeak	The ratio of the ABP/PPG cardiac cycle second derivative Cpeak duration with respect to Epeak to the ABP/PPG cardiac cycle second derivative Apeak duration with respect to Cpeak
812	ABP/PPG_D_RT_SD_Dpeak_wrtEpeak_ABP/PPG_D_SD_Apeak_wrtCpeak	The ratio of the ABP/PPG cardiac cycle second derivative Dpeak duration with respect to Epeak to the ABP/PPG cardiac cycle second derivative Apeak duration with respect to Cpeak

813	ABP/PPG_D_RT_SD_Apeak_wrtEpeak_ABP/PPG_D_SD_Apeak_wrtDpeak	The ratio of the ABP/PPG cardiac cycle second derivative Apeak duration with respect to Epeak to the ABP/PPG cardiac cycle second derivative Apeak duration with respect to Dpeak
814	ABP/PPG_D_RT_SD_Bpeak_wrtCpeak_ABP/PPG_D_SD_Apeak_wrtDpeak	The ratio of the ABP/PPG cardiac cycle second derivative Bpeak duration with respect to Cpeak to the ABP/PPG cardiac cycle second derivative Apeak duration with respect to Dpeak
815	ABP/PPG_D_RT_SD_Bpeak_wrtDpeak_ABP/PPG_D_SD_Apeak_wrtDpeak	The ratio of the ABP/PPG cardiac cycle second derivative Bpeak duration with respect to Dpeak to the ABP/PPG cardiac cycle second derivative Apeak duration with respect to Dpeak
816	ABP/PPG_D_RT_SD_Bpeak_wrtEpeak_ABP/PPG_D_SD_Apeak_wrtDpeak	The ratio of the ABP/PPG cardiac cycle second derivative Bpeak duration with respect to Epeak to the ABP/PPG cardiac cycle second derivative Apeak duration with respect to Dpeak
817	ABP/PPG_D_RT_SD_Cpeak_wrtDpeak_ABP/PPG_D_SD_Apeak_wrtDpeak	The ratio of the ABP/PPG cardiac cycle second derivative Cpeak duration with respect to Dpeak to the ABP/PPG cardiac cycle second derivative Apeak duration with respect to Dpeak
818	ABP/PPG_D_RT_SD_Cpeak_wrtEpeak_ABP/PPG_D_SD_Apeak_wrtDpeak	The ratio of the ABP/PPG cardiac cycle second derivative Cpeak duration with respect to Epeak to the ABP/PPG cardiac cycle second derivative Apeak duration with respect to Dpeak
819	ABP/PPG_D_RT_SD_Dpeak_wrtEpeak_ABP/PPG_D_SD_Apeak_wrtDpeak	The ratio of the ABP/PPG cardiac cycle second derivative Dpeak duration with respect to Epeak to the ABP/PPG cardiac cycle second derivative Apeak duration with respect to Dpeak
820	ABP/PPG_D_RT_SD_Bpeak_wrtCpeak_ABP/PPG_D_SD_Apeak_wrtEpeak	The ratio of the ABP/PPG cardiac cycle second derivative

		Bpeak duration with respect to Cpeak to the ABP/PPG cardiac cycle second derivative Apeak duration with respect to Epeak
821	ABP/PPG_D_RT_SD_Bpeak_wrtDpeak_ABP/PPG_D_SD_Apeak_wrtEpeak	The ratio of the ABP/PPG cardiac cycle second derivative Bpeak duration with respect to Dpeak to the ABP/PPG cardiac cycle second derivative Apeak duration with respect to Epeak
822	ABP/PPG_D_RT_SD_Bpeak_wrtEpeak_ABP/PPG_D_SD_Apeak_wrtEpeak	The ratio of the ABP/PPG cardiac cycle second derivative Bpeak duration with respect to Epeak to the ABP/PPG cardiac cycle second derivative Apeak duration with respect to Epeak
823	ABP/PPG_D_RT_SD_Cpeak_wrtDpeak_ABP/PPG_D_SD_Apeak_wrtEpeak	The ratio of the ABP/PPG cardiac cycle second derivative Cpeak duration with respect to Dpeak to the ABP/PPG cardiac cycle second derivative Apeak duration with respect to Epeak
824	ABP/PPG_D_RT_SD_Cpeak_wrtEpeak_ABP/PPG_D_SD_Apeak_wrtEpeak	The ratio of the ABP/PPG cardiac cycle second derivative Cpeak duration with respect to Epeak to the ABP/PPG cardiac cycle second derivative Apeak duration with respect to Epeak
825	ABP/PPG_D_RT_SD_Dpeak_wrtEpeak_ABP/PPG_D_SD_Apeak_wrtEpeak	The ratio of the ABP/PPG cardiac cycle second derivative Dpeak duration with respect to Epeak to the ABP/PPG cardiac cycle second derivative Apeak duration with respect to Epeak
826	ABP/PPG_D_RT_SD_Bpeak_wrtDpeak_ABP/PPG_D_SD_Bpeak_wrtCpeak	The ratio of the ABP/PPG cardiac cycle second derivative Bpeak duration with respect to Dpeak to the ABP/PPG cardiac cycle second derivative Bpeak duration with respect to Cpeak
827	ABP/PPG_D_RT_SD_Bpeak_wrtEpeak_ABP/PPG_D_SD_Bpeak_wrtCpeak	The ratio of the ABP/PPG cardiac cycle second derivative Bpeak duration with respect to Epeak to the ABP/PPG cardiac

		cycle second derivative Bpeak duration with respect to Cpeak
828	ABP/PPG_D_RT_SD_Cpeak_wrtDpeak_ABP/PPG_D_SD_Bpeak_wrtCpeak	The ratio of the ABP/PPG cardiac cycle second derivative Cpeak duration with respect to Dpeak to the ABP/PPG cardiac cycle second derivative Bpeak duration with respect to Cpeak
829	ABP/PPG_D_RT_SD_Cpeak_wrtEpeak_ABP/PPG_D_SD_Bpeak_wrtCpeak	The ratio of the ABP/PPG cardiac cycle second derivative Cpeak duration with respect to Epeak to the ABP/PPG cardiac cycle second derivative Bpeak duration with respect to Cpeak
830	ABP/PPG_D_RT_SD_Dpeak_wrtEpeak_ABP/PPG_D_SD_Bpeak_wrtCpeak	The ratio of the ABP/PPG cardiac cycle second derivative Dpeak duration with respect to Epeak to the ABP/PPG cardiac cycle second derivative Bpeak duration with respect to Cpeak
831	ABP/PPG_D_RT_SD_Bpeak_wrtEpeak_ABP/PPG_D_SD_Bpeak_wrtDpeak	The ratio of the ABP/PPG cardiac cycle second derivative Bpeak duration with respect to Epeak to the ABP/PPG cardiac cycle second derivative Bpeak duration with respect to Dpeak
832	ABP/PPG_D_RT_SD_Cpeak_wrtDpeak_ABP/PPG_D_SD_Bpeak_wrtDpeak	The ratio of the ABP/PPG cardiac cycle second derivative Cpeak duration with respect to Dpeak to the ABP/PPG cardiac cycle second derivative Bpeak duration with respect to Dpeak
833	ABP/PPG_D_RT_SD_Cpeak_wrtEpeak_ABP/PPG_D_SD_Bpeak_wrtDpeak	The ratio of the ABP/PPG cardiac cycle second derivative Cpeak duration with respect to Epeak to the ABP/PPG cardiac cycle second derivative Dpeak duration with respect to Cpeak
834	ABP/PPG_D_RT_SD_Dpeak_wrtEpeak_ABP/PPG_D_SD_Bpeak_wrtDpeak	The ratio of the ABP/PPG cardiac cycle second derivative Dpeak duration with respect to Epeak to the ABP/PPG cardiac cycle second derivative Bpeak duration with respect to Dpeak

835	ABP/PPG_D_RT_SD_Cpeak_wrtDpeak_ABP/PPG_D_SD_Bpeak_wrtEpeak	The ratio of the ABP/PPG cardiac cycle second derivative Cpeak duration with respect to Dpeak to the ABP/PPG cardiac cycle second derivative Bpeak duration with respect to Epeak
836	ABP/PPG_D_RT_SD_Cpeak_wrtEpeak_ABP/PPG_D_SD_Bpeak_wrtEpeak	The ratio of the ABP/PPG cardiac cycle second derivative Cpeak duration with respect to Epeak to the ABP/PPG cardiac cycle second derivative Dpeak duration with respect to Epeak
837	ABP/PPG_D_RT_SD_Dpeak_wrtEpeak_ABP/PPG_D_SD_Bpeak_wrtEpeak	The ratio of the ABP/PPG cardiac cycle second derivative Dpeak duration with respect to Epeak to the ABP/PPG cardiac cycle second derivative Bpeak duration with respect to Epeak
838	ABP/PPG_D_RT_SD_Cpeak_wrtEpeak_ABP/PPG_D_SD_Cpeak_wrtDpeak	The ratio of the ABP/PPG cardiac cycle second derivative Cpeak duration with respect to Epeak to the ABP/PPG cardiac cycle second derivative Cpeak duration with respect to Dpeak
839	ABP/PPG_D_RT_SD_Dpeak_wrtEpeak_ABP/PPG_D_SD_Cpeak_wrtDpeak	The ratio of the ABP/PPG cardiac cycle second derivative Dpeak duration with respect to Epeak to the ABP/PPG cardiac cycle second derivative Cpeak duration with respect to Dpeak
840	ABP/PPG_D_RT_SD_Dpeak_wrtEpeak_ABP/PPG_D_SD_Cpeak_wrtEpeak	The ratio of the ABP/PPG cardiac cycle second derivative Dpeak duration with respect to Epeak to the ABP/PPG cardiac cycle second derivative Cpeak duration with respect to Epeak

27. Frequency domain features (841-852)

841	ABP/PPG_AM_P1	The amplitude of the first peak observed in the FFT of the ABP/PPG cardiac cycle
842	ABP/PPG_AM_P2	The amplitude of the second peak observed in the FFT of the ABP/PPG cardiac cycle
843	ABP/PPG_AM_P3	The amplitude of the third peak observed in the FFT of the ABP/PPG cardiac cycle
844	ABP/PPG_F_F1	The frequency corresponding to the first peak in the FFT of the ABP/PPG cardiac cycle
845	ABP/PPG_F_F2	The frequency corresponding to the second peak in the FFT of the ABP/PPG cardiac cycle
846	ABP/PPG_F_F3	The frequency corresponding to the third peak in the FFT of the ABP/PPG cardiac cycle
847	ABP/PPG_RT_ AM_P1_P2	The ratio of the first peak amplitude to that of the second peak amplitude in the FFT of the ABP/PPG cardiac cycle
848	ABP/PPG_RT_ AM_P1_P3	The ratio of the first peak amplitude to that of the third peak amplitude in the FFT of the ABP/PPG cardiac cycle
849	ABP/PPG_RT_ AM_P2_P3	The ratio of the second peak amplitude to that of the third peak amplitude in the FFT of the ABP/PPG cardiac cycle
850	ABP/PPG_RT_ F_F1_F2	The ratio of the frequency values of the first peak and the second peak in the FFT of the ABP/PPG cardiac cycle
851	ABP/PPG_RT_ F_F1_F3	The ratio of the frequency values of the first peak and the third peak in the FFT of the ABP/PPG cardiac cycle
852	ABP/PPG_RT_ F_F2_F3	The ratio of the frequency values of the second peak and the third peak in the FFT of the ABP/PPG cardiac cycle
